# Using data on snus use in Sweden to compare different modelling approaches to estimate the population health impact of introducing a smoke-free tobacco product

**DOI:** 10.1186/s12889-019-7714-0

**Published:** 2019-10-29

**Authors:** Smilja Djurdjevic, Laszlo Pecze, Rolf Weitkunat, Frank Luedicke, John Fry, Peter Lee

**Affiliations:** 1 Philip Morris R&D, Philip Morris Products SA, Quai Jeanrenaud 5, CH-2000 Neuchâtel, Switzerland; 2 Route du Midi 1, CH-1723 Marly, Switzerland; 3 Roelee Statistics Ltd, 17 Cedar Road, Sutton, Surrey SM2 5DA UK; 4 P N Lee Statistics and Computing Ltd, 17 Cedar Road, Sutton, Surrey SM2 5DA UK

**Keywords:** Smoking, Snus, Modelling, Attributable risk, Smoke-free tobacco product, Harm reduction, Validation

## Abstract

**Background:**

We have developed an approach for modelling the health impact of introducing new smoke-free tobacco products. We wished to compare its estimates with those of alternative approaches, when applied to snus, used in Sweden for many years.

**Methods:**

Modelling was restricted to men aged 30–79 years for 1980–2009 and to four smoking-related diseases. Mortality data were extracted for Sweden and other European countries. Published data provided Swedish prevalence estimates for combinations of never/former/current smoking and snus use, and smoking prevalence estimates for other European countries. Approach 1 compares mortality in Sweden and in other countries with a smoking prevalence similar to Sweden’s prevalence of combined smoking/snus use. Approaches 2 and 3 compare mortality in Sweden with hypothetical mortality had snus users smoked. Approach 3 uses our health impact model, individuals starting with the tobacco prevalence of Sweden in 1980. Tobacco histories during 30-year follow-up were then estimated using transition probabilities, with risk derived using a negative exponential model. Approach 2 uses annual tobacco prevalence estimates coupled with estimates of relative risk of current and former smokers regardless of history. The main applications of Approaches 2 and 3 assume that only smoking affects mortality, though sensitivity analyses using Approach 3 allow for risk to vary in snus users and dual users.

**Results:**

Using Approach 2, estimated mortality increases in Sweden in 1980–2009 had snus not been introduced were: lung cancer 8786; COPD 1781; IHD 10,409; stroke 1720. The main Approach 3 estimates were similar (7931, 1969; 12,501; 1901). They decreased as risk in snus users and dual users increased. Approach 1 estimates differed wildly (77,762, 32,538; 77,438; 76,946), remaining very different following correction for differences between Sweden and the comparison countries in non-smoking-related disease mortality.

**Conclusions:**

Approach 1 is unreliable, accounting inadequately for non-tobacco factors affecting mortality. Approaches 2 and 3 provide reasonably similar approximate estimates of the mortality increase had snus not been available, but have differing advantages and disadvantages. Only Approach 3 considers tobacco history, but develops histories using tobacco transition probabilities, which is possibly less reliable than using estimated tobacco prevalences at each follow-up year.

## Background

While quitting tobacco and nicotine altogether is clearly the best way for cigarette smokers to reduce their disease risk, many will not quit. If cigarette smokers who would otherwise not quit would switch to a smoke-free tobacco product (SFTP), this could be helpful from a harm reduction standpoint, especially if the reduction in risk inherent to their chosen product is substantial. In this regard, various SFTPs have recently been developed, notably e-cigarettes and heat-not-burn products. While evidence from biomarkers of exposure and short-term clinical tests shows that switching to these products (such as a heat-not-burn product marketed as IQOS) presents less risk than continued smoking, there is currently no reliable evidence of benefit from epidemiological prospective cohort or case-control studies, even though some of these products have been on the market for over 5 years. Nevertheless, there is a need to estimate the population health impact of introducing SFTPs, partly to satisfy the requirements of the United States Food and Drug Administration’s draft guidelines [1] to applicants seeking to introduce such products into the U.S. market under a modified-risk tobacco product marketing order.

In 2015, we developed an approach to assess the population health impact of introducing a SFTP [2], leading to estimates of the impact of such introduction in the US on mortality from the four main smoking-related diseases (SRDs) – lung cancer, chronic obstructive pulmonary disease (COPD), ischaemic heart disease (IHD) and stroke [3, 4]. The Population Health Impact Model (PHIM) we developed involves starting in a given year with a prevalence of smoking (never, current, former – by time quit) that is representative of the population studied. Individual tobacco histories are then updated annually over a defined period using estimated probabilities of switching between never/current/former cigarette smoking where the SFTP is not introduced (the “Null” or “Historical” Scenario), or switching between never/current cigarettes only/current SFTP only/current dual use/former smoking where it is (the “Alternative” or “Hypothetical” Scenario). Based on (a) the tobacco histories obtained, (b) published estimates of the relative risk for current smoking and of the quitting half-life (the time it takes to halve the excess risk associated with continued smoking) [3], and (c) estimates of the effective dose (compared to cigarette only smokers) for SFTP only and for dual use, a negative exponential model (NEM) [3, 5] is then used to estimate each individual’s risk of each disease at each year of follow-up. Combining these estimates with national data on population size and mortality, one can then derive the expected numbers of deaths in each Scenario and hence the reduction in the number of deaths related to introducing the SFTP.

Due particularly to limited data on the switching probabilities in the Hypothetical Scenario and on the effective dose for exclusive SFTP use and for dual use, such estimates are clearly imprecise. Furthermore, the modelling approach used to estimate the reduction in deaths following SFTP introduction cannot be validated against observed changes in mortality for recently introduced SFTPs.

However, extensive evidence on uptake and risk is available for one SFTP that has been on the market for many years. This is Swedish moist snuff, commonly referred to as “snus”, which has been marketed in Sweden for over 100 years [6]. Despite published meta-analyses [7–9] showing excess risks of the main SRDs to be much lower than those from cigarette smoking, it is rarely used in most other European countries, as snus is banned for sale outside Sweden in the European Community.

Here we use three approaches to estimate the increase in the number of deaths from the four major diseases that would have occurred in Sweden over the 30-year period starting in 1980 if snus had not been available.

“Approach 1” compares the historical number of deaths in Sweden with the hypothetical number that would have occurred if Sweden had the mortality rates of other European countries with a prevalence of smoking relatively similar to the prevalence of overall tobacco use (for smoking and snus combined) in Sweden.

“Approach 2” compares the historical number of deaths in Sweden with the hypothetical number that would have occurred if current and former snus users had actually been current and former smokers.

While these two approaches do not use the PHIM, “Approach 3” uses it to make a similar comparison to Approach 2.

All three approaches provide estimates of the increase in mortality from the four diseases that would have occurred in Sweden if snus had not been introduced.

## Methods

### Data used

All data considered were for males and for the period 1980–2009. Effects on mortality were restricted to those aged 30–79 years.

Annual data by 5 year age groups on population size for Sweden, and in the case of Approach 2 for other European countries, came from the United Nations website [10].

Annual data by 5 year age groups for Sweden and other European countries on deaths from lung cancer, COPD, IHD and stroke (“the four diseases”), all SRDs, all non-smoking-related diseases (NSRD), and all causes combined came from the WHO database [11]. The definition of SRDs was based on that defined by Tachfouti et al. [12] with minor modifications, as described in detail in Additional file 1.

Published data were used to estimate prevalence for Sweden for nine groups of tobacco use, representing each combination of the 3 × 3 matrix smoker (never/former/current) x snus user (never/former/current), separately for 20 age groups (15, 16, 17, 18, 19, 20, 21–24, 25–29, 30–34 … 80–84 and 85+). Details of the source publications and the methods used to estimate the prevalences are given in Additional file 2.

Other data used were specific to particular approaches and the sources are described below.

### Approach 1

In males in 1980–2009 in European countries other than Sweden tobacco users were nearly all cigarette smokers. Approach 1 estimates the effect of snus use in Swedish males based on a comparison of their mortality rates with those seen in combined male data from other European countries with an overall prevalence of current tobacco use similar to that in Sweden. Comparisons were made annually from 1980 to 2009 of mortality from the four diseases for the combined age group 30–79 years with age-standardization to the European standard population (ESP 1976) [13].

For Sweden the overall prevalence of current tobacco use in each year for each age group was estimated by combining all those estimated prevalences for the nine tobacco use groups described above which involved current smoking and/or current snus use.

Annual data on the prevalence of current smoking for European countries was extracted from the Global Burden of Disease Study 2015 [14], although data for countries with an average population size less than 500,000, with sales of tobacco products other than manufactured cigarettes or snus greater than 35%, or with markedly incomplete mortality data were not considered. Countries were considered similar to Sweden if the average annual absolute difference between their prevalence and that in Sweden for 1980–2009 was less than 4%.

For each 5 year age group, annual mortality rates (per 100,000) for each of the four diseases and countries were then calculated by dividing estimates of the numbers of deaths by estimates of the population size [10] and multiplying by 100,000. Weights calculated from the ESP 1976 [13] for each 5 year age-group then provided the age-standardized mortality rates. For each disease, year and country, these were divided by the corresponding value for Sweden to give the disease specific mortality rate ratios (normalized to Swedish data).

For each disease, year and country, the number of deaths in Sweden occurring at age 30–79 years was then multiplied by the corresponding rate ratio to obtain the hypothetical number of deaths that would have occurred with the mortality rate of the comparison country. The mean of these hypothetical numbers of deaths was then compared with the historical number of deaths in Sweden.

As mortality in Sweden may be lower than that in other countries for reasons other than tobacco use, adjusted hypothetical numbers of deaths were also calculated. The method was as described in the previous paragraph except that the year- and country-specific rate ratios used to multiply deaths in Sweden for each of the four diseases were each divided by the corresponding rate ratio calculated for deaths from all NSRD.

The differences between the hypothetical numbers (adjusted or unadjusted) of deaths in Sweden and the historical numbers are indicators of the increase in deaths that would have occurred in Sweden had snus not been on the market.

### Approach 2

As for Approach 1, Approach 2 estimates a hypothetical mortality rate for Swedish males assuming that snus was not available and that those who had used snus smoked instead. Approach 2 compares the historical numbers of deaths in males aged 20–79 years from a specified disease that occurred in Sweden in a defined year (A) with the hypothetical number if snus users had smoked cigarettes instead (B), the difference (B-A) being an estimate of the increase in deaths that would have occurred if snus had not been available.

The estimation required annual age-specific data for Swedish males on tobacco use prevalence for the nine categories as described earlier, population size [10] and numbers of deaths for the diseases of interest [11]. It also required estimates of relative risks of the four diseases for current and former smokers. These estimates, shown in Table 1, came from published meta-analyses [3, 15, 16]. Table 1 also includes estimates of relative risks for current snus use, again taken from a published meta-analysis [8]. As can be seen the relative risks for current snus use were all non-significant and close to 1, so it was decided to estimate mortality assuming that disease risk depends only on smoking.

**Table 1:** Relative risks of tobacco related disease in Swedish males

	Age range	Relative Risk (95% CI) Current smoker	Relative Risk (95% CI) Former smoker	Relative Risk (95% CI) Current snus
Lung Cancer	Any	8.68 (7.14–10.54)	2.62 (2.01–3.42)	0.80 (0.60–1.06)
COPD	Any	3.31 (2.80–3.92)	1.99 (1.76–2.25)	0.80 (0.40–1.60)
IHD	to 54 55 to 64 65 to 74 75 to 79 Any	3.38 (2.92–3.91) 2.32 (2.05–2.62) 1.70 (1.56–1.86) 1.27 (1.21–1.33)	1.36 (1.21–1.53) 1.38 (1.22–1.55) 1.25 (1.16–1.34) 1.16 (1.08–1.25)	1.01 (0.91–1.12)
Stroke	to 54 55 to 64 65 to 74 75 to 79 Any	2.48 (1.94–3.17) 2.13 (1.93–2.34) 1.39 (1.23–1.58) 1.06 (0.96–1.17)	1.10 (0.90–1.34) 1.17 (1.01–1.36) 1.15 (1.04–1.26) 1.00 (0.89–1.12)	1.04 (0.92–1.17)

Table 1 presents relative risks for lung cancer, COPD, IHD and stroke for current and former smokers compared to never smokers and for current snus users relative to never users. Estimates for IHD and stroke for smoking are given by age, but other relative risks are assumed to be independent of age. The estimates for smoking come from published meta-analyses for lung cancer [15], for COPD [16], and for IHD and stroke [3]. The estimates for snus use come from another published meta-analysis [8]

If one subdivides the population into nine groups, one can compare risk in the alternative situations, as summarized in Table 2. Note that death rates in the historical situation (A) and hypothetical situation (B) are identical in six of the nine groups, differing only for groups 4, 7 and 8.

**Table 2:** Estimating death rates in Approach 2

	Historical tobacco habits		Death rate in situation		
Group	Smoking	Snus use	A	B	Difference
1	Never	Never	N	N	None
2	Never	Former	N	F	Higher in B
3	Never	Current	N	C	Higher in B
4	Former	Never	F	F	None
5	Former	Former	F	F	None
6	Former	Current	F	C	Higher in B
7	Current	Never	C	C	None
8	Current	Former	C	C	None
9	Current	Current	C	C	None

For the nine tobacco use groups, Table 2 shows the death rates that would apply in situation A, which concerns the historical number of deaths in males from a specified disease that did occur in Sweden in a defined year, and in situation B, which concerns the hypothetical number that would have occurred in that year if snus users had smoked cigarettes instead. N is the death rate in never smokers, F is that in former smokers and C is that in current smokers

The estimation, which was carried out separately for different 5 year age groups, with the results then combined over age group, requires estimates for each year of the population size in each group, N, and of the total number of deaths from the disease of interest, D.

Given the proportions in the nine groups, (P_i_, i = 1 ….. 9) and the relative risk of disease for former smokers and current smokers compared to never smokers, R_F_ and R_C_ we first estimated the death rate in never smokers, U, from the formula

$$ U=D/\Big[N\ \left({P}_1+{P}_2+{P}_3+{R}_F\left({P}_4+{P}_5+{P}_6\right)+{R}_C\left({P}_7+{P}_8+{P}_9\right)\right] $$

The number of deaths in each group in situation A was then obtained by multiplying N*U*P_i_ by 1 for groups 1 to 3, R_F_ for groups 4 to 6 and by R_C_ for groups 7 to 9. The number of deaths in each group in situation B is the same as that in situation A in six groups (1, 4, 5, 7, 8, 9) but is multiplied by R_F_ for group 2, R_C_ for group 3 and R_C_/R_F_ for Group 6.

### Approach 3

The methodology of the PHIM, which was designed to assess the population-level health impact of marketing a SFTP, has already been summarized in the background section and is described in more detail elsewhere [2, 3]. The application of the PHIM used in Approach 3 involves comparison of mortality from the four main SRDs in the historical scenario (SNUS) in which snus is present and the hypothetical scenario (NO-SNUS) in which it is not. In each scenario tobacco transition probabilities (TTPs) determine the rate at which individuals change tobacco groups. Based on the tobacco use histories built up, the relative risks of each disease are then estimated for each individual, and are used to determine the number of deaths attributable to tobacco. The difference between the estimated numbers of deaths for the two scenarios (NO-SNUS minus SNUS) then provides the increase in mortality if snus had not been available.

In Approach 3, simulated samples of 100,000 males start in 1980 with a distribution of smoking habits consistent with the prevalence in Swedish males in that year.

In the SNUS scenario individuals start in five groups – never tobacco (representing the combination of groups 1 + 2 of the original nine groups shown in Table 2), current cigarettes only (7 + 8), current snus only (3), current dual use (9) and former tobacco (4 + 5 + 6). This was based on two assumptions. One was that there was no increase in risk associated with former use of snus, consistent with epidemiological evidence that exclusive snus use is associated with little or no increase in the incidence of the smoking attributable diseases studied [8, 9]. This suggests that any risk of former snus use can be ignored, so indicating that groups 1 + 2, 4 + 5 and 7 + 8 could each be combined as having equivalent risk. Evidence that current snus users who formerly smoked (“switchers”) have risks very similar to those of never users who quit smoking (“quitters”) [17] also justified the decision to count those originally in group 6 as having equivalent risk to the other former cigarette smoking groups 4 and 5.

In the NO-SNUS scenario, individuals start in three groups again based on the original nine groups – never cigarettes (1), former cigarettes (2 + 4 + 5) and current cigarettes (3 + 6 + 7 + 8 + 9). Thus, this scenario included as former smokers all those who had used either or both products but did not currently use them, and as current smokers all those who were current users of either or both product. Never smokers included only those who had never used either product. Effectively it was assumed that cigarette smoking totally replaced snus use.

During each year of the 30 year follow-up period (1980–2009), individuals can switch groups according to defined TTPs. In the SNUS scenario there are 15 TTPs, three relating to initiation, three relating to quitting, three relating to re-initiation and six relating to switching. Thus, for example, individuals may initiate or re-initiate to each of the three current use groups, or may quit from each of them. In the NO-SNUS scenario there are three TTPs, representing initiation, quitting and re-initiation. The values of the TTPs used, which are age dependent, are given in Additional file 3 which also provides further details of the methodology. Note that the TTPs for initiation and re-initiation in the NO-SNUS scenario are the same as the three TTPs in the SNUS scenario, while the TTPs for quitting in NO-SNUS are the same as the three identical TTPs for quitting in SNUS.

Comparison is between mortality in the two scenarios over the 30 year follow-up period. Note that any individual reaching age 80 drops out of the population, so by the end of follow-up smoking prevalence refers to those aged 40–79. The model requires the disease- and age-specific estimates of the relative risk associated with continued smoking, and also requires estimates of the quitting half-life, the time after quitting when the increase in relative risk associated with smoking has halved. These estimates, derived from published meta-analyses, are provided in our earlier paper [3], which clarifies the sources used.

The model also requires estimates of the “relative exposure” (RE) corresponding to the current tobacco use pattern. This takes the value 0 for an individual not using tobacco (a never or former user), 1 for a current cigarette smoker, f for a current SNUS user (the f-factor), and g for a dual user (the g-factor), a dual user being an individual whose tobacco use pattern consists of a substantial use of both cigarettes and snus. The results shown in Table 1 suggest that the f-factor is close to zero and the g-factor is close to 1. In Approach 2 and in the main analysis using Approach 3, we assume that f = 0 and g = 1. In Approach 3 we also conduct sensitivity analyses, with g = 1.0 and f = 0.1 or 0.2, with f = 0.0 and g = 0.9, 0.8 or 0.5, and with f = 0.1 and g = 0.9 or 1.1.

For each of the four major SRDs and for each five-year age group and each year of follow-up the PHIM estimates the mean relative risk for each of the two scenarios. The number of deaths occurring in the SNUS scenario is the number that actually occurred, while the number occurring in the NO-SNUS scenario can be obtained by multiplying this number by the ratio of the mean relative risks in the NO-SNUS and SNUS scenarios. The difference between these two numbers of deaths is then the required increase in deaths for that disease, age group and year.

The estimated increases in deaths for each disease so far described take no account of the reduced population size that would have existed in the hypothetical scenario. For the main analysis, with f = 0.0 and g = 1.0, we also present survival-adjusted estimates, using methodology previously described [2].

### Years of life lost

For each disease and each year of follow-up, separately for the historical and hypothetical scenario, Approaches 2 and 3 both generate estimates of the number of deaths occurring in each year of follow-up for each of the 5 year age groups from 30 to 34 to 75–79. These estimates were also converted to numbers of years of life lost before age 80, taking the midpoints of the age groups as 32.5, 37.5, .. 77.5. Thus, those dying at age 30–34 would lose 80–32.5 = 47.5 years, for example.

## Results

### Tobacco use prevalence in Swedish male population

**Fig. 1:**
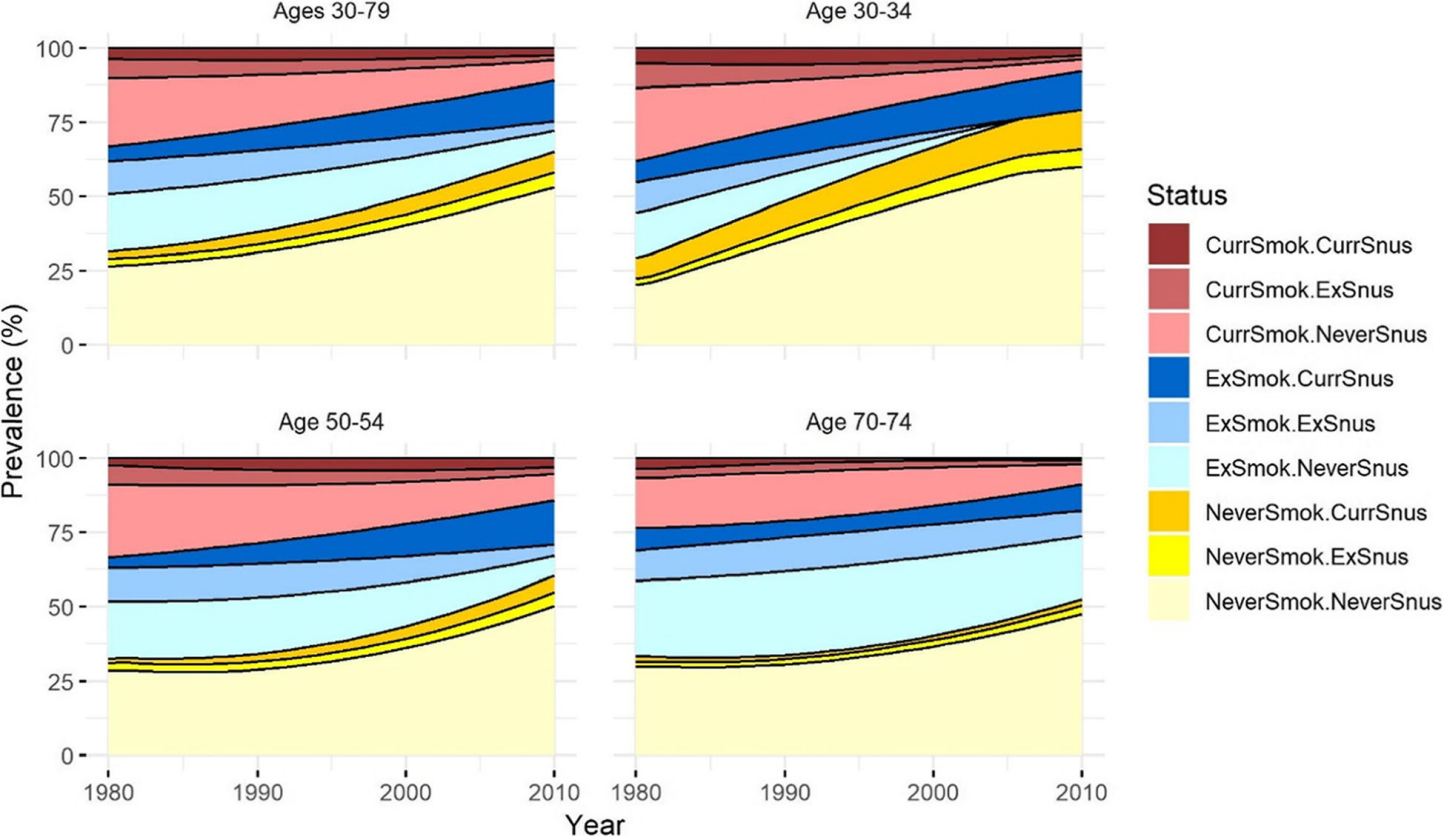
Distribution of tobacco use status in Swedish males by year and age. Figure 1 shows the distribution of the nine subcategories of tobacco use status in Swedish males by year from 1980 to 2009 for all ages (30–79 years), and for three selected age groups

Figure 1 shows the distribution of prevalence of tobacco use in Swedish males for the nine categories by year for the age-groups 30–79, 30–34, 50–54 and 70–74 years. Cigarette smoking prevalence, standardized to age 30–79 years, declined from 33% in 1980 to 11% in 2009 (red areas). Over the same period, the prevalence of snus use including snus and cigarette dual use increased from 11 to 23% (dark yellow, dark blue and dark red areas). Fewer former smokers and former snus users are observed at younger ages, as expected. Nevertheless, the overall prevalence of former tobacco use also decreased during the observed period. Consequently, the subcategory of never tobacco users increased with time.

### Approach 1

There were seven other European countries where, over the period 1980–2009, the mean absolute difference between their age-standardized prevalence of current smoking in males aged 30–79 years differed from the prevalence of current tobacco use for Sweden by less than 4%. These countries, with the mean absolute differences from Sweden shown in parentheses, were Spain (1.34%), Hungary (1.46%), Lithuania (2.16%), Czech Republic (2.95%), Poland (3.09%), Denmark (3.36%) and Slovakia (3.93%).

**Fig. 2:**
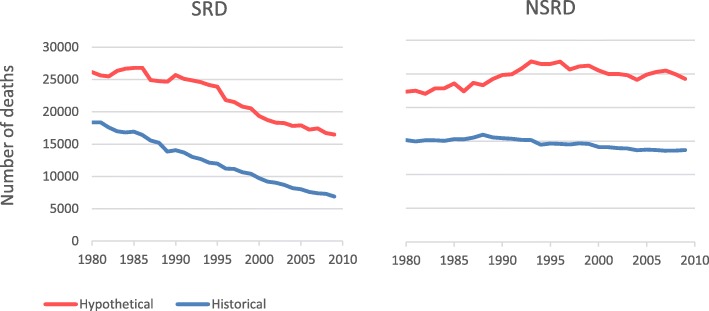
Comparison of SRD and NSRD deaths in Sweden and in the seven comparison countries. For the period 1980–2009, Fig. 2 compares the number of deaths in each year from all SRD and all NSRD occurring in Swedish males aged 30–79 years (historical data) with those that would have occurred if they had the average mortality of seven other European countries with a prevalence of cigarette smoking similar to the prevalence of tobacco use seen in Sweden (hypothetical data)

Figure 2 shows (blue lines) the historical number of deaths occurring each year in Swedish males aged 30–79 years from all SRD combined and from all NSRD. Also shown (red lines) are the hypothetical number of deaths that would have occurred if Swedish males had the average rates seen in the seven countries with similar rates of tobacco use. The hypothetical rates are higher, for SRD, consistent with the beneficial effect of Swedish snus on population health shown in Table 1. However, they are also higher for NSRD, supporting the need for adjustment of the simple comparison of historical and hypothetical rates for the four diseases of specific interest (lung cancer, COPD, IHD and stroke).

**Fig. 3:**
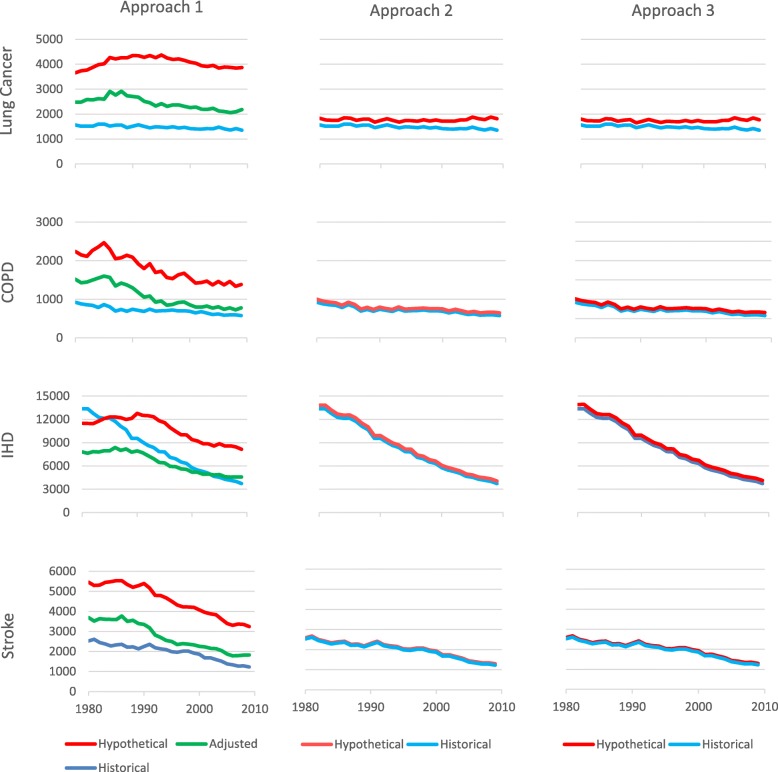
Comparison of historical deaths in Sweden with hypothetical deaths as estimated by the three Approaches. For the period 1980–2009, Fig. 3 compares the historical number of deaths in each year occurring in Swedish males aged 30–79 years with the hypothetical number of deaths estimated by the three Approaches. For Approach 1, the hypothetical numbers are also shown with adjustment for the lower mortality from all NSRD in Sweden than in the seven comparison countries. For Approach 3, the hypothetical numbers assume there is no effect of snus on mortality (f factor = 0.0, g factor = 1.0)

Figure 3 similarly shows the historical numbers of deaths occurring in Swedish males aged 30–79 years from the four specific diseases and the hypothetical numbers estimated from the three Approaches. For Approach 1, the hypothetical numbers adjusted for the lower rates of NSRD in Sweden are also shown (green lines). For approach 3, the hypothetical numbers assume that snus use has no effect on risk. It can be seen that, as compared to Approach 1, the hypothetical numbers are much closer to the historical numbers for Approaches 2 and 3.

### Approach 2

In this approach, deaths actually occurring in Sweden are compared with those that would have occurred if current and former snus users had been current and former cigarette smokers, with a resultant increase in risk. As shown in Fig. 3, we observe increased mortality rates in the hypothetical scenario for each disease. The elevation is clear for lung cancer and COPD, but still evident for IHD and stroke.

### Approach 3

**Fig. 4:**
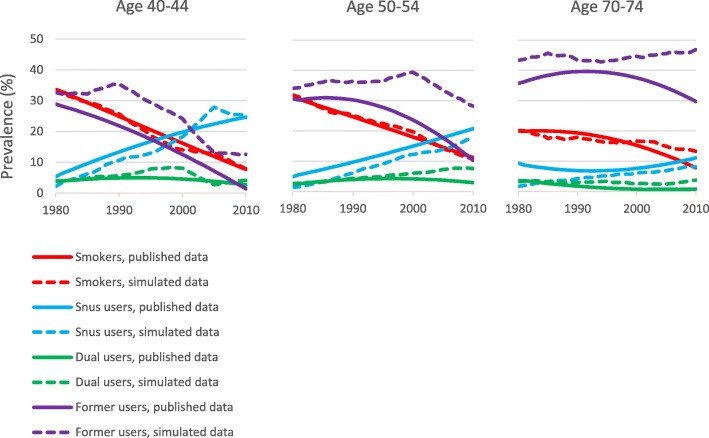
Approach 3. Comparison of published and PHIM simulated tobacco prevalence where snus is used. For the period 1980–2010 and for three age groups, Fig. 4 compares tobacco use prevalence in Swedish males based on published data with that estimated from PHIM simulations for the historical scenario, where snus is used. The category “Former users” comprises former smokers, former snus users and former dual users

**Fig. 5:**
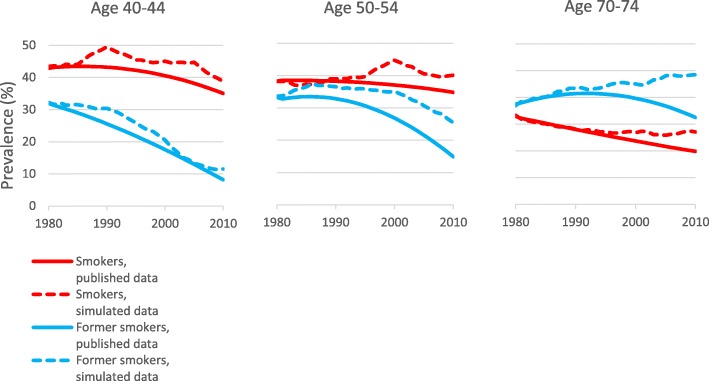
Approach 3. Comparison of published and PHIM simulated smoking prevalence where snus is not used. For the period 1980–2010 and for three age groups, Fig. 5 compares smoking prevalence in Swedish males based on published data with that estimated from PHIM simulations for the historical scenario, where snus is not used

Figure 4 (historical scenario) and Fig. 5 (hypothetical scenario) compare the tobacco use prevalence estimates for Sweden derived as described in Additional file 2 with those estimated by the PHIM based on the initial prevalences and the TTPs. The correspondence between the pairs of estimates for current smokers, snus users, and dual users appears quite reasonable. However, the prevalence of former use of either product is overestimated by the PHIM simulations.

**Fig. 6:**
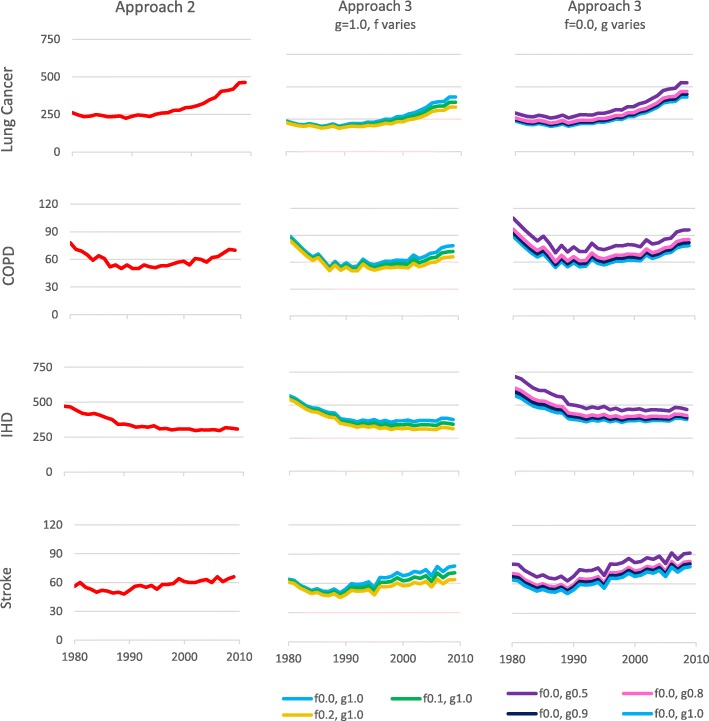
Comparison of estimated increases in deaths in Approach 2 and in Approach 3 with varying f- and g-factors. For the period 1980–2009, Fig. 6 compares the increases in deaths that would have occurred in Swedish males had snus not been introduced, as estimated using Approach 2 (column 1) and in Approach 3 using PHIM simulations where the f-factor varies, with the g-factor fixed at 1.0 (column 2), or where the g-factor varies, with the f-factor fixed at 0.0 (column 3)

In one set of analyses comparing the historical and hypothetical scenarios, the g-factor (relative exposure for dual users) was fixed at 1, but the f-factor (relative exposure for snus only users) varied with values of 0, 0.1 and 0.2. In another set, analyses were carried out with the f-factor fixed at 0, but with the g-factor varying with values of 0.9, 0.8 and 0.5. Figure 6 compares the increases in deaths that would have occurred in the hypothetical scenario for these different Approach 3 analyses as well as showing the corresponding estimates using Approach 2.

The estimates from Approach 3 with f = 0 are somewhat lower over the whole follow-up period than those from Approach 2 for lung cancer, and somewhat higher for the other three diseases. As the f-factor increases the estimates from Approach 3 decline for all four diseases, so getting closer to those from Approach 2 for COPD, IHD and stroke and less close for lung cancer. As the g-factor decreases, the estimates from Approach 3 increase for all four diseases, so getting less close to those from Approach 2 for COPD, IHD and stroke. For lung cancer decreasing the g-factor leads to the increase rising to exceed that from Approach 2.

Comparisons of the increases in deaths associated with unavailability of snus from the different approaches are summarized in Table 3, based on the period 1980–2009. They indicate that for lung cancer and COPD the highest estimates are from Approach 1 and the lowest from Approach 3. For IHD, the adjusted result for Approach 1 is unreliable (the occurrence of IHD being extremely high in Sweden compared to other countries, mostly due to non-smoking attributable cases). Nevertheless, Approach 2 and Approach 3 provide quite consistent results. For stroke, Approach 1 shows a very large increase in deaths compared to the other approaches.

**Table 3:** Increase in deaths in Sweden if snus had not been introduced – summary of results

	Lung cancer	COPD	IHD	Stroke
Approach 1	77,762	32,538	77,438	76,946
Approach 1 (Adjusted)	28,535	11,265	−50,597	23,146
Approach 2	8786	1781	10,409	1720
PHIM f = 0.0, g = 1.0	7931	1969	12,501	1901
PHIM f = 0.0, g = 1.0 (survival adjusted)	7573	1781	10,907	1443
PHIM f = 0.1, g = 0.9	7849	1970	12,386	1865
PHIM f = 0.1, g = 1.0	7423	1868	11,784	1781
PHIM f = 0.1, g = 1.1	7004	1767	11,186	1697
PHIM f = 0.2, g = 1.0	6932	1770	11,082	1663
PHIM g = 0.9, f = 0.0	8367	2072	13,110	1986
PHIM g = 0.8, f = 0.0	8812	2175	13,724	2072
PHIM g = 0.5, f = 0.0	10,198	2493	15,597	2333

Table 3 shows the increase in the number of deaths that would have occurred in the years 1980–2009 in Swedish males aged 30–79 years if snus had not been introduced, as estimated by the different Approaches

Table 3 also provides estimates of increases in deaths for two analyses in which both f and g varied from the values used in the main analyses. The estimates for f = 0.1, g = 0.9 were very similar to those for the main analysis where f = 0.0 and g = 1.0. Similarly, estimates for f = 0.1, g = 1.1 were similar to those for f = 0.2 and g = 1.0, supporting the idea that the effect of increasing f approximately cancelled out the effect of decreasing g by a similar amount.

Also shown in Table 3 are results from a survival-adjusted version of the main analysis. Survival adjustment reduced the number of deaths by 4.5% for lung cancer, 9.5% for COPD, 12.8% for IHD and 24.1% for stroke. Compared to the effect of increasing f to 0.2, the reductions seen were less for lung cancer, similar for COPD and IHD, and greater for stroke.

Additional file 4 gives further details concerning the estimated increases in deaths.

Table 4 shows results for years of life lost corresponding to those for deaths in Table 3. The pattern of results is essentially the same. Each increase in lung cancer deaths is associated with an increase of about 14 years life lost, with corresponding figures of about 10, 17 and 19 years for COPD, IHD and stroke.

**Table 4:** Years of life lost in Sweden if snus had not been introduced – summary of results

	Lung cancer	COPD	IHD	Stroke
Approach 2	113,640	17,212	178,966	33,363
PHIM f = 0.0, g = 1.0	108,295	18,823	211,706	36,046
PHIM f = 0.0, g = 1.0 (survival adjusted)	105,394	17,672	200,717	33,279
PHIM f = 0.1, g = 0.9	105,974	18,695	207,174	35,045
PHIM f = 0.1, g = 1.0	100,229	17,722	197,149	33,480
PHIM f = 0.1, g = 1.1	94,599	16,759	187,228	31,928
PHIM f = 0.2, g = 1.0	92,545	16,642	183,040	30,974
PHIM g = 0.9, f = 0.0	114,230	19,808	221,920	37,636
PHIM g = 0.8, f = 0.0	120,293	20,802	232,247	39,239
PHIM g = 0.5, f = 0.0	139,294	23,845	263,920	44,136

Table 4 shows the increase in the number of years of life lost that would have occurred in the years 1980–2009 in Swedish males aged 30–79 if snus had not been introduced, as estimated by the different methods

## Discussion

In Sweden in 1925, sales of snus represented over two-thirds of total tobacco sales by weight [6]. Though this declined, steadily, to about 20% in 1965, it then increased and currently forms over half of all tobacco sales [6]. There is also evidence of an increasing uptake in Norway in recent years [6]. There is extensive epidemiological evidence that any risks of disease associated with snus use are very much less than those associated with smoking [8].

Here we investigate various approaches [9] for estimating how many more deaths from the four main SRDs there might have been in men in Sweden in the period 1980–2009 if snus had not been available. Attention was restricted to men because snus use by women in Sweden is much less than that in men [6], and also because this paper is mainly intended as a comparison of methodologies.

Three approaches have been used. In the first, Approach 1, we compare the historical number of deaths from the four diseases that occurred in Sweden with the hypothetical number that might have occurred if mortality rates there had been the average of those seen in seven other European countries with an overall prevalence of tobacco use very similar to that seen in Sweden. Because mortality rates in Sweden might also differ from those for the comparison countries for NSRD, we adjusted the hypothetical number of deaths to reflect this. While the difference between the adjusted hypothetical number of deaths and the actual historical number is generally consistent with the reduced risks for snus compared to smoking, interpretation of this difference is far from straightforward. Differences in mortality between Sweden and the comparison countries may arise for various reasons. These may include differences in exposure to risk factors other than smoking, in healthcare, and in diagnosis and detection of disease. Such differences are likely to vary by disease. Because of this, an adjustment based on diseases unrelated to smoking may well be inaccurate, so that even after adjustment the difference between the actual and hypothetical numbers of deaths will not solely reflect the fact that males in the comparison countries do not use snus. Theoretically, it would be possible to use multiple regression models to compare mortality in Sweden with that of a range of other European countries, after adjustment for a range of other factors that affect mortality from the four diseases of interest. However, not only is this beyond the scope of the work described, which was to compare estimates using PHIM with alternatives based on quite simple approaches, we doubt whether it would generate population health impact estimates similar to those from Approaches 2 and 3, which restrict attention to data from Sweden.

In Approach 2, we compare the historical number of deaths occurring in Sweden with the hypothetical number that would have occurred if current snus users who were never or former smokers had been current smokers, and if former snus users who had never smoked had been former smokers (situation B). While Approach 2 seems likely to give a better estimate than Approach 1 of the effect that snus use has had on the mortality of Swedish men, some points should be noted.

The first is the assumption that Swedish men who used snus but did not smoke would have smoked instead if snus was not available. While not readily testable, one can note that over the period 1965–2005, when the combined sales of snuff and cigarettes (by weight) varied little in Sweden, the proportion of snus rose markedly (Table 5), which seems consistent with this assumption.

**Table 5:** Sales of cigarettes and snus in Sweden from 1965 to 2005

	Consumption (tonnes)			Percentage	
Year	Cigarettes	Snus	Total	Cigarettes	Snus
1965	8160	2490	10,650	76.6	23.4
1975	7587	2943	10,530	72.1	27.9
1985	7249	4560	11,809	61.4	38.6
1995	5280	5407	10,687	49.4	50.6
2005	4499	6561	11,060	40.7	59.3

Table 5 shows trends in sales of cigarettes and snus (in tonnes) in Sweden over the period 1965 to 2005, together with the percentage of the total from each. Data from [6]

Secondly, our estimates of the prevalence of smoking and snus use over the 30 year follow-up period may be subject to some error. As is clear from Additional file 2, estimating prevalences for the nine groups representing combinations of never/former/current smoking and never/former/current snus use for each year from a variety of sources was far from straightforward.

A third point is that the analysis does not take account of the theoretical possibility that taking up smoking (as an alternative to using snus) may affect exposure to other risk factors.

Like Approach 2, Approach 3 compares the number of deaths occurring in Sweden with those that would have occurred in the absence of snus. Here, however, this is estimated using the PHIM. In comparing results from Approach 3 with those in Approach 2, various points have to be considered.

First, while Approach 2 estimates deaths occurring at age 30–79 years over the whole follow-up period, individuals in Approach 3 start at age 10–79 years but age during follow-up, so that by the end of the 30 year follow-up period there are none under age 40 years. Since the great majority of deaths from the four diseases occur at age 40 or over, this should make little difference to the comparison.

Second, while in 1980 both Approaches start with the same prevalence of tobacco habits, the prevalences differ in subsequent years. Approach 2 uses prevalence estimates derived from published sources (see Additional file 2) while Approach 3 derives prevalence estimates using TTPs. While these simulated distributions of tobacco habits align approximately with the estimates used in Approach 2, there are some differences, as seen in Figs. 4 and 5. While it would be possible to define a more detailed set of TTPs which change every year so that the distributions agree better, this would be inconsistent with the usual applications of the PHIM, where TTPs for a new SFTP cannot be precisely known. In any case, as noted above, the prevalence of estimates used in Approach 2 may themselves be subject to error. Although the methods used to derive prevalence estimates in the two approaches differ, we do not believe this is the main reason for the variation in the estimated increase in deaths shown in Table 3. This is because the ratio of the estimated increases in the two Approaches varied little between 1980, when the distributions of tobacco were constrained to be the same (Approach 2/Approach 3 1.11 for lung cancer, 0.88 for COPD, 0.83 for IHD and 0.89 for stroke) and 2009 when the differences were most evident (Approach 2/Approach 3 1.10 for lung cancer, 0.90 for COPD, 0.79 for IHD and 0.85 for stroke).

The main reason why the estimates differ between Approach 2 and 3 lies in differences in how the relative risks are calculated. In Approach 2 the relative risks for current and former smokers used, shown in Table 1, are applied assuming that they apply regardless of any aspect of previous smoking history, including duration of smoking or time quit. In contrast, Approach 3 uses the NEM in which the relative risks for current smokers approach the estimates shown in Table 1 with increasing time smoked, while those for former smokers decline from the current smoker relative risk with increasing time quit. Also the NEM takes into account changes in relative risk relating to more complex patterns of smoking history, such as quitting, then re-initiation. Relative risks for short-term quitters may be much higher than assumed in Approach 2, especially for diseases such as lung cancer with a long half-life.

In Approach 2, it is assumed that snus use does not affect risk of the four diseases. This is equivalent to the Approach 3 analyses where the f-factor is set as 0 and the g-factor at 1. As expected, increasing the f-factor increased the number of tobacco-associated deaths in the snus scenario by increasing risk in the group currently using snus only, so decreasing the estimated increase comparing the NO-SNUS and SNUS scenarios. Similarly, decreasing the g-factor decreased tobacco-associated deaths in the SNUS scenario by decreasing risk in the group currently using both products, so increasing the estimated increase comparing the scenarios. As the Approach 2 estimate cannot be considered to be a gold standard, however, one cannot validly determine the most appropriate values of the f- and g-factors by determining those values which produce the estimated increases that are most consistent with those estimated by Approach 2. One must rely on epidemiological evidence to give plausible estimates of the two factors.

The analyses in Approach 2 and most of the analyses in Approach 3 take no account of the reduction in population size that would have occurred if, in Sweden, smoking had replaced snus. We note that this is not unusual in analyses which estimate the change in mortality that would have occurred in a particular year, given the same population and different assumptions. We also present additional results for Approach 3 showing that adjustment for differential mortality between the historical and hypothetical scenarios has an effect that is of the general order of that arising from plausible variations in the f- and g-factors. Since any bias resulting from ignoring changes in population size should similarly affect both Approaches it seems that failure to account for population size would not materially have affected the comparison of Approaches 2 and 3.

## Conclusions

Three approaches have been investigated in an attempt to determine the increase in the number of deaths from lung cancer, COPD, IHD and stroke that might have occurred in Swedish men in 1980–2008 if snus had not been available. Approach 1, which compared death rates in Sweden with those in seven other European countries with a similar prevalence of tobacco use produced very different answers from the other two approaches and must be regarded as unreliable as failing to account properly for a range of factors other than tobacco use. Approaches 2 and 3, which both compare the number of deaths occurring in Sweden with the number that would have occurred if current and former snus users had actually been current and former smokers, produced relatively similar results. Approach 3, which uses the PHIM, allows for relative risks to vary based on a detailed tobacco history, may have advantages over Approach 2, in which fixed relative risks are used for current and former smokers, regardless of tobacco history. However, whereas Approach 2 derives tobacco prevalence estimates at each year of follow-up from published statistics, Approach 3, which was developed to estimate the impact of a new SFTP where future prevalence is unknown, derives the estimates using TTPs which may not be accurately determined. Both Approach 2 and 3 can be regarded as reasonable approximate approaches, with different advantages and disadvantages.

## Supplementary information

**Additional file 1:** Notes on estimating mortality data.

**Additional file 2:** Sources and methodology for estimating prevalence of tobacco use for Sweden.

**Additional file 3:** Additional details for Approach 3.

**Additional file 4:** Fuller details of results from the three approaches.

## Data Availability

The data used on population size, mortality and smoking prevalence are publically available, from the sources described in the paper. The data used on relative risks are shown in Table 1. The data used on the joint distribution of snus use and smoking were derived from the material presented in Additional file 2 and are available on reasonable request, as are files showing full details of the calculations made.

## Abbreviations

COPD: Chronic obstructive pulmonary disease
ESP 1976: European standard population 1976
IHD: Ischaemic heart disease
NEM: Negative exponential model
NSRD: Non-smoking related diseases
PHIM: Population health impact modelling
SFTP: Smoke-free tobacco product
SRD: Smoking-related diseases
TTP: Tobacco transition probability

## References

[1] Food and Drug Administration. Guidance for Industry: Modified Risk Tobacco Product Applications. Draft Guidance. Guidance for Industry: U.S. Department of Health and Human Services Food and Drug Administration Center for Tobacco Products; 2012.

[2] Weitkunat R, Lee PN, Baker G, Sponsiello-Wang Z, González-Zuloeta Ladd AM, Lüdicke F (2015). A novel approach to assess the population health impact of introducing a modified risk tobacco product. Regul Toxicol Pharmacol.

[3] Lee PN, Fry JS, Hamling JF, Sponsiello-Wang Z, Baker G, Weitkunat R (2017). Estimating the effect of differing assumptions on the population health impact of introducing a Reduced Risk Tobacco Product in the USA. Regul Toxicol Pharmacol.

[4] Djurdjevic Smilja, Lee Peter, Weitkunat Rolf, Sponsiello-Wang Zheng, Lüdicke Frank, Baker Gizelle (2018). Modeling the Population Health Impact of Introducing a Modified Risk Tobacco Product into the U.S. Market. Healthcare.

[5] Lee Peter N., Hamling John, Fry John, Forey Barbara (2015). Using the Negative Exponential Model to Describe Changes in Risk of Smoking-Related Diseases following Changes in Exposure to Tobacco. Advances in Epidemiology.

[6] Forey B, Hamling J, Hamling J, Thornton A, Lee P. International smoking statistics. A collection of worldwide historical data. (web edition). Sutton, Surrey: P N Lee Statistics and Computing Ltd; 2006-2016. Available: www.pnlee.co.uk/iss.htm.

[7] Lee PN, Hamling JS (2009). Systematic review of the relation between smokeless tobacco and cancer in Europe and North America. BMC Med.

[8] Lee PN (2011). Summary of the epidemiological evidence relating snus to health. Regul Toxicol Pharmacol.

[9] Lee PN (2013). Epidemiological evidence relating snus to health - an updated review based on recent publications. Harm Reduct J.

[10] United Nations Department of Economic and Social Affairs. World population prospects: the 2012 revision. Excel tables - population data. United Nations, Department of Economic and Social Affairs, Population Division, Population Estimates and Projections Section; 2013. Available: http://esa.un.org/wpp/Excel-Data/population.htm; http://esa.un.org/unpd/wpp/Documentation/pdf/WPP2012_HIGHLIGHTS.pdf; http://www.un.org/en/development/desa/population/publications/pdf/trends/WPP2012_Wallchart.pdf.

[11] World Health Organization. WHO mortality database. 2013. Available: http://www.who.int/healthinfo/statistics/mortality_rawdata/en/index.html; http://www.who.int/healthinfo/statistics/mortality_rawdata/en/index1.html.

[12] Tachfouti N, Raherison C, Obtel M, Nejjari C (2014). Mortality attributable to tobacco: review of different methods. Arch Public Health.

[13] Waterhouse J, Muir C, Correa P, Powell J (1976). Cancer incidence in five continents.

[14] Reitsma Marissa B, Fullman Nancy, Ng Marie, Salama Joseph S, Abajobir Amanuel, Abate Kalkidan Hassen, Abbafati Cristiana, Abera Semaw Ferede, Abraham Biju, Abyu Gebre Yitayih, Adebiyi Akindele Olupelumi, Al-Aly Ziyad, Aleman Alicia V, Ali Raghib, Al Alkerwi Ala'a, Allebeck Peter, Al-Raddadi Rajaa Mohammad, Amare Azmeraw T, Amberbir Alemayehu, Ammar Walid, Amrock Stephen Marc, Antonio Carl Abelardo T, Asayesh Hamid, Atnafu Niguse Tadela, Azzopardi Peter, Banerjee Amitava, Barac Aleksandra, Barrientos-Gutierrez Tonatiuh, Basto-Abreu Ana Cristina, Bazargan-Hejazi Shahrzad, Bedi Neeraj, Bell Brent, Bello Aminu K, Bensenor Isabela M, Beyene Addisu Shunu, Bhala Neeraj, Biryukov Stan, Bolt Kaylin, Brenner Hermann, Butt Zahid, Cavalleri Fiorella, Cercy Kelly, Chen Honglei, Christopher Devasahayam Jesudas, Ciobanu Liliana G, Colistro Valentina, Colomar Mercedes, Cornaby Leslie, Dai Xiaochen, Damtew Solomon Abrha, Dandona Lalit, Dandona Rakhi, Dansereau Emily, Davletov Kairat, Dayama Anand, Degfie Tizta Tilahun, Deribew Amare, Dharmaratne Samath D, Dimtsu Balem Demtsu, Doyle Kerrie E, Endries Aman Yesuf, Ermakov Sergey Petrovich, Estep Kara, Faraon Emerito Jose Aquino, Farzadfar Farshad, Feigin Valery L, Feigl Andrea B, Fischer Florian, Friedman Joseph, G/hiwot Tsegaye Tewelde, Gall Seana L, Gao Wayne, Gillum Richard F, Gold Audra L, Gopalani Sameer Vali, Gotay Carolyn C, Gupta Rahul, Gupta Rajeev, Gupta Vipin, Hamadeh Randah Ribhi, Hankey Graeme, Harb Hilda L, Hay Simon I, Horino Masako, Horita Nobuyuki, Hosgood H Dean, Husseini Abdullatif, Ileanu Bogdan Vasile, Islami Farhad, Jiang Guohong, Jiang Ying, Jonas Jost B, Kabir Zubair, Kamal Ritul, Kasaeian Amir, Kesavachandran Chandrasekharan Nair, Khader Yousef S, Khalil Ibrahim, Khang Young-Ho, Khera Sahil, Khubchandani Jagdish, Kim Daniel, Kim Yun Jin, Kimokoti Ruth W, Kinfu Yohannes, Knibbs Luke D, Kokubo Yoshihiro, Kolte Dhaval, Kopec Jacek, Kosen Soewarta, Kotsakis Georgios A, Koul Parvaiz A, Koyanagi Ai, Krohn Kristopher J, Krueger Hans, Defo Barthelemy Kuate, Bicer Burcu Kucuk, Kulkarni Chanda, Kumar G Anil, Leasher Janet L, Lee Alexander, Leinsalu Mall, Li Tong, Linn Shai, Liu Patrick, Liu Shiwei, Lo Loon-Tzian, Lopez Alan D, Ma Stefan, El Razek Hassan Magdy Abd, Majeed Azeem, Malekzadeh Reza, Malta Deborah Carvalho, Manamo Wondimu Ayele, Martinez-Raga Jose, Mekonnen Alemayehu Berhane, Mendoza Walter, Miller Ted R, Mohammad Karzan Abdulmuhsin, Morawska Lidia, Musa Kamarul Imran, Nagel Gabriele, Neupane Sudan Prasad, Nguyen Quyen, Nguyen Grant, Oh In-Hwan, Oyekale Abayomi Samuel, PA Mahesh, Pana Adrian, Park Eun-Kee, Patil Snehal T, Patton George C, Pedro Joao, Qorbani Mostafa, Rafay Anwar, Rahman Mahfuzar, Rai Rajesh Kumar, Ram Usha, Ranabhat Chhabi Lal, Refaat Amany H, Reinig Nickolas, Roba Hirbo Shore, Rodriguez Alina, Roman Yesenia, Roth Gregory, Roy Ambuj, Sagar Rajesh, Salomon Joshua A, Sanabria Juan, de Souza Santos Itamar, Sartorius Benn, Satpathy Maheswar, Sawhney Monika, Sawyer Susan, Saylan Mete, Schaub Michael P, Schluger Neil, Schutte Aletta Elisabeth, Sepanlou Sadaf G, Serdar Berrin, Shaikh Masood Ali, She Jun, Shin Min-Jeong, Shiri Rahman, Shishani Kawkab, Shiue Ivy, Sigfusdottir Inga Dora, Silverberg Jonathan I, Singh Jasvinder, Singh Virendra, Slepak Erica Leigh, Soneji Samir, Soriano Joan B, Soshnikov Sergey, Sreeramareddy Chandrashekhar T, Stein Dan J, Stranges Saverio, Subart Michelle L, Swaminathan Soumya, Szoeke Cassandra E I, Tefera Worku Mekonnen, Topor-Madry Roman, Tran Bach, Tsilimparis Nikolaos, Tymeson Hayley, Ukwaja Kingsley Nnanna, Updike Rachel, Uthman Olalekan A, Violante Francesco Saverio, Vladimirov Sergey K, Vlassov Vasiliy, Vollset Stein Emil, Vos Theo, Weiderpass Elisabete, Wen Chi-Pan, Werdecker Andrea, Wilson Shelley, Wubshet Mamo, Xiao Lin, Yakob Bereket, Yano Yuichiro, Ye Penpeng, Yonemoto Naohiro, Yoon Seok-Jun, Younis Mustafa Z, Yu Chuanhua, Zaidi Zoubida, El Sayed Zaki Maysaa, Zhang Anthony Lin, Zipkin Ben, Murray Christopher J L, Forouzanfar Mohammad H, Gakidou Emmanuela (2017). Smoking prevalence and attributable disease burden in 195 countries and territories, 1990–2015: a systematic analysis from the Global Burden of Disease Study 2015. The Lancet.

[15] Lee PN, Forey BA, Coombs KJ (2012). Systematic review with meta-analysis of the epidemiological evidence in the 1900s relating smoking to lung cancer. BMC Cancer.

[16] Forey BA, Thornton AJ, Lee PN (2011). Systematic review with meta-analysis of the epidemiological evidence relating smoking to COPD, chronic bronchitis and emphysema. BMC Pulm Med.

[17] Lee PN (2013). The effect on health of switching from cigarettes to snus - a review. Regul Toxicol Pharmacol.

