# Supplementary material for: Using data on snus use in Sweden to compare different modelling approaches to estimate the population health impact of introducing a smoke-free tobacco product
Source: BMC Public Health. 2019 Oct 29;19:1411. doi: 10.1186/s12889-019-7714-0 (PMC6819486; doi:10.1186/s12889-019-7714-0)
Supplement: Supplementary file 1 — Additional file 1: Notes on estimating mortality data. [file 12889_2019_7714_MOESM1_ESM.docx]

Title : “Using data on snus use in Sweden to validate a published modelling approach for estimating the population health impact of introducing a smoke-free tobacco product”

Authors : Smilja Djurdjevic, Laszlo Pecze, Rolf Weitkunat, Frank Luedicke, John Fry and Peter Lee

**SUPPLEMENTARY FILE 1**

Notes on estimating mortality data

For Sweden, and for the seven comparison countries used in Approach 1, Table 1 shows which ICD codes applied in which year, while Table 2 shows the ICD codes used to define lung cancer, COPD, IHD, stroke and other smoking-related diseases.

Based on this information, mortality data for 1980-2009 were extracted for each five-year age group from 30-34 to 75-79 years.

For ICD 9 and ICD 10, the definitions of smoking-related diseases are based on Table 2 of Tachfouti *et al* [1]. That publication did not give codes for smoking-related diseases for ICD 8, but the codes we used are very similar to those used in ICD 9, the only exceptions being that cancers of the pancreas, bladder and kidney (respectively ICD 9 detailed codes 157, 188 and 189) are not counted as smoking-related in the definition in Table 2. This is because, in ICD 8, they formed only part of code ICD A058, which also included a number of other cancers that were not considered smoking-related by Tachfouti *et al* [1]. As ICD 8 was only relevant in two countries for a limited period, and the diseases only formed a part of the total of smoking-related diseases, this inconsistency is likely only to have a minor biasing effect in the Approach 1 analyses.

**Table 1 ICD codes for different countries per year**

| Country | ICD 8 | ICD 9 Basic Tabulation | ICD 9 Detailed Tabulation | ICD 10 Detailed Tabulation |
| --- | --- | --- | --- | --- |
| Czech Republic | - | 1980-1985  (as Czechoslovakia)  1986-1993 |  | 1994-2009 |
| Denmark | 1980-1993 | - | - | 1994-2009 |
| Hungary | - | 1980-1995 | - | 1996-2009 |
| Lithuania |  | 1993-1997 |  | 1998-2009 |
| Poland | - | 1980-1996 | - | 1999-2009 |
| Slovakia | - | 1980-1991  (as Czechoslovakia) | 1992-1993 | 1994-2009 |
| Spain | - | 1980-1996 | 1997-1998 | 1999-2009 |
| Sweden | 1980-1986 | 1987-1995 | 1996 | 1997-2009 |

**Table 2 ICD codes for smoking-related diseases**

|  |  | ICD 8 | ICD 9  Basic tabulation | ICD 9  Detailed  tabulation | ICD 10  Detailed  tabulation |
| --- | --- | --- | --- | --- | --- |
| Lung cancer |  | A051 | B101 | 162 | C33-C34, |
| COPD |  | A093,  A096 | B323-B325 | 490-496 | J40—J47,  J67 |
| IHD |  | A083 | B27 | 410-414 | I20-I25 |
| Stroke |  | A085 | B29 | 430-438 | I60-I69 |
| Other smoking-related diseases | Neoplasms | A045-A047,  A050,  A055 | B08,  B090-B091,  B096,  B100,  B120,  B126 | 140-149,  150-151,  157,  161,  180,  188-189,  205 | C00-C16,  C25,  C32,  C53,  C64-C65,  C67,  C92 |
|  | Circulatory | A086 | B300-B302 | 440-448 | I70-I78 |
|  | Respiratory | A090-A092 | B321-B322 | 480-487 | J10-J18 |

In deriving the mortality data, certain specific points should be noted.

- The Czech Republic and Slovakia were parts of Czechoslovakia until 1992. For the period 1980-1991 mortality data for Slovakia were derived from data for Czechoslovakia based on the proportion of the population in Slovakia over this period. As WHO provide mortality data for the Czech Republic back to 1986, it was therefore only necessary to estimate this from Czechoslovakian data for earlier years. These calculations were carried out on a year, sex and age specific basis.
- Mortality data were unavailable from Lithuania for the years 1980 and 1983-1984. Data for 1981 were used for 1980. Data for 1983-1984 were derived by linear interpolation from the neighboring years (1982 and 1985).
- Mortality data for smoking-related diseases, other than the four main smoking-related diseases were not available from Lithuania for the years 1980 to 1992, the data provided by the Special List of causes as reported by some countries of the newly independent States of former USSR on the WHO homepage not being useful. Thus, mortality data from 1993 were applied for the previous years. However, the Special List of causes was used to derive the mortality estimates for lung cancer (B101), IHD (B27), COPD (B323-325) and stroke (B29).
- Mortality data were unavailable from Poland for the years 1997 and 1998 and were derived by linear interpolation from the data for 1996 and 1999.

**Reference**

1. Tachfouti N, Raherison C, Obtel M, Nejjari C. Mortality attributable to tobacco: review of different methods. Arch Public Health 2014,72(1):22.(Epub 20140701): doi:10.1186/2049-3258-72-22.
