# Supplementary material for: Using data on snus use in Sweden to compare different modelling approaches to estimate the population health impact of introducing a smoke-free tobacco product
Source: BMC Public Health. 2019 Oct 29;19:1411. doi: 10.1186/s12889-019-7714-0 (PMC6819486; doi:10.1186/s12889-019-7714-0)
Supplement: Supplementary file 2 — Additional file 2: Sources and methodology for estimating prevalence of tobacco use for Sweden. [file 12889_2019_7714_MOESM2_ESM.docx]

Title : “Using data on snus use in Sweden to validate a published modelling approach for estimating the population health impact of introducing a smoke-free tobacco product”

Authors : Smilja Djurdjevic, Laszlo Pecze, Rolf Weitkunat, Frank Luedicke, John Fry and Peter Lee

**SUPPLEMENTARY FILE 2**

Sources and methodology for estimating prevalence of tobacco use for Sweden

**Sources**

Three sources were used: International Smoking Statistics [1]; an internal report by Forey and Lee in 2011 which is attached as Appendix 1 to this Supplementary File; and Swedish national data obtained from the public health agency of Sweden - <http://fohm-app.folkhalsomyndigheten.se/Folkhalsodata/pxweb/sv/B_HLV/B_HLV__aLevvanor__aagLevvanortobak/hHLV_Tobaksvanor_alder.px/?rxid=aa498d10-f457-48a6-9fcd-a665bcd517aO>

**Inclusion criterion**

Surveys must relate to 1975 or later.

**Age groups – general considerations**

The objective was to derive annual estimates of prevalence of smoking in 20 standard age groups: 15, 16, 17, 18, 19, 20, 21-24, 25-29, 30-34 … 80-84 and 85+ years.

Data for the five tobacco use groups - Current Smoker, Former Smoker, Current Snus user, Former Snus user and Dual user (Current Smoker and Current Snus user) – are commonly published, not in five year age groups, but in broader age groups such as 20-39, 40-59, 60-79 and 80+ years. Studies only reporting results for one age group covering multiple standard age groups have been ignored, but studies where the original age group covers fully a single standard age group have been retained.

**Estimating prevalence data by age group using linear inter/extrapolation**

For each study matching the inclusion criteria, standard prevalence estimates were derived as described below for each age group, if not directly available from the source.

- Estimate the mean age for each original age group using the European standard population (ESP) [2] for 10-84 year-olds, given as 5 year groups, assuming a mean age of x + 0.5 years for x year-olds; and a mean age of 87.5 years for 85+ year-olds.
- Estimate the mean age for the 20 standard age groups using the same assumptions. These mean ages are 15.5, 16.5 … 20.5, 23, 27.5, 32.5 ... 82.5, and 87.5 years.
- For each standard age group, identify the two original age groups whose mean ages are nearest and next nearest to the mean age of the standard age group. If these two age differences are the same, choose the lower age group as being the nearest one and the higher as the next nearest.
- Use a linear regression based on the two selected age groups to estimate the prevalence for the standard age group.   Thus, if the mean ages of the original age groups are x_O1_, x_O2_, and the prevalences are y_O1_, y_O2_, the standardized prevalence y_S_ at the mean age of the standard age group x_S_ is estimated from the line (x_O1_, y_O1_) to (x_O2_, y_O2_). In the above, subscript 1 refers to the age group whose mean age is nearest to x_S_ and subscript 2 to the age group whose mean age is next nearest to x_S_.
- Retain the estimate if x_S_ = x_O1_ (i.e. the mean age of the original age group matches the mean age of the standard age group).
- Otherwise, only retain the estimate if the maximum of the two distances abs (x_O2_ – x_S_) and abs (x_O2_ – x_O1_) is less than a critical value. For instance, if x_S_ = 16.5, x_O1_ = 17 and x_O2_ = 15 the maximum distance (D) is the maximum of |15 -16.5| and |17 – 15| = 2, or if x_S_ = 23, x_O1_ = 25 and x_O2_ = 35 then D is the maximum of |35 – 23| and |35 – 25| = 12. The critical value depends on the distribution of the original age groups, as described below.

If none of the original age groups extends above 20 years, D must be <5.

If one of the original age groups extends over 20 years, the following critical values were applied:

- for the standard age groups 12, 13, 14 and 15 years, D must be <5.
- for the standard age groups 16, 17, 18 and 19 years, D must be <11.
- for the standard age groups 20, 21-24 and 25-29 years, D must be <20.
- for the standard age group 30-34 years and above, D must be <30.
- Negative extrapolated values were replaced by a zero value.
- Former smokers below the age of 18 were not considered. This allowed us to add an additional age point, 18, with a prevalence value of zero, so controlling the prevalence of former smokers at lower ages.
- One prevalence estimate of 41.8% for male former smokers in 1985 was removed from further calculations. This estimate, from source 50 (see Appendix 1 for sources), was in the valid age range but appeared to be an obvious outlier compared to estimates from other surveys for nearby year and age groups.

**Combining estimates from individual surveys**

For each age group a moving regression estimate was calculated from all the individual surveys using locally estimated scatterplot smoothing (LOESS).

- All surveys were given equal weight, their relative merits being ignored.
- For smoking, differences in product type (e.g. any smoking vs cigarette smoking) were not taken into account, as cigar and pipe smoking is relatively rare in Sweden [1].
- When a survey provided prevalence estimates for multiple different definitions of frequency of tobacco use (A = any, R = regular, U = unstated, * = other specified), a particular prevalence was selected according to the priority list R, U, A and *. For former smoking and dual use, each survey only provided prevalence estimates for a single definition of frequency, so this problem did not arise.
- If a LOESS estimate could not be derived for the beginning or the end of the time period, the closest values were considered.
- Negative LOESS values were replaced by a zero value.
- Finally, estimates for the age groups 10-14 and 15-19 years were created as the mean of the LOESS estimates for the five single year age groups. For the age group 20-24 years, the estimate was taken as one fifth of that for age 20 years plus four fifths of that for age 21-24 years.

R code was used for the generation of these estimates, and in particular the standard R loess function. This function is based upon a package developed by W. S. Cleveland, E. Grosse and W. M. Shyu  [3]

**Calculating prevalences for the nine subcategories for each age group**

The objective was to obtain percentage prevalences for the following 3 x 3 table:

|  | Cigarette smoking | | | | |
| --- | --- | --- | --- | --- | --- |
|  |  | Current | Former | Never | Total |
| Snus use - |  |  |  |  |  |
|  | Current | **a** | **b** | **c** | **A** |
|  | Former | **d** | **e** | **f** | **B** |
|  | Never | **g** | **h** | **i** | **C** |
|  | Total | **D** | **E** | **F** | **100** |

We start with the known values of **D** and **E** and calculate **F** = 100 – (**D**+**E**). We also know the percentage of current dual users **a.**

To complete the table, we use data from Table 4A of Appendix 1, in the following sequence:

1. Division 1: Divide the total % of current snus users who are noncurrent smokers, b + c (= A − a), into its separate parts.
2. Division 2: Divide the total % of never cigarette smokers who are noncurrent snus users, f + i (= F − c), into its separate parts.
3. Division 3: Divide the total % of former cigarette smokers who are noncurrent snus users, e + h (= E − b), into its separate parts.
4. Division 4: Divide the total % of current cigarette smokers who are noncurrent snus users, d +g (= D − a), into its separate parts.

In order to carry out these four divisions, the following process has been followed:

- Collect data from relevant studies for age groups with a range of up to 30 years.
- Calculate the means of these age groups using the European standard population (ESP1976) [2].
- Calculate the relevant division (1 to 4) for each mean age.
- Plot the division values along the line of ages.
- Assuming that below the age of 18 there are no former smokers or former snus users, add an additional point with 0% value for age 18 years.
- Fit a LOESS line. Derive a LOESS estimate at the mean ages of the standard age groups. If there is no estimate for older ages use the closest LOESS estimates.
- If f > E (as occurs in some younger age groups) replace E by the value of f. This is because we have more reliable data for A (Current Snus Users) than for E (Former Smokers).

In dividing the data as described, the following studies were found to be relevant. See Appendix 1 for the sources.

| Source | Former v never smokers among current snus users | Former v never snus users among never smokers | Former v never snus users among former smokers | Former v never snus users among current smokers |
| --- | --- | --- | --- | --- |
|  |  |  |  |  |
| ULF (3) | ✓ |  |  |  |
| SALT (4) | ✓ | ✓ | ✓ | ✓ |
| 2 AMI CC (6) | ✓ | ✓ | ✓ | ✓ |
| MONICA (7) | ✓ | ✓ | ✓ | ✓ |
| Malmö diet (9) | ✓ |  |  |  |
| IBD CC Stockholm  (11) | ✓ |  |  |  |
| VIP (15) | ✓ | ✓ | ✓ | ✓ |
| VIP (15) continued | ✓ | ✓ | ✓ | ✓ |
|  |  |  |  |  |
| NTS (22) | ✓ | ✓ | ✓ | ✓ |
| Your country and your life (23) | ✓ | ✓ | ✓ | ✓ |
| Stockholm Fire Brigade (25) | ✓ |  |  | ✓ |
| Stockholm Public Health follow-up (26) |  | ✓ |  |  |
| Skåne Public Health (28) | ✓ |  |  |  |
| Stockholm family diabetes (29) |  | ✓ |  |  |
| Vara municipality (32) | ✓ |  |  |  |
| AIR (33) | ✓ | ✓ | ✓ | ✓ |
| Stockholm County  60 year olds | ✓ | ✓ |  |  |

In these calculations all surveys were given equal weight, with their relative merits ignored. Where a study provided data for a broad age group and narrow age groups, only the broad age group was considered. It was assumed that the divisions do not change over time. If a study provides estimates for different years or year periods (for example: Study NTS, Years: 1985, 1986, 1987), the results for each period were considered separately. The Study MONICA gives sets of data for the year 1986 from two different sources. Both sources have been included and considered.

**References**

1. Forey B, Hamling J, Hamling J, Thornton A, Lee P. International Smoking Statistics. A collection of worldwide historical data. (Web edition). Sutton, Surrey: P N Lee Statistics and Computing Ltd; 2006-2016. Available: [www.pnlee.co.uk/iss.htm](file:///\\QNAP-1\tdrive\PMINTL\Project%2028\www.pnlee.co.uk\iss.htm).

2. Waterhouse J, Muir C, Correa P, Powell J. Cancer incidence in five continents. vol. III. Lyon, France: International Agency for Research on Cancer; 1976.

3. Cleveland WS, Grosse E, Shyu WM. Local regression models. In: Chambers JM, Hastie TJ, editors. Statistical Models in S. Wadsworth & Brooks/Cole; 1992.

**APPENDIX 1**

Gaining insight into the consequences of switching from

cigarettes to snus

Report 3

Characterizing dual use of snus and cigarettes, including comparison of cigarette consumption in dual users and in smokers of cigarettes only

Authors : B A Forey and P N Lee

Date : 19^th^ September 2011

EXECUTIVE SUMMARY

Literature searches were carried out to identify studies in Sweden that had published information relevant to characterization of the dual use of snus and cigarettes. Studies in populations likely to have smoking and snus use habits that were highly atypical of the general population (e.g. alcoholics) were excluded. 34 separate studies were identified. From each study data were extracted relating to the joint distribution of snus and smoking, the amount used by dual users, switching from smoking to snus, and the sequence of initiating smoking and snus use. For studies including adolescents and young adults, attention was restricted, if possible, to those aged 18+ years.

**Association between ever smoking and ever use**

19 studies provided relevant information. There was clear evidence that those who had ever smoked were more likely to have ever used snus. Based on n = 36 estimates, the random-effects estimate of the odds ratio (OR) was 3.13 (95% CI 2.28-4.30). The association was stronger in females (5.49, 4.14-7.30, n = 9) than in males (2.80, 1.94-4.06, n = 25). Within males, estimates were higher in later studies, in studies in the north of Sweden, and in studies where the definition of snus use was based on any frequency of use, rather than regular use. These factors did not fully explain the marked heterogeneity between the OR estimates.

The frequency of dual use varied from 3% to 44% with a mean of 18.2% (SE 1.7%). Dual use was less common in females (9.4%) than in males (22.0%) and, within males, values were higher for later studies, studies in the North and studies looking at any use rather than regular use.

**Association between current smoking and current use**

22 studies provided relevant information. In contrast to the evidence for ever smoking/snus use, there was no clear evidence that those who currently smoked were more likely to currently use snus. Here the random-effect estimate was 1.05 (0.95-1.16, n = 56) and there was no marked variation in the estimates by any of the cofactors studied. Though there was unexplained heterogeneity between estimates, this was much less than for ever smoking/snus use.

The frequency of dual use varied from 0.1% to 12.4% with a mean of 3.5% (SE 0.4%). Dual use is much less common in females (0.9%) than in males (4.8%), and, within males, values were higher for earlier studies and at younger ages.

The association for ever smoking/snus use, but not for current smoking/snus use can be explained by some people avoiding tobacco, many of the rest trying both products and ultimately settling for one.

**Tobacco consumption in single and dual users**

8 studies provided relevant information. Cigarette consumption in dual users is less than in smokers who do not use snus, with the ratio varying from 54% to 98% (mean 75%). Four of the studies allow comparison of snus use in dual users to that in snus users who do not smoke. Apart from a study of military conscripts, the results indicate a somewhat lower snus usage in the dual users. Comparison of total tobacco use in dual users and exclusive smokers or snus users is complicated by difficulties in assigning appropriate values to the weight of tobacco in a cigarette and in a can of snus.

The studies we considered provided limited, and conflicting, evidence based on levels of biomarkers of exposure, in dual and exclusive users, allowing no clear conclusion.

**Switching ratios**

Based on more detailed breakdowns of the joint distribution of smoking and snus use, it was possible to derive estimates from 19 studies of the ratio of the number of people switching from smoking to snus, relative to those continuing to smoke. Interpretation of the data is complicated by the differing definitions available from the different studies. However, it is clear that the ratio is much lower for women than for men. Within men, the ratio generally decreases with age and increases over time. For studies conducted before the year 2000, non-switchers generally outnumber switchers, while for later studies switchers are in the majority, with switching ratios exceeding 2 from recent studies of younger men.

**Sequence of initiation**

Only 3 studies provided information. All these studies showed that a large majority of male dual users reported having smoked first. Generally this was about 70-80%, except for the older age group in an early study where virtually all (99%) had smoked first. Note that studies in adolescents would provide additional data here.

INDEX

Text Page

[1. Introduction 9](#_Toc304193936)

[2. Methods 10](#_Toc304193937)

[2.1 Literature searches 10](#_Toc304193938)

[2.2 Study inclusion criteria 10](#_Toc304193939)

[2.3 Structure of the report 11](#_Toc304193940)

[2.4 Joint distribution of snus and smoking 11](#_Toc304193941)

[2.5 Amount used by dual users 12](#_Toc304193942)

[2.6 Switching from smoking to snus 13](#_Toc304193943)

[2.7 Sequence of initiating smoking and snus use 15](#_Toc304193944)

[2.8 Meta-analysis 15](#_Toc304193945)

[2.9 Checking 17](#_Toc304193946)

[3. Results 18](#_Toc304193947)

[3.1 Searches 18](#_Toc304193948)

[3.2 The individual studies 18](#_Toc304193949)

[3.2.1 The Swedish Construction Workers Study 18](#_Toc304193950)

[3.2.2 The Kalixanda study 19](#_Toc304193951)

[3.2.3 Swedish survey of living conditions- ULF 20](#_Toc304193952)

[3.2.4 Swedish Twin Registry – SALT 23](#_Toc304193953)

[3.2.5 Swedish case-control study of multiple sclerosis 25](#_Toc304193954)

[3.2.6 Two AMI case control studies in Stockholm and Västernorrland 26](#_Toc304193955)

[3.2.7 MONICA – cross-sectional studies in Northern Sweden 27](#_Toc304193956)

[3.2.8 MONICA – follow-up study in Northern Sweden 29](#_Toc304193957)

[3.2.9 Malmö Diet and Cancer Study 31](#_Toc304193958)

[3.2.10 Swedish Annual Level-of-Living Survey 32](#_Toc304193959)

[3.2.11 Case-control study of IBD in Stockholm 33](#_Toc304193960)

[3.2.12 BROMS study in Swedish children 34](#_Toc304193961)

[3.2.13 Uppsala County Study 35](#_Toc304193962)

[3.2.14 Case-control study of oral cancer in four northern counties 36](#_Toc304193963)

[3.2.15 Västerbotten Intervention Program (VIP) 37](#_Toc304193964)

[3.2.16 Swedish Medical Birth Register 39](#_Toc304193965)

[3.2.17 Case-control study of gastric cancer 40](#_Toc304193966)

[3.2.18 Eurobarometer 41](#_Toc304193967)

[3.2.19 Swedish Twin Registry - STAGE 42](#_Toc304193968)

[3.2.20 Telephone survey of smokers 43](#_Toc304193969)

[3.2.21 Surveys of 21-year olds in northern town 44](#_Toc304193970)

[3.2.22 NTS surveys 45](#_Toc304193971)

[3.2.23 Your country and your life survey 50](#_Toc304193972)

[3.2.24 Health on equal terms 51](#_Toc304193973)

[3.2.25 Stockholm City Fire Brigade 52](#_Toc304193974)

[3.2.26 Stockholm Public Health survey 53](#_Toc304193975)

[3.2.27 Göteborg Public Dental Service study 55](#_Toc304193976)

[3.2.28 Skåne public health survey 56](#_Toc304193977)

[3.2.29 Stockholm family history of diabetes study 57](#_Toc304193978)

[3.2.30 Värmland ice hockey players 58](#_Toc304193979)

[3.2.31 Älvsborg county study 59](#_Toc304193980)

[3.2.32 Vara municipality study 60](#_Toc304193981)

[3.2.33 Atherosclerosis and Insulin Resistance Study (AIR) 61](#_Toc304193982)

[3.2.34 Stockholm 60-year olds study 63](#_Toc304193983)

[3.2.35 Säve military conscripts 64](#_Toc304193984)

[3.2.36 Stockholm county 31-40 year olds 66](#_Toc304193985)

[3.3 Overall analysis 67](#_Toc304193986)

[3.3.1 Association between ever smoking and ever snus use 67](#_Toc304193987)

[3.3.2 Association between current smoking and current snus use 69](#_Toc304193988)

[3.3.3 Tobacco consumption in single and dual users 70](#_Toc304193989)

[3.3.4 More detailed joint distribution of smoking and snus 72](#_Toc304193990)

[3.3.5 Switching ratios 72](#_Toc304193991)

[3.3.6 Sequence of initiation 73](#_Toc304193992)

[4. Summary 74](#_Toc304193993)

[Tables 77](#_Toc304193994)

[TABLE 1 Association of ever (vs never) snus use with ever (vs never) smoking 77](#_Toc304193995)

[TABLE 2 Association of current (vs non-current) snus use with current (vs non-current) smoking 85](#_Toc304193996)

[TABLE 3 Daily consumption^a^ of tobacco in single and dual users 98](#_Toc304193997)

[TABLE 4 Association of current/ex/never snus use with current/ex/never smoking – studies giving at least 6 categories 101](#_Toc304193998)

[TABLE 4A Numbers of subjects 101](#_Toc304193999)

[TABLE 4B Percentages 110](#_Toc304194000)

[TABLE 5 Ratios of switchers to non-switchers 120](#_Toc304194001)

[TABLE 6 Detailed distribution of switching patterns – ULF study (section 3.2.3) 133](#_Toc304194002)

[TABLE 7 Detailed distribution of switching patterns – SALT study (section 3.2.4) 135](#_Toc304194003)

[TABLE 8 Detailed distribution of switching patterns – MONICA follow-up study (section 3.2.8) 137](#_Toc304194004)

[TABLE 9 Detailed distribution of switching patterns – VIP follow-up study (section 3.2.15) 141](#_Toc304194005)

[TABLE 10 Meta-analysis of ORs for ever snus use and ever smoking 142](#_Toc304194006)

[TABLE 11 Percentages of dual users for ever snus use and ever smoking 145](#_Toc304194007)

[TABLE 12 Meta-analysis of ORs for current snus use and current smoking 147](#_Toc304194008)

[TABLE 13 Percentages of dual users for current snus use and current smoking 150](#_Toc304194009)

[References 152](#_Toc304194010)

1. Introduction

The work to be carried out in this project involves three parts. Part I concerns comparison of the health risks of dual users of cigarettes and snus, and of switchers from cigarettes to snus with the health risks of continuing cigarette smokers. Part II concerns changes in health risks associated with cutting down smoking. Part III concerns characterization of the dual use of snus and cigarettes, including comparison of cigarette consumption in dual users and in smokers of cigarettes only.

This report, report 3, concerns evidence relevant to Part III. Some of this evidence has already been presented in report 1, which concerned studies of health risks of snus conducted in Sweden (or occasionally in neighbouring Scandinavian countries).

# 2. Methods

## 2.1 Literature searches

All papers previously included in PNL’s published review of the epidemiological evidence relating snus to health [1], in Report 1 of the present project [2] and in the Sweden chapter of International Smoking Statistics [3] were considered. The extensive collection of papers at P N Lee Statistics and Computing was also searched. A Medline search using the search term

(“Smoking”[Mesh] OR “Smoking Cessation”[Mesh]) AND “Tobacco, Smokeless”[Mesh] AND (“Sweden”[Mesh] OR “Finland”[Mesh] OR “Norway”[Mesh])

was carried out

## 2.2 Study inclusion criteria

The aim was to include studies where the subjects were, in broad terms, likely to be typical of the general population in their smoking and snus use habits. Thus studies were excluded if:

- subjects were likely to have highly unusual smoking or snus use habits (e.g. alcohol-dependant subjects [4])
- subjects were all cases with some medical condition (e.g. diabetics [5], Raynaud’s phenomena [6])
- subjects were volunteers for an experimental or clinical study with <100 subjects.

However studies of occupational groups were included (even though workers in some occupations may use snus because of restrictions, dangers or impracticalities of smoking while at work). For case-control studies, only the controls are considered.

Studies where subjects were selected according to their smoking or snus habits were included only where that selection did not make their results inappropriate.

Further, attention in this report is restricted to studies in Sweden, and to studies of adults. For studies including adolescents and young adults, attention is restricted, if possible, to those aged 18+.

Multiple papers referring to the same studies were identified where possible, and any overlapping studies are mentioned (i.e. where the same subjects have participated in more than one study). However as many nationwide studies have been conducted, and as certain counties, particularly in Northern Sweden, have been selected for more than one regionally-based study, it is impossible to rule out the possibility that some individuals may have participated in more than one survey.

## 2.3 Structure of the report

From each study, relevant data were extracted, where available, relating to the joint distribution of snus and smoking, the amount used by dual users, switching from smoking to snus, and the sequence of initiating smoking and snus use. Section 3 considers each study in turn, briefly describing the study and presenting the relevant data, except where previously given in Report 1 [2]. The overall results are summarized in Tables 1 to 9, and discussed in section 4. The rest of the methods section gives further detail on the data extracted and the structure of the tables.

## 2.4 Joint distribution of snus and smoking

Methods for assessing the joint distribution of tobacco use are as described in §2.2 of Report 1 [2].

Tables 1 and 2 are equivalent to Tables 1 and 2 in Report 1 [2], and give the odds ratios for ever and current use respectively. Table 4 gives summary information on the available 3×3 tables of joint use from cross-sectional studies (current/ex/never), including all those studies that provide at least 6 of the 9 categories. This table is subdivided into two parts – the first giving numbers of subjects surveyed and the second percentages. Results are described fully in the study-specific sections of text, except where previously described in Report 1 [2], in which case they are shown only in the summary tables.

Results are presented, if available, by sex and year/period. Results for the sexes combined are given only when sex-specific results are not available. Results for females are given only for those studies with more than 10 dual users. Age-specific results are also given for men. Results stratified by other demographic or life-style factors are mentioned but not shown.

Where there is a choice of definition, regular and occasional smokers (or snus users) have generally been combined.

The definitions of smokers are shown in the summary tables by a 2-part code. The first part indicates the smoking product referred to : C=cigarettes, A=any product (i.e. cigarettes, pipe and/or cigars), U=unspecified (i.e. either the original questionnaire did not specify, simply asking “do you smoke”, or the paper reporting the results did not specify). The second part indicates the frequency of smoking: R=regular or daily; W=at least weekly; A=any (i.e. regular or occasional/non-daily); U=unspecified. If the report did not specify either the product or the frequency, but categorized the smokers by cigarettes per day, then the codes C and R are assumed.

Arrows and heavy table borders are used to indicate where table cells have been combined.

## 2.5 Amount used by dual users

Results are reported comparing the amount smoked by exclusive smokers and by dual users, and similarly comparing the amount of snus used by exclusive users and by dual users. If available, the means of the current amount smoked/used are presented, otherwise the distribution by amount is shown. However distributions by regular vs intermittent or non-daily use have not been included.

Lifetime consumption (e.g. pack-years) is included where no other measure is available. However interpretation is very limited, as dual users may have very different durations of use for the two products.

Results for biochemical measures such as cotinine are also reported, where available.

As for joint use, results are described in full in the text unless previously given in Report 1 [2]. Results are summarised in Table 3, equivalent to Table 3 of Report 1.

## 2.6 Switching from smoking to snus

Ideally, one might wish to identify persons who switch from smoking to snus as those who smoke exclusively for a significant period, then possibly have a short period of dual use, and finally settle to exclusive snus use for a significant period. However no study that we are aware of enables “switchers” to be identified as clearly as this. Consequently we consider a number of different definitions, and term these *S1, S2* etc in this report.

Similarly, “non-switchers” would ideally be identified as those current smokers who have smoked continuously and exclusively, but we consider a number of different definitions and term these *N1, N2* etc.

For a cross-sectional study, where the 3×3 table for the joint distribution is available as follows:

|  |  | Snus use |  |  |
| --- | --- | --- | --- | --- |
|  |  | Current | Former | Never |
| Smoking^a^ | Current | A | B | C |
|  | Former | D | E | F |
|  | Never | G | H | I |

“switchers” can be defined as cell D. We term this definition of switchers as *S1*. Note however that these subjects may have started snus use before or during the period when they smoked, or they may have had a period of using neither product between quitting smoking and starting snus. Switching may have occurred at any time in the past.

“Non-switchers” can be defined as cell C (termed *N1*), which meets the ideal definition, except that they may have had periods of quitting in the past. From studies with less detail available, B+C (i.e. subjects may have used snus at some time in the past) would be an acceptable alternative (*N2*).

A cross-sectional study may have asked dual users the order of first use. This would allow a subset of cell D – those who first smoked before they first used snus – to be identified as switchers (*S2*). Even if only a 2×3 table is available:

|  |  | Snus use |  |
| --- | --- | --- | --- |
|  |  | Ever | Never |
| Smoking^a^ | Current | A+B | C |
|  | Former | D+E | F |
|  | Never | G+H | I |

then switchers (*S3)* can be defined as the subset of D+E who started smoking first, although this is a less satisfactory definition as these “switchers” may in fact have given up snus as well as quitting smoking.

From longitudinal studies, it may be possible to assess switching between different phases of the study. Various definitions may be possible depending on the categories available at baseline and follow-up. The following are defined:

|  | Baseline | Follow-up |
| --- | --- | --- |
| *S4* | Current smoke only | Current snus only |
| *S5* | Current smoke only or current dual user | Current snus only |
| *S6* | Current smoke only and never snus | Current snus only |
|  |  |  |
| *N3* | Current smoke only | Current smoke only |
| *N4* | Current smoke only or current dual user | Current smoke only |
| *N5* | Current smoke only or current dual user | Current smoke only or current dual user |
| *N6* | Current smoke only and never snus | Current smoke only |

Note that, apart from definitions S6 and N6, snus may have been used before baseline, and also that for all definitions there may have been a period of quitting, or of dual use, between the two time points examined.

Clearly the length of time between baseline and follow-up will also affect the likelihood of subjects having switched.

Results are given in the text and in summary Table 5, and are expressed where possible as the ratio of numbers of switchers to number of non-switchers.

## 2.7 Sequence of initiating smoking and snus use

Any results referring to whether dual users started by smoking first, or by snus first (or rarely, by starting both habits at the same time) are reported in the text.

Note that many of the studies giving results on switching or on the sequence of initiation also provide information on cessation or initiation. These aspects were considered in PNL’s review [1], but are not considered in this report.

## 2.8 Meta-analysis

Fixed-effect and random-effects meta-analysis were conducted using the methods of Fleiss and Gross [7], with heterogeneity quantified by the ratio of the heterogeneity chisquared to its degrees of freedom. For various factors, the meta-analysis was repeated at each level of the factor, and differences in the fixed-effect estimates by level of the factor were tested using an F-test which compared variation between and within levels of the factors considered.

From studies providing multiple ORs, inclusion in the meta-analysis was decided on the following basis, to avoid, as far as reasonable, “double-counting” any subjects who contributed to more than one OR.

**Sex** Since sex-specific results have been selected if available, and sexes-combined results only if sex-specific results are not available (§2.4), there is no requirement for any further restriction on the ORs that can enter a meta-analysis.

**Age** As the ORs from non-overlapping age-groups are independent, more than one such OR can validly enter a meta-analysis. Where both overall (all ages) and age-specific ORs were available from a study, the main analyses were conducted including the overall OR. However where a study did not provide an overall estimate (e.g. the VIP study which reported only non-contiguous age groups, see §3.2.15), the age-specific ORs were used in the main analysis.

When age was tested as a factor, age-specific ORs were used if available. Different studies report results using different schemes to define their age groups, (e.g. 30-39 and 40-49 in one study, but 20-34, 35-49 in another). In defining the levels of the age factor, the objective was to include ORs referring to age-groups wholly within some reasonably narrow age range, but to avoid excluding ORs from too many studies. The age ranges were chosen as ≤45, 35-64 and ≥55. These overlapping ranges allow, for instance, ORs for all the example age groups just mentioned to be entered into the meta-analysis. However if a narrow age group does fall into two of the age ranges, it is allocated only to the more extreme range (e.g. 40 year olds from the VIP study are allocated to the lowest range, while 58 year olds from the AIR study are allocated to the highest range). For studies not providing age-specific results, the overall OR was also included if the whole study age range fell wholly within one of the age ranges.

**Period** Studies were divided into three periods, before 1990, 1990-1999 and 2000 onwards. Studies that referred to more than one of these periods was allocated to the period into which most years fell provided not more than two years fell into another period. ORs from separate waves of cross-sectional studies are independent if each wave draws a new sample, and hence more than one such OR can enter a meta-analysis. However ORs from longitudinal studies where the same subjects participate on more than one occasion may not be independent, and care was taken to exclude ORs which would have double counted such subjects; the latest available OR was included.

**Other factors** considered were:

- region, categorised as (1) nationwide or multi-region, (2) north and (3) south or central.
- regularity of snus use, categorised as (1) regular or daily and (2) weekly, any or unspecified (where unspecified included both that the reporting paper did not give any definition, and where the original questionnaire left the definition open to the respondent e.g. by simply asking “*do you use snus?*”)
- regularity of smoking, categorised as for regularity of snus use
- smoking product, categorised as (1) cigarettes and (2) any (i.e. cigarettes, pipe and/or cigars), or unspecified.

## 2.9 Checking

All data extracted, calculations made and overall analyses were independently checked.

# 3. Results

## 3.1 Searches

The Medline search was carried out on 14^th^ July 2011, scoring 148 hits. 12 of these which were not already available, and which were selected as potentially relevant on the basis of their abstract/title, were obtained (except for one [8] which is temporarily unavailable via the British Library). A total of 276 papers were examined, of which 61 were found to have relevant results referring to 34 separate studies.

Studies are considered in turn in the following sections, starting with those already described in sections 3.2.1 – 3.2.17 of Report 1 [2]. For convenience, the section numbers refer to the same studies as before (except for the studies within the MONICA framework where two original case-control studies are replaced by baseline (3.2.7) and follow-up (3.2.8) studies; note that no data are presented in sections 3.2.10 and 3.2.12.) Other studies cited in PNL’s snus review [1] are then considered in sections 3.2.18 – 3.2.24, with some additional studies considered in sections 3.2.25-3.2.36. Where results have been presented previously, they are reproduced only in the summary Tables, but any new results are fully described in the text sections that follow.

## 3.2 The individual studies

### 3.2.1 The Swedish Construction Workers Study

This study was described in §3.2.1 of Report 1 [2]. Results for the joint distribution of tobacco use and for the amounts smoked/used are reproduced in Tables 1 and 3 respectively, together with further age-specific results. The results are based on three papers. Two (Norden et al [9] and Zendehdel et al [10]) are based on the same members of the cohort but report different age groups – that from Zendehel being shown in Table 1. The third paper, by Carlens et al [11], omits cohort members recruited before 1978, as the validity of the snus data gathered in that period has been questioned [10].

### 3.2.2 The Kalixanda study

This study [12], conducted in two communities in Northern Sweden, was described in §3.2.2 of Report 1 [2], and results are included in Tables 1, 2, 3 and 4, including age-specific results not previously included. As previously noted, the study reported no current users of one product who were former users of the other product, casting doubt on the validity of this categorization.

### 3.2.3 Swedish survey of living conditions- ULF

A nationwide survey entitled Undersknigar on levnadsförhållanden (ULF, known in English as the Survey of Living Conditions, or Level of Living) has been conducted annually by Statistisk Centralbyrån (SCB, Statistics Sweden) since 1977, and includes questions on smoking. A supplement which changes in 8-year waves and stays for 2 consecutive years includes questions on snus [13]. Only current snus use was recorded. In 1980-81 it was categorized only as yes or no, and was asked only of men; in the later surveys, it was categorized as daily, occasional or none. Smoking was defined as daily smoking (current, ex or never).

Two papers reporting follow-up studies, both based on the 1988-89 wave but using different age groups, were described in §3.2.3 [14] and §3.2.10 [15] of Report 1 [2].

In PNL’s snus review [1], estimates based on data provided by Högstorp [16] were reported, and are reproduced here in Table 2, except that

- an error has recently been discovered in the calculations for 1996-97, and the corrected estimates are shown here, and
- a slightly different method for the all-ages estimates affects the female results

Note that all the estimates are approximate and may be subject to rounding error. More details are shown in Tables 4 and 5. These data refer to four waves of the survey (the first three being pooled over two consecutive years, with only one year available for the last wave). Results for women are shown only for all ages. Daily and occasional snus use have been combined in Tables 2, 4 and 5.

For the 2010 survey, results are available on the SCB website [17], for daily smoking, daily snus use, and daily smoking and/or snus, from which the joint distribution can be derived (Table 2). These are available only as percentages, so the confidence interval (CI) of the odds ratios cannot be estimated. Results are also available (not shown) by household type, foreign/Swedish origin, education, SES and municipality.

Further data on regular/occasional snus use (by daily smoking) are also available [16] but are not shown here.

Tillgren et al [18] reported on a follow-up study in which a random sample of about 40% of participants from the 1980-81 wave were reinterviewed at the same time as the 1988-89 wave. As the 1980-81 survey did not ask women about snus use, changes in both smoking and snus habits can only be studied among the 2383 men in the follow-up panel. Stenbeck et al [[13] and personal communication] reported on a similar panel follow-up in 1996-97 from those first interviewed in 1988-89, with results available only for the 2156 men. Full details are given in Table 6. The relative frequency of switching among those who initially smoked is shown below and in Table 5:

| Baseline | | |  | Follow-up | | |  | Ratio  (Switchers: non-switchers) | | |
| --- | --- | --- | --- | --- | --- | --- | --- | --- | --- | --- |
| Year | Age | Smoking/snus |  | Switched to snus only | Continued smoking | |  | S4:N3 | S5:N4 | S5:N5 |
|  |  |  |  |  | Smoke only | Smoke only or dual user |  |  |  |  |
| 1980-81 | 16-84 | Smoke only |  | 32 | 450 | 463 |  | 0.07 |  |  |
|  |  | Smoke only or dual user |  | 69 | 480 | 530 |  |  | 0.14 | 0.13 |
|  |  |  |  |  |  |  |  |  |  |  |
| 1988-89 | 16-44 | Smoke only |  | 25 | 110 | 120 |  | 0.23 |  |  |
|  |  | Smoke only or dual user |  | 43 | 115 | 137 |  |  | 0.37 | 0.31 |
|  |  |  |  |  |  |  |  |  |  |  |
|  | 45-84 | Smoke only |  | 19 | 180 | 186 |  | 0.11 |  |  |
|  |  | Smoke only or dual user |  | 31 | 188 | 207 |  |  | 0.16 | 0.15 |
|  |  |  |  |  |  |  |  |  |  |  |
|  | 16-84 | Smoke only |  | 44 | 290 | 306 |  | 0.15 |  |  |
|  |  | Smoke only or dual user |  | 74 | 303 | 344 |  |  | 0.24 | 0.22 |

Tillgren et al [18] presented both univariate and multivariate analyses of the risk of remaining a daily smoker, using a wide range of factors but unfortunately not snus!

### 3.2.4 Swedish Twin Registry – SALT

The Screening Across the Lifespan Twin (SALT) study of twins born before 1959 was described in §3.2.4 of Report 1 [2], and results based on 16642 males with no previous CVD at baseline as reported by Hansson et al [19] are included in Tables 1-5.

The results from Hansson et al [19] are shown in Table 1 in preference to those reported earlier by Furberg et al [20] and included in PNL’s snus review [1], being based on a slightly larger cohort and having “current” results also available (Table 2); it is unclear why the Furberg cohort is smaller (14932 males), as there is no mention of restriction on previous CVD or for any other reason. Excluding those with incomplete information or who only “smoked at parties” (4%, for whom the age of starting tobacco use was unknown), Furberg et al [20] presented results for 13822 men on the joint distribution of cigarette smoking (current/former × regular/occasional) and ever snus (regular/ occasional), with dual users further categorized by the order of starting (snus first, cigarettes first or same time) (Table 7). Among those who ever smoked or used snus, the distribution including the order of starting for dual users can be summarized as follows:

| Definition of smoking and snus use | Ever used snus, never smoked | Ever smoked, never snus | Ever smoked and used snus |  |  |
| --- | --- | --- | --- | --- | --- |
|  |  |  | Snus first | Smoked first | Same time |
| Regular | 976 | 5370 | 208 | 2025 | 277 |
|  | (11.0%) | (60.6%) | (2.3%) | (22.9%) | (3.1%) |
|  |  |  |  |  |  |
| Regular or occasional | 1036 | 5466 | 291 | 2422 | 370 |
|  | (10.8%) | (57.0%) | (3.0%) | (25.3%) | (3.9%) |

This categorization also allows “switchers” to be defined as former smokers who ever used snus and who started using smoking before snus use (but note that this also includes those who quit both smoking and snus use). Thus we have:

| Definition of smoking and snus use | Current smoker, never snus | Switchers | Ratio S3:N1 |
| --- | --- | --- | --- |
| Regular smoking, regular snus | 2120 | 1701 | 0.80 |
| Regular smoking, any snus | 2120 | 1838 | 0.87 |
| Any smoking, any snus | 2278 | 1928 | 0.85 |

Another analysis of the data by Furberg et al [21], based on a total of 14424 men, gave similar results (not shown) for the order of starting among dual users. It also allowed switchers to be defined as former smokers who currently use snus (regardless of which product was first). This gave:

| Definition of smoking and snus use | Current smoker, never snus | Switchers | Ratio S1:N1 |
| --- | --- | --- | --- |
| Any smoking, any snus | 2416 | 1470 | 0.61 |

### 3.2.5 Swedish case-control study of multiple sclerosis

This nationwide study [22] was described in §3.2.5 of Report 1 [2], and results are included in Table 1 (footnote). Although no results are available for the amount of snus currently used, the distribution of the controls according to lifetime (cumulative) snus use are as follows:

| Packet-years^a^ | Snus only^b^ | Smoking and snus^b^ |
| --- | --- | --- |
| <5 | 26 (41%) | 102 (55%) |
| 5+ | 38 (59%) | 84 (45%) |

^a^ A “packet year” is defined as smoking 1 packet of snus daily for a year.

^b^ Ever snus, and ever smoked, assessed in “index year”, i.e. year of disease onset for the matched case, mean 3.8 years before inclusion in study.

### 3.2.6 Two AMI case control studies in Stockholm and Västernorrland

This pooled analysis [23] of two comparable studies was described in §3.2.6 of Report 1 [2], and results are included in Tables 1-5. Note that former smokers had stopped smoking 1 year before inclusion in the study, while former snus users had stopped use 2 years before.

### 3.2.7 MONICA – cross-sectional studies in Northern Sweden

A series of five cross-sectional studies were conducted as part of the northern Sweden component of the WHO Multinational Monitoring of Trends and Determinants in Cardiovascular Diseases (MONICA) study. Case-control studies [24,25] using participants from the cross-sectional studies as controls were described in §3.2.7 and §3.2.8 of Report 1 [2]. A follow-up study is considered in the next section of this report.

The cross-sectional studies were conducted in 1986, 1990, 1994, 1999 and 2004 in Norrbotten and Västerbotten counties. The age range was 25-64 in the first two surveys and 25-74 thereafter. Both men and women were surveyed, but some reports refer only to the men.

Some papers reported the joint distribution of tobacco use directly as a 3×3 table ([26] for 1986, [27,28] for pooled years), while another paper [29] presented information from which the 3×3 table could be estimated for the years 1986, 1990, 1994 and 1999. A further paper [30] gave only the 2×2 table for current use in 2004, but additionally gave age-specific results. However the definitions used in these papers are not exactly comparable, and there are some unexplained discrepancies. Results, including those previously shown in PNL’s snus review [1], are shown in Tables 1, 2, 4 and 5. Numbers of subjects have generally been estimated from percentages, in some cases read from bar charts, so may be subject to rounding error.

For the first four surveys combined (1986-1999), Rodu et al [29] reported the following mean daily consumption figures for men (also shown in Table 3):

| Smoking: | current | current | current | current | ex | never | non^a^ |
| --- | --- | --- | --- | --- | --- | --- | --- |
| Snus: | never | ex | non^a^ | current | current | current | Current |
| Cigarettes per day | 16.0 ± 7.98 | 15.1 ± 7.52 | 15.8 ± 7.89 | 10.8 ± 6.16 |  |  |  |
| Snus packages per day |  |  |  | 0.25 ± 0.20 | 0.41 ± 0.25 | 0.44 ± 0.27 | 0.42 ± 0.26 |

Mean ±SD

^a^ Estimated by combining means for never/ex

From the 1990 survey, Eliasson et al [31], after excluding 38 subjects who smoked a pipe or cigars, reported the following mean consumption and biomarker levels for current smokers/users among the men:

|  | Snus only | Smoke only | Snus and smoking |
| --- | --- | --- | --- |
| Cigarettes per day | – | 16.5 (15.3-17.8) | 10.1 (8.0-12.2) |
| Snus cans per week | 3.2 (2.9-3.5) | ­– | 2.5 (2.2-2.9) |
| Tobacco grams per day^a^ | 22.9 (20.7-25.0) | 16.5 (15.3-17.8) | 28.2 (25.0-31.4) |
| Plasma cotinine (ng/ml)^b^ | 351 (277-425) | 242 (209-275) | 308 (242-373) |
| Plasma nicotine (ng/nl)^b^ | 15.5 (9.6-21.4) | 9.8 (7.6-12.1) | 9.5 (5.0-14.1) |

Mean (95%CI)

^a^ Assuming 1 cigarette = 1 g and 1 can of snus = 50 g

^b^ Available for 25% of whole sample

Results for the women snus users (12 exclusive and 4 dual users) were not reported.

### 3.2.8 MONICA – follow-up study in Northern Sweden

Rodu et al [32] reported on a follow-up study in 1999 to which all participants of the 1984, 1990 and 1994 cross-sectional surveys (see §3.2.7) were invited. About 70% were successfully followed. The results refer to those aged 25-64 at baseline.

Extensive tables of the joint distribution of tobacco were presented for men, allowing cross-tabulations of 9 baseline categories (i.e. the usual 3×3 current/ex/never table) by 4 categories at follow-up (2×2 current/non) by 3 baseline years (the only exception being that the continuing never smokers/never users were not given in full detail). These are shown in full as Table 8. Among the **baseline current smokers who had never used snus**, switchers can be defined as those who at follow-up used snus only, and compared with those who at follow-up smoked only (S6:N6). The numbers and ratio were as follows:

|  | Baseline year | Follow-up period (years) | | Switchers | Continuing smokers | Ratio S6:N6 |
| --- | --- | --- | --- | --- | --- | --- |
|  | 1986 | 13 |  | 7 | 34 | 0.21 |
|  | 1990 | 9 |  | 5 | 37 | 0.14 |
|  | 1994 | 5 |  | 3 | 42 | 0.07 |

For comparability with other studies, the alternative definitions among those **currently exclusively smoking at baseline** (but irrespective of past snus use) can also be given:

|  | Baseline year | Follow-up period (years) | | Switchers | Continuing smokers | Ratio S4:N3 |
| --- | --- | --- | --- | --- | --- | --- |
|  | 1986 | 13 |  | 11 | 42 | 0.26 |
|  | 1990 | 9 |  | 13 | 42 | 0.31 |
|  | 1994 | 5 |  | 5 | 46 | 0.11 |

Less detailed tables were presented for women (Table 6). Using alternative definitions by including **all baseline current smokers** (irrespective of concurrent or past snus use), the following estimates can be obtained for both men and women:

| Sex | Baseline year | Follow-up period (years) | | Switched to snus only | Continued smoking | |  | Ratio |  |
| --- | --- | --- | --- | --- | --- | --- | --- | --- | --- |
|  |  |  |  |  | Smoke only | Smoke only or dual use |  | S5:N4 | S5:N5 |
| Men | 1986 | 13 |  | 25 | 44 | 55 |  | 0.57 | 0.45 |
|  | 1990 | 9 |  | 24 | 43 | 63 |  | 0.56 | 0.38 |
|  | 1994 | 5 |  | 9 | 47 | 59 |  | 0.19 | 0.15 |
|  |  |  |  |  |  |  |  |  |  |
| Women | 1986 | 13 |  | 5 | 90 | 92 |  | 0.06 | 0.05 |
|  | 1990 | 9 |  | 3 | 86 | 87 |  | 0.03 | 0.03 |
|  | 1994 | 5 |  | 6 | 113 | 114 |  | 0.05 | 0.05 |

These results are also shown in Table 5.

### 3.2.9 Malmö Diet and Cancer Study

This study [33] was described in §3.2.9 of Report 1 [2], and results are included in Tables 2-5. Note that current smokers include both regular and occasional smokers, with daily cigarette consumption stated in grams, while snus users were categorized according to packages of snus used per week.

### 3.2.10 Swedish Annual Level-of-Living Survey

This study, previously reported in §3.2.10 of Report 1 [2], is the same as the study reported in §3.2.3 above.

### 3.2.11 Case-control study of IBD in Stockholm

This study [34] was described in §3.2.11 of Report 1 [2], and results are included in Tables 1, 4 and 5.

### 3.2.12 BROMS study in Swedish children

The BROMS (Children’s Smoking and Environment in Stockholm County) study was described in §3.2.12 of Report 1 [2], and in PNL’s snus review [1] but is not considered further here as it involves only adolescent subjects.

### 3.2.13 Uppsala County Study

This study [35] was described in §3.2.13 of Report 1 [2], and results for males are included in Table 1. Axéll and Liedholm [36] also presented results from this study for the sexes combined, but their categorization of “mixed tobacco users” included dual pipe and cigarette smokers, so does not provide relevant information.

### 3.2.14 Case-control study of oral cancer in four northern counties

This study [37], conducted in Norbotten, Västerbotten, Jämtland and Västernorrland, was described in §3.2.14 of Report 1 [2], and results are included in Tables 1, 2, 4 and 5. Results are for the sexes combined, although only 1 woman reported snus use, and it was not stated whether she was a case or a control subject. Although no results are available for the amount of smoking tobacco or snus currently used, the distribution of subjects according to lifetime (cumulative) use are given, based on 277 pairs (81%) with complete data:

|  |  | Snus use |  |  |
| --- | --- | --- | --- | --- |
|  |  | Never | Low | High |
| Smoking | Never | 133  (46.3%) | 8  (2.8%) | 12  (4.2%) |
|  | Low | 52  (18.1%) | 7  (2.4%) | 7  (2.4%) |
|  | High | 58  (20.2%) | 8  (2.8%) | 2  (0.7%) |

Snus use: assuming 1 quid of snus = 1 g.

Smoking: assuming 1 cigarette = 1 g, 1 cheroot = 3 g, 1 cigar = 5 g, 1 pack of pipe tobacco = 50 g.

Low and high were defined as ≤ and > respectively median value among the controls, the medians being 156.0 kg for snus and 124.8 kg for smoking.

The OR is 0.25 (0.04-1.62) for high vs low usage among dual users, although this is based on very small numbers.

### 3.2.15 Västerbotten Intervention Program (VIP)

Since 1985, all inhabitants of Västerbotten county have been invited to a health examination in the year they turn 40, 50 or 60 years of age. Up to 1995, the study also included 30-year olds. This study contributed the majority of the controls for a nested case-control study reported by Wennberg et al [38] which was described in §3.2.15 of Report 1 [2] (the few remaining controls coming from the MONICA study, see §3.2.7). Results from this study as reported by Lundqvist et al [39] were included in PNL’s snus review [1]. Here, the more recent report by Norberg et al [40] is preferred, as it covers a longer period and provides sex- and age-specific results (although not for the 30-year olds who were included in the all-ages results from Lundqvist [39]). Intermittent smokers are categorized with the current smokers.

Based on a series of cross-sectional analyses, the sex-, age- and period-specific joint distribution of tobacco use is shown in Tables 1, 2 and 4. The ratio of switchers (current snus/former smoker) to continuing exclusive smokers (S1:N1) was as follows:

| Year |  | Men |  |  |  | Women |  |  |
| --- | --- | --- | --- | --- | --- | --- | --- | --- |
|  | Age: | 40 | 50 | 60 |  | 40 | 50 | 60 |
| 1990-1995 |  | 0.98 | 0.56 | 0.60 |  | 0.06 | 0.01 | 0.01 |
| 1996-2001 |  | 1.64 | 1.21 | 0.91 |  | 0.25 | 0.09 | 0.04 |
| 2002-2007 |  | 2.61 | 1.89 | 1.32 |  | 0.78 | 0.31 | 0.13 |

As can be seen, for both men and women, the ratio has increased over time within each age group, and is generally higher for the younger age groups. A similar pattern is seen with the alternative definition (S1:N2, Table 5). Results are also available by level of education (not shown).

A further analysis included those who had participated twice – initially in 1990-97 at ages 30, 40 or 50, and followed up 10 years later in 2000-07. Details of the joint distribution for the two time points, effectively giving a 2×2 table of current/non use at baseline by a similar 2×2 table at follow-up, are shown in Table 9. At follow-up, the distribution of tobacco use and the ratio of switchers to continuing smokers were as follows:

| Sex | Baseline | Follow-up |  |  |  | Ratio |  |  |
| --- | --- | --- | --- | --- | --- | --- | --- | --- |
|  |  | Switch to snus only | Continued smoking | |  | S4:N3 | S5:N4 | S5:N5 |
|  |  |  | Smoke only | Smoke only or dual use |  |  |  |  |
| Men | smoke only | 229 | 916 | 1049 |  | 0.25 |  |  |
|  | smoke only or dual | 573 | 972 | 1368 |  |  | 0.59 | 0.42 |
|  |  |  |  |  |  |  |  |  |
| Women | smoke only | 255 | 1886 | 1967 |  | 0.14 |  |  |
|  | smoke only or dual | 311 | 1893 | 2003 |  |  | 0.16 | 0.16 |

### 3.2.16 Swedish Medical Birth Register

This nationwide study [41-43] was described in §3.2.16 of Report 1 [2], and results are included in Table 2.

### 3.2.17 Case-control study of gastric cancer

This study [44], conducted in five counties in northern and central Sweden, was described in §3.2.17 of Report 1 [2], and results are included in Tables 1, 4 and 5.

### 3.2.18 Eurobarometer

Results for males from the Swedish part of the 2002 EU-wide Eurobarometer survey [45] were presented in PNL’s snus review [1] and are reproduced in Table 2. Both chewing tobacco and snuff were enquired about, but in the Swedish context, this will effectively be equivalent to snus use. There were too few female smokeless tobacco users (6 exclusive users and 3 dual users) for useful analysis. Results from other waves of this series and age-specific results for men have not been added, as the surveys have been criticised for having small sample sizes and generating “estimates that are in some cases widely discrepant from more substantive national sources” [46]

### 3.2.19 Swedish Twin Registry - STAGE

The Study of Twin Adults : Genes and Environment (STAGE) study recruited a younger cohort of twins, born 1959-1985, than the SALT study (see §3.2.4). Results reported by Furberg et al [47] were presented in PNL’s snus review [1] and are reproduced in Table 1.

### 3.2.20 Telephone survey of smokers

Gilljam and Galanti [48] reported on a cross-sectional study conducted in 2000, which recruited national samples of current and former daily smokers aged 25-55. The mean numbers of cigarettes smoked by the 424 male and 561 females current smokers are shown in Table 3. The authors also stated that:

“The proportion of current smokers smoking less than 10 cigarettes/day was newly twice as high among users of snus than among non-users (44% versus 24%, respectively, data not shown). Moreover, among smokers of less than 20 cigarettes a day there was an inverse relation between amount of snus consumed in a week and number of cigarettes smoked in a day (data not shown)”

They also reported that for 7.1% of current smokers and 4.6% of former smokers, smoking reduction was the main reason for starting using snus.

Among the male current smokers, 55% had never used snus, while among the former smokers 29% were current snus users (i.e. switchers). However as the study design was to recruit equal numbers of current and former smokers, the ratio of continuing smokers to switchers cannot be estimated. Equivalent figures for the females were 86% and 3% respectively.

### 3.2.21 Surveys of 21-year olds in northern town

Novo et al [49] described two cross-sectional studies conducted among 21-years old in an industrial town in northern Sweden in 1986 and 1994. Results were presented in PNL’s snus review [1] and are reproduced in Table 2. Results for women are not presented as there were only 1 and 10 dual users respectively.

### 3.2.22 NTS surveys

A series of nationally representative surveys were carried out in the 1970s-1980s by the NTS (Nationalfoereningen foer upplysning om tobakens skadeverknignar, Swedish National Smoking and Health Association). Results for men for the years 1985, 1986 and 1987 from three publications by lead author Ramström [50-52] were presented in PNL’s snus review [1] and are reproduced in Tables 1 and 2, with further details in Table 4 and 5. There were too few women dual users for useful analysis.

Note that in Table 2, regular and occasional smokers have been combined, as have regular and occasional snus users. However this was not possible for the results in Tables 1, 4 and 5, which refer only to regular smoking/snus use. The results shown have been derived from percentage distributions, and may be subject to rounding error.

Although no results are available for the mean number of cigarettes smoked, the percentage of male heavy smokers (smoking 13 or more cigarette per day) by snus use, shown in the table below, show a consistent tendency for snus users to be less likely to be heavy smokers than those who do not use snus.

|  |  | Current snus use | | | |
| --- | --- | --- | --- | --- | --- |
|  |  | Daily | Occasional | Daily or occasional | None |
| 1985 | 18-34 | 31.3 (16) | 52.4 (21) | 43.2 (37) | 65.6 (61) |
|  | 35-70 | 45.5 (11) | 43.5 (23) | 44.1 (34) | 62.9 (140) |
|  |  |  |  |  |  |
| 1986 | 18-34 | 20.0 (10) | 42.1 (19) | 34.5 (29) | 65.2 (69) |
|  | 35-70 | 50.0 (8) | 77.8 (18) | 69.2 (26) | 57.4 (148) |
|  |  |  |  |  |  |
| 1987 | 18-34 | 18.8 (32) | 72.2 (18) | 38.0 (50) | 76.6 (47) |
|  | 35-70 | 35.7 (14) | 69.2 (13) | 51.9 (27) | 61.5 (143) |
|  |  |  |  |  |  |
|  |  | Daily snus use | | | |
|  |  | Current | Ex | Ever | Never |
| 1987 | 18-34 | 18.8 (32) | 90.0 (20) | 46.2 (52) | 68.9 (45) |
|  | 35-70 | 35.7 (14) | 50.0 (20) | 44.1 (34) | 63.5 (137) |

Note: Smokers of pipe/cigar only are excluded. The numbers in brackets are the number of smokers on which the percentages were based..

The number of female dual users was too small for useful analysis.

From the 1987 survey [52], information was available on the sequence of starting among dual users. As shown in the table below virtually all older (age 35-70) dual users smoked first, but almost 30% of men aged 18-34 used snus first.

| Age |  |  | Males |  |  | Females |  |
| --- | --- | --- | --- | --- | --- | --- | --- |
|  |  |  | Snus first | Smoke first |  | Snus first | Smoke first |
| 18-34 | N^a^ |  | 32 | 79 |  | 2 | 6 |
|  |  |  | (28.8%) | (71.2%) |  | (25.0%) | (75.0%) |
|  |  |  |  |  |  |  |  |
|  | Smoking^b^ | Current daily | 21 | 28 |  |  |  |
|  |  |  | (65.6%) | (35.4%) |  |  |  |
|  |  | Current occasional | 5 | 20 |  |  |  |
|  |  |  | (15.6%) | (25.3%) |  |  |  |
|  |  | Ex | 6 | 31 |  |  |  |
|  |  |  | (18.8%) | (39.2%) |  |  |  |
|  |  |  |  |  |  |  |  |
|  | Snus^b^ | Current daily | 17 | 53 |  |  |  |
|  |  |  | (53.1%) | (67.9%) |  |  |  |
|  |  | Current occasional | 12 | 3 |  |  |  |
|  |  |  | (37.5%) | (3.8%) |  |  |  |
|  |  | Ex | 3 | 22 |  |  |  |
|  |  |  | (9.4%) | (28.2%) |  |  |  |
|  |  |  |  |  |  |  |  |
| 35-70 | N^a^ |  | 1 | 97 |  | 0 | 4 |
|  |  |  | (1.0%) | (99.0%) |  | (0.0%) | (100.0%) |
|  |  |  |  |  |  |  |  |
|  | Smoking^b^ | Current daily |  | 43 |  |  |  |
|  |  |  |  | (44.3%) |  |  |  |
|  |  | Current occasional |  | 8 |  |  |  |
|  |  |  |  | (8.2%) |  |  |  |
|  |  | Ex |  | 46 |  |  |  |
|  |  |  |  | (47.4%) |  |  |  |
|  |  |  |  |  |  |  |  |
|  | Snus^b^ | Current daily |  | 42 |  |  |  |
|  |  |  |  | (43.8%) |  |  |  |
|  |  | Current occasional |  | 16 |  |  |  |
|  |  |  |  | (16.7%) |  |  |  |
|  |  | Ex |  | 38 |  |  |  |
|  |  |  |  | (39.6%) |  |  |  |

Note: Dual users are those who had ever smoked daily and had used snus daily, for at least 6 months. Excluding subjects with partial missing data. Too few female dual users or male snus first users aged 35-70 for useful analysis.

^a^ %s are of all dual users (i.e. across the row)

^b^ %s are within the column

Similar information (although less detailed) was also presented by narrower age groups, by area of residence and by education.

Continuing smokers can also be compared with switchers (although it is not possible to exclude those who have also given up snus use):

|  |  | Switcher^a^ | Non-switcher^b^ | Switchers: non-switchers  S3:N1 |
| --- | --- | --- | --- | --- |
| Males | 18-34 | 51 | 52 | 0.98 |
|  | 35-70 | 54 | 171 | 0.32 |
|  |  |  |  |  |
| Females | 18-34 | 3 | 131 | 0.02 |
|  | 35-70 | 2 | 169 | 0.01 |

^a^ Ex daily smoker (including current occasional ex daily), ever daily snus, started smoking first.

^b^ Current daily smoker, never daily snus.

Note that the apparent discrepancies in Table 5 for values of N1 probably arises from rounding errors using results derived from different tables in the original source.

### 3.2.23 Your country and your life survey

Results from this nationally representative postal survey conducted in 2001-2003 were reported by Ramström and Foulds [53]. As no women were dual users, results were presented in PNL’s snus review [1] for males only, and are reproduced in Tables 1 and 2, with further details in Table 4.

Among the men who ever either smoked or used snus daily, the distribution including the order of starting for dual users can be summarized as follows:

| Ever used snus, never smoked | Ever smoked, never snus | Ever smoked and used snus |  |
| --- | --- | --- | --- |
|  |  | Snus first | Smoked first |
| 402 | 888 | 100 | 338 |
| (23.3%) | (51.4%) | (5.8%) | (19.6%) |

Various definitions of “switchers” (Table 5) can be compared with continuing smokers:

| Definition of switcher | Current smoker (never snus) (N1) | Switchers | Ratio |
| --- | --- | --- | --- |
| Started by smoking, currently snus only (S2) | 391 | 207 | 0.53 |
| Ever smoked, current snus only (S1) | 391 | 263 | 0.67 |
| Started by smoking, ever snus (S3) | 391 | 338 | 0.86 |

### 3.2.24 Health on equal terms

Health on equal terms (Hälsa på lika villkor) is a series of national postal surveys by Statens folkhälsoinstitut (Swedish National Institute of Public Health) [54]. The survey has been conducted annually since 2004, and results from PNL’s snus review [1] for 2004 and 2009 are reproduced in Table 2. The numbers of subjects shown are estimated from percentages given only as whole numbers, so may be subject to substantial rounding error. There were too few dual users among the women for useful analysis.

### 3.2.25 Stockholm City Fire Brigade

Results from a 1993 study of the Stockholm City Fire Brigade were reported in various papers by lead author Bolinder [55-58]. 151 men aged 35-60 participated.

The distribution by tobacco use was as follows:

|  | |  | Snus Use^a^ | | |
| --- | --- | --- | --- | --- | --- |
|  |  | | Current | Former | Never |
| Smoking^b^ | Current | | 5 | 2 | 26 |
|  |  | | (3.3%) | (1.3%) | (17.2%) |
|  | Former | | 21 | 26 | ← |
|  |  | | (13.9%) | (17.2%) |  |
|  | Never | | 29 | ↑ | 42 |
|  |  | | (19.2%) |  | (27.8%) |

^a^ for 6 months, see also text below

^b^ daily for 5 years

However the definition of snus use for a dual user (namely daily or occasional) differed from that for a sole user (daily), and one of the papers [56] implied that all the dual users were in fact occasional snus users. Consequently the OR for current use cannot sensibly be calculated and is not included in summary Table 1. However this problem does not affect the ratio of switchers to continuing smokers, which refers only to daily smoking/snus use, and is 21 / 26 = 0.81 using definition S1:N1, or 21 / 28 = 0.75 using definition S1:N2 (Tables 4 and 5).

### 3.2.26 Stockholm Public Health survey

Engström et al [59] reported on the 2006 wave of this study. Subjects were contacted by post and invited to complete the questionnaire either on paper or online. There were 34707 participants aged 18-84. The distribution of joint current use is shown in Table 2. ORs (not shown) for exclusive smokers and users, and dual users, relative to non-users, and for exclusive snus and dual users relative to exclusive smokers were also presented for a range of lifestyle factors.

Hansson et al [60] reported on a 5-year follow-up of men originally recruited in the 2002 wave. There were 9954 participants, giving a 76% retention rate at follow-up. There were differences in the definitions of smoking/use between the two questionnaires, but these were minor (baseline : daily for 6 months, follow-up : more or less daily for a year). Changes during the follow-up period in snus use and smoking were reported for those only ever reporting one product, and the number of those consistently reporting never use were also reported. All others were combined in a single category of “other” tobacco users, which at first sight must comprise all dual users (current and former). Thus the joint distribution of tobacco use at follow-up appears as follows:

|  | |  | Snus Use^a^ | | |
| --- | --- | --- | --- | --- | --- |
|  |  | | Current | Former | Never |
| Smoking^b^ | Current | | 2666 | ← | 729 stable+  56 starters =785 |
|  |  | | (26.8%) |  | (7.9%) |
|  | Former | | ↑ | ⭦ | 1541 stable+ 284 quitters =1825 |
|  |  | |  |  | (18.3%) |
|  | Never | | 445 stable + 52 starters =497 | 126 stable+ 178 quitters =304 | 3877 |
|  |  | | (5.0%) | (3.1%) | (38.9%) |

“stable” = same at baseline and follow-up; “starters” or “quitters” refers to change during the follow-up period

Based on this table, the OR for ever use is 4.94 (4.50-5.43) (Table 1). However, the authors make no mention of any subjects giving inconsistent replies (e.g. current or former smoker at baseline but never smoker at follow-up), so it is possible that such subjects may have inflated the “other” category, casting doubt on the validity of this OR.

These results are also available stratified by alcohol consumption, breakfast habits, education, fruit consumption and physical activity. The lack of detail for joint users prevents any useful analysis of the sequence of starting or switching.

### 3.2.27 Göteborg Public Dental Service study

Hirsch et al [61] reported results of a study conducted in 1986 at the annual dental check-up of patients of 9 clinics in central Göteborg. 836 patients were aged 18-19, among whom the joint distribution was reported as:

|  |  | Snus use |  |
| --- | --- | --- | --- |
|  |  | Ever | Never |
| Smoking | Ever | – | 209 |
|  |  |  | (25.0%) |
|  | Never | 110 | 517 |
|  |  | (13.2%) | (61.8%) |

The authors commented that the relatively high socioeconomic status of the districts covered may have contributed to the low prevalence of snus use. However in their definitions of categories they did not mention the possibility of dual use, and therefore the apparent absence of any dual users should be regarded with caution. (Table 1)

### 3.2.28 Skåne public health survey

Lindström [62] reported on the 2004 Skåne public health survey, a postal survey of randomly selected 18-80 year olds.

Results on the joint distribution of tobacco, based on 11855 men and 14050 women, were as follows:

|  |  | Men |  |  | Women |  |
| --- | --- | --- | --- | --- | --- | --- |
|  |  | Snus Use^a^ |  |  | Snus Use^a^ |  |
|  |  | Yes | No |  | Yes | No |
| Smoking | Current^b^ | 451 | 1813 |  | 73 | 3061 |
|  |  | (3.8%) | (15.3%) |  | (0.5%) | (21.8%) |
|  | Former^c^ | 925 | 2491 |  | 173 | 2882 |
|  |  | (7.8%) | (21.0%) |  | (1.2%) | (20.5%) |
|  | Never^c^ | 936 | 5239 |  | 78 | 7783 |
|  |  | (7.9%) | (44.2%) |  | (0.6%) | (55.4%) |

^a^ Undefined, but assumed from context to be current

^b^ Daily or intermittent

^c^ Undefined whether daily or daily and intermittent

The ORs for current use are 1.03 (0.92-1.16) for men and 1.01 (0.78-1.32) for women (Tables 2, 4 and 5).

### 3.2.29 Stockholm family history of diabetes study

Persson et al [63] reported on a population-based cross-sectional study conducted in four suburban municipalities of Stockholm in 1992-94. An initial screening questionnaire identified men with a family history of diabetes, all of whom were invited to participate, along with an equally sized random sample of those without family history. The eventual sample comprised 3128 men aged 35-56, 52% with a family history of diabetes. The following joint distribution of daily tobacco use could be derived:

|  |  | Snus use |  |  |
| --- | --- | --- | --- | --- |
|  |  | Current | Former | Never |
| Smoking^a^ | Current | 691^b^ | ← | 517 |
|  |  | (24.8%) |  | (18.6%) |
|  | Former | ↑ | ⭦ | 503 |
|  |  |  |  | (18.1%) |
|  | Never | 121 | 56 | 895 |
|  |  | (4.3%) | (2.0%) | (32.2%) |

^a^ Cigarettes

^b^ Estimated by subtraction from all other categories. As the numbers of subjects in the cigarette and snus analyses differ by 2 due to missing data, the correct figure may be 689

The OR for ever smoking is 3.43 (2.84-4.13) (Tables 1 and 4)

### 3.2.30 Värmland ice hockey players

Rolandsson and Hugoson [64] reported results from a longitudinal study among boys aged 12-19 recruited in 1998 at three ice-hockey clubs in Värmland, with 73% followed up in 2001. As this report is concerned with adults, we report here results for the 95 subjects aged 18-22 at the follow-up.

|  |  | Snus use^a^ |  |
| --- | --- | --- | --- |
|  |  | Current | Non |
| Smoking^b^ | Current | 10 | 2 |
|  |  | (10.5%) | (2.1%) |
|  | Non | 27 | 56 |
|  |  | (28.4%) | (58.9%) |

^a^ any current use (from “every day” to “hardly ever”)

^b^ undefined

The OR for current use is 10.37 (2.12-20.66) (Table 2)

### 3.2.31 Älvsborg county study

Salonen et al [65] reported on a cross-sectional population study in the north of Älvsborg county^[[1]](#footnote-1)^, with clinical examination of 920 subjects aged 20+. All the women tobacco users were exclusive cigarette smokers. Among the 448 men, the joint distribution of tobacco use was as follows:

|  |  | Snus use |  |
| --- | --- | --- | --- |
|  |  | Current | Non |
| Smoking | Current | 16 cigarette+  5 pipe  =21 | 103 cigarette+  32 pipe+  2 cigarette&pipe  =137 |
|  |  | (4.7%) | (30.6%) |
|  | Non | 58 | 232 |
|  |  | (12.9%) | (51.8%) |

The OR for current use (based on smoking any product) was 0.61 (0.36-1.05), and this varied little if pipe smokers were omitted or treated as non-smokers (results not shown). (Table 2)

### 3.2.32 Vara municipality study

Sundbeck et al [66] reported a study in the municipality of Vara (SW Sweden) within the Skaraborg project. A population survey was conducted in 2001-03 with 1811 participants aged 30-75. Only 8 women used snus, so further analysis was restricted to men. As well as those with incomplete information or using chewing tobacco or nicotine gum, the study criteria excluded those currently using more than one type of tobacco. Among the 834 remaining men, the joint distribution was as follows:

|  |  | Snus use^a^ |  |
| --- | --- | --- | --- |
|  |  | Current | Non |
| Smoking^b^ | Current | excluded^c^ | 109 |
|  | Former | 116 | 189 |
|  | Never | 63 | 357 |

^a^ weekly

^b^ cigarette, cigar or pipe, daily

^c^ 36 persons were excluded as they used chewing tobacco, nicotine gum or more than one sort of tobacco, but it is not known how many of these were men, or, of those, how many were dual snus users/smokers.

Because of the exclusion of dual users, it is not possible to express this as a percentage distribution, or to calculate the OR for joint use. However the relative frequency of switchers to continuing smokers (S1:N2) is 116 / 109 = 1.06. Note that the definition of continuing smokers may have used snus in the past. (Tables 4 and 5)

It was also reported that the average snus consumption by the 63 users who had never smoked was 3.7 cans/week, while for the 116 former smoking snus users it was 3.4. Again, due to the exclusion of the dual users, this result is not comparable to those given in Table 3.

### 3.2.33 Atherosclerosis and Insulin Resistance Study (AIR)

Wallenfeldt et al [67] reported a sub-study of the AIR study. Of 818 original participants (men, aged 58, of Swedish ancestry, and without various cardiovascular diseases or drug therapies), a subsample of 391 was drawn, comprising all those in the lowest and highest quintiles for insulin sensitivity and a 20% sample of those in the intermediate quintiles. The date the study was conducted was not given. The joint distribution of tobacco use was as follows:

|  |  | Snus use^a^ |  |  |
| --- | --- | --- | --- | --- |
|  |  | Current | Former | Never |
| Smoking^a^ | Current | 14 | 12 | 70 |
|  |  | (3.6%) | (3.1%) | (17.9%) |
|  | Former | 32 | 19 | 101 |
|  |  | (8.2%) | (4.9%) | (25.8%) |
|  | Never | 2 | 2 | 139 |
|  |  | (0.5%) | (0.5%) | (35.5%) |

^a^ daily

The OR for ever use is 1.31 (0.67-2.56), and for current use is 15.65 (5.59-43.82). The ratio of switchers to continuing exclusive smokers (S1:N1) is 0.46 (or using the alternative definition S1:N2 is 0.39). (Tables 1, 2, 4 and 5)

Although current consumption was not reported, estimated lifetime consumption was reported as follows:

|  |  | Snus use |  |  |
| --- | --- | --- | --- | --- |
|  |  | Current | Former | Never |
| Smoking | Ever smoked  (cigarette-years^a, b^) | 400 ± 309 | 627 ± 554 | 293 ± 397 |
|  |  |  |  |  |
|  |  | Smoking |  |  |
|  |  | Current | Former | Never |
| Snus use | Ever snus  (snus years^a, c^) | 87 ± 232 | 178 ± 419 | 23 ± 181 |

^a^ Mean ± SD

^b^ Years smoked × cigarettes per day

^c^ Years taken snus × grams snus per day

Note that it is not clearly stated whether these means are calculated based on all the subjects, or based only on the smokers/users.

### 3.2.34 Stockholm 60-year olds study

Wändell et al [68] reported on a population-based study among 60-year olds in Stockholm county in 1997-1999. After excluding women due to “low prevalence of smokeless tobacco use among women in the study base”, and 113 men with diabetes, the sample comprised 1859 men. The joint distribution of tobacco use was as follows:

|  |  | Snus use^a^ |  |  |
| --- | --- | --- | --- | --- |
|  |  | Current | Former | Never |
| Smoking^b^ | Current | 27 | –^c^ | 360^d^ |
|  |  | (1.5%) |  | (19.4%) |
|  | Former | 113 | –^c^ | 737^d^ |
|  |  | (6.1%) |  | (39.6%) |
|  | Never | 16^e^ | 12^e^ | 594 |
|  |  | (0.9%) | (0.6%) | (32.0%) |

^a^ Undefined, but assumed weekly from context

^b^ Daily

^c,d,e^ Categories marked ^d^ are described simply as current smokers and ex smokers, and are assumed to be never snus users. Similarly those marked ^e^ are described as current or ex snuffers and are assumed to be never smokers. Categories marked ^c^ are thus implied to be zero.

The OR for ever use is 2.71 (1.78-4.11), and for current use is 0.78 (0.51-1.20). The ratio of switchers to continuing exclusive smokers (S1:N1) is 0.31. (Tables 1, 2, 4 and 5)

### 3.2.35 Säve military conscripts

Screening prior to military conscription is compulsory for 18-19 year old men in Sweden, and the centre at Säve serves the South West of the country. Wennmalm et al [69] reported on a study carried out there on a random sample in one year (although it was not stated which year). After excluding subjects taking certain medications or with pre-existing acute or chronic disease, and also excluding 9 subjects who stated no tobacco use but had high levels of urinary cotinine, results were available for 577 men. The joint distribution of tobacco use was as follows:

|  |  | Snus use |  |  |  |
| --- | --- | --- | --- | --- | --- |
|  |  | Current |  | Former | Never |
| Smoking^a^ | Current | 30 |  | 43 | ← |
|  |  | (5.2%) |  | (7.5%) |  |
|  |  |  |  |  |  |
|  | Former | 127 |  | 33 | ← |
|  |  | (22.0%) |  | (5.7%) |  |
|  | Never | ↑ |  | ↑ | 344 |
|  |  |  |  |  | (59.6%) |

^a^ Cigarette smoking, except one man who smoked a pipe as well as smoking cigarettes and using snus.

The OR for current use was 2.07 (1.25-3.44) (Table 2).

Mean daily consumption of cigarettes and snus were also reported (Table 3), together with lifetime consumption and urinary cotinine as follows:

|  | Snus  only | Smoking  only | Snus and  smoking | |
| --- | --- | --- | --- | --- |
|  |  |  |  |  |
|  | (grams) | (cigs) | (grams) | (cigs) |
| Current consumption^a^ | 25 ± 1 | 12.2 ± 0.8 | 27 ± 3 (108%) | 7.8 ± 1.3 (64%) |
|  |  |  |  |  |
|  | (kgs) | (cigs×1000) | (kgs) | (cigs×1000) |
| Lifetime consumption^a^ | 29.1 ± 2.7 | 17.4 ± 2.0 | 43.4 ± 8.7 (149%) | 9.1 ± 2.1 (52%) |
|  |  |  |  |  |
|  | ng/ml | ng/ml | ng/ml |  |
| Cotinine in urine^b^ | 1210  (3.1-4280) | 1560  (570-3450) | 1773  (840-2800) |  |

^a^ Mean ± SE

^b^ Median (range)

### 3.2.36 Stockholm county 31-40 year olds

Wickholm et al [70] reported a population-based study among 31-40 year olds in the Stockholm region around 1985. The sample size was 823 men and 851 women. The joint distribution was reported for both ever and current use, but the full 3×3 table cannot be derived from this information.

|  |  |  | Men |  |  | Women |  |
| --- | --- | --- | --- | --- | --- | --- | --- |
|  |  | Snus use: | Ever | Never |  | Ever | Never |
| Smoking | Ever |  | 90 | 432 |  | 9 | 540 |
|  |  |  | (10.9%) | (52.5%) |  | (1.0%) | (63.5%) |
|  | Never |  | 51 | 250 |  | 3 | 299 |
|  |  |  | (6.2%) | (30.4%) |  | (0.3%) | (35.1%) |
|  |  |  |  |  |  |  |  |
|  |  | Snus use: | Current | Non |  | Current | Non |
| Smoking | Current |  | 36 | 278 |  | 4 | 331 |
|  |  |  | (4.4%) | (33.8%) |  | (0.5%) | (38.9%) |
|  | Non |  | 75 | 434 |  | 7 | 509 |
|  |  |  | (9.1%) | (52.7%) |  | (0.8%) | (59.8%) |

The OR for ever use was 1.02 (0.70-1.49) in men, and 1.66 (0.45-6.18) in women. For current use it was 0.75 (0.49-1.15) in men and 0.88 (0.26-3.03) in women. (Tables 1 and 2; females omitted from tables due to low numbers)

## 3.3 Overall analysis

### 3.3.1 Association between ever smoking and ever snus use

Table 1 summarizes the available results relating ever snus use to ever smoking from the 19 studies that have provided data. The Construction Workers study provides results from essentially the same population based on slightly differing recruitment periods, and the VIP study reports cross-sectional results at different time points from a cohort study, while the MONICA study reports independent results from surveys carried out at different time points (with results for different smoking definitions available at one time point). One study (Göteborg Public Dental Service) which reported no dual users is not considered further.

Based on the ORs and 95% CIs provided, a combined meta-analysis estimate was calculated. From studies providing multiple estimates, inclusion in the meta-analysis was decided (as outlined in §2.8) on the following basis:

- using the all age estimates in preference to estimates by age, if both were available
- using sex-specific estimates for both sexes if available (3 studies), otherwise sexes-combined estimates (2 studies) or estimates for males (13 studies)
- using the apparently more reliable data from the Construction Workers study [11], and the more consistently reported data from the MONICA study [29]
- omitting the younger subjects (40 or 50) of the first wave (1990-95) of the VIP study, as a large proportion of the same subjects are also included in the last wave (i.e. as 50 or 60 year-olds in the 2002-2007 wave), (Because the study waves as reported were not at exactly 10 years intervals, a small proportion of subjects will have contributed to more than one OR included in the meta-analysis (i.e. 40 year olds in 1996-7 became 50 year olds in 2006-7, and similarly for the 50 year olds), but this “double-counting” is deemed minor.)

As shown in Table 10, this gave an overall fixed-effect estimate of 1.29 (1.27- 1.30) with the heterogeneity chisquared equal to 8047 on 35 degrees of freedom (p < 0.001), and a random-effects estimate of 3.13 (2.28-4.30).

As also shown in Table 10, the meta-analyses was repeated, separately for the ORs in males, in females and for the sexes combined, showing that the estimate for females (5.03 (4.69-5.40) was substantially higher than the estimates for males (1.22 (1.21-1.24) or from the few studies with only sexes-combined ORs (0.93 (0.70-1.22). Further meta-analyses looking for variation by age, and various aspects of the study were carried out using only the data for males. As can be seen, the OR is greater than 1 for every level of every factor, significantly so with only two exceptions. No single factor explains the variation, with heterogeneity remaining significant within each level of each factor.

The estimates are higher for

- later studies
- studies in the north of Sweden
- studies that included any frequency of snus use than for studies of regular use. The result was similar when comparing studies of any frequency of smoking with studies of regular smoking (not shown), many studies using the same definition for both products

but do not vary significantly with age, or between studies of cigarette smoking specifically and studies of any smoking. Multivariate analysis might more clearly identify independent factors affecting the association between ever smoking and ever snus use, but has not been attempted in this report.

Note that analysis by some factors is limited by the decisions that were made as to which data to extract (§2.4). It would be possible to extract data using alternative definitions for some studies, for instance by not combining regular and occasional snus users, or to extract regional results from the ULF study. As well as providing more estimates relevant to specific factor levels, this would allow within-study testing of this factor, but this has not been attempted.

The data in Table 1 can also be used to summarize estimates of the percentage of dual ever users. Means, ranges and standard deviations of the estimates corresponding to the same selection criteria as used for the meta analyses are shown in Table 11.

The percentage varies from 3% to 44%, with higher values for males than females. Within the male studies, values are higher for later studies, studies in the North, and (as would be expected) for studies looking at any use rather than regular use only.

### Association between current smoking and current snus use

Table 2 similarly summarizes the available evidence relating current snus use to current smoking from 22 studies, six of which provide data at multiple time points (five independent and the sixth, VIP, as described in the previous section).

Selection of estimates to include in meta-analysis, and the factors investigated, were similar to those used for ever smoking/use (§3.3.1), and rResults of meta-analyses are summarized in Table 12. The overall meta-analysis estimate is 0.96 (0.93-0.99) from the fixed-effect model, and 1.05 (0.95-1.16) from the random-effects model. As previously, no factor explains the heterogeneity which, although lower than in the previous analysis, is again significant at almost every level of every factor. However, unlike the previous analysis, differences between the levels of the various factors are generally not significant here, with no significant variation in the OR seen by sex or, for males, by period, age, region or regularity of use. The only significant difference was that studies of cigarettes smoking specifically had lower ORs than studies of any/unspecified smoking. Given the small number of pipe/cigar smokers in Sweden, and given that some studies did not clearly specify the smoking product, this difference is unlikely to be meaningful.

Unlike the situation for ever smoking/use, where Table 10 showed a significant positive association in virtually every one of the random-effect estimates, the corresponding results in Table 12 seem consistent with a lack of association between current smoking and current snus use.

Percentages of dual current users are summarized in Table 13. As for ever use, values are higher for males than females. However within the male studies, higher values are now seen for earlier studies and at younger ages, but with little difference by region.

### Tobacco consumption in single and dual users

Table 3 summarizes relevant data from eight studies. All the studies allow comparison of daily cigarette consumption in dual users and smokers who do not use snus. Consumption is always less in dual users, with the all age estimates for men (or sexes combined in one study) ranging from 54% to 98% (mean 75%) of the consumption of smokers who do not use snus. Results from the NTS study showing lower percentages of heavy smokers among snus users (§3.2.22) are consistent with this.

Four of the studies allow comparison of snus use in dual users to that of snus users who do not smoke. Apart from the study of military conscripts, the results indicate a somewhat lower snus usage in the dual users.

While one general conclusion is that dual users smoke less cigarettes a day than do smokers who do not use snus, and use less snus than users who do not smoke, it seems that dual users use at least as much total tobacco. Eliasson et al [31] gave estimates for the 1990 MONICA study of 28.2 g for dual users, 22.9 g for exclusive snus users and 16.5 g for exclusive smokers. Applying the same conversion factors of 1 g per cigarette and 50 g per can of snus to the other studies, estimates of total consumption (g) are as follows:

|  | Snus only | Smoke only | Snus and smoking |
| --- | --- | --- | --- |
| Construction workers (all ages) | 22 | 12 | 25 |
| Kalixanda | 22.9 | 11.5 | 21.9 |
| MONICA 1986-1999 | 21 | 15.8 | 23.3 |
| MONICA 1990 | 22.9 | 16.5 | 28.2 |
| Save military conscripts | 25 | 12.2 | 34.8 |

This shows total consumption by dual users similar to or higher than consumption by exclusive snus users, and considerably higher than by exclusive smokers. However there is uncertainty in these estimates. For instance, using the alternative estimate of 0.65 g per cigarette [3] generally reduces the estimated total consumption for dual users to below that for the exclusive snus users. We do not have adequate information on the weight of snus per can for the relevant study dates to comment on the validity of the conversion factor for snus, but it can be noted that many current snus brands are sold in cans of less that 50 g. It should also be noted that both construction workers and military conscripts have working conditions that may restrict opportunities for smoking, so that results from those studies are unlikely to be typical of the general population.

Some studies gave results on cumulative (lifetime) consumption by single and dual users (see §3.2 subsections 5, 14, 33, 35), but as the duration of usage of the products may well have differed, these results are of little relevance.

Results on levels of biomarkers of exposure are available from only 2 studies:

|  |  | Snus only | Smoke only | Snus and smoking |
| --- | --- | --- | --- | --- |
| MONICA 1990 [31] | Plasma cotinine (ng/ml)^a^ | 351 | 242 | 308 |
|  | Plasma nicotine (ng/nl)^a^ | 15.5 | 9.8 | 9.5 |
| Säve military conscripts | Urinary cotinine | 1210 | 1560 | 1773 |

No consistent pattern is evident, with the highest biomarker levels seen in the exclusive snus users from the MONICA study but in the dual users from the conscripts study, while the lowest level is seen in a different category for each of the three measures shown. Further details have been given in §3.2.7 and §3.2.35.

### More detailed joint distribution of smoking and snus

Table 4 gives, for 19 studies, a breakdown of the population jointly by smoking (current/ex/never), or by a 3x2 breakdown where the full 3x3 breakdown is not available. Numbers of subjects are shown in Table 4A, with percentages of the total population shown in Table 4B.

### 3.3.5 Switching ratios

Table 5 shows the ratios of switchers to non-switchers, i.e. the numbers of persons switching from smoking to snus, relative to those continuing to smoke. Much of the data is derived from that shown in Table 4, with the remainder taken from text tables in §3.2 (ULF (3.2.3), SALT (3.2.4), MONICA follow-up (3.2.8), NTS (3.2.22), Your country your life (3.2.23), and Stockholm City Fire Brigade (3.2.25)).

From cross-sectional studies that gave a full 3×3 distribution of smoking (current/ex/never) × snus use (current/ex/never), the definition a of switcher as “current snus/former smoker” (denoted *S1*) is used, together with two alternative definitions of a non-switcher ­ “current smoker/never snus” (denoted *N1*), or as “current smoker/never or former snus” (*N2*). The *N2* definition is also given from some cross-sectional studies which gave less than the full 3×3 table, so that *N1* could not be obtained. Other definitions as described in section 2.6 are given where available from studies that reported on the order of first use, or from longitudinal studies.

Interpretation is difficult due to the differing definitions available from the different studies. However, from those studies with results for both men and women, it can be seen that the ratio is lower for women than for men irrespective of the definitions available. Among men, for the studies reporting over a period of time, the ratio has generally increased over time, and for studies reporting by age, the ratio generally decreases with age. The ratio is generally below 1 (i.e. non-switchers outnumber switchers) except for studies conducted since around 2000, where it exceeds 1, occasionally exceeding 2 from recent studies of younger men.

Four studies presented more detailed data on switching, as shown in Table 6 (ULF study), Table 7 (SALT study), Table 8 (MONICA follow-up study), and Table 9 (VIP follow-up study).

Further analysis is beyond the scope of this report.

### 3.3.6 Sequence of initiation

Only three studies gave information on the sequence of initiation for the two tobacco products, and results are shown in §3.2.4 for the SALT study, §3.2. 22 for the NTS study and in §3.2.23 for the Your country you life study. All three studies showed that a large majority of male dual users reported having smoked first. This was around 70-80%, with the exception of the older age group (35-70) in the NTS study, where virtually all (99%) had smoked first. This difference is unlikely to be related to the age of the participants, which was similar to the SALT study (age 42-64), but may be related to the earlier date of the study (1987 compared with around 2000 for the other two), such that the subjects would have started their tobacco careers before the resurgence of snus use in the late 1960s [3] quoting [71].

# 4. Summary

Literature searches were carried out to identify studies in Sweden that had published information relevant to characterization of the dual use of snus and cigarettes. Studies in populations likely to have smoking and snus use habits that were highly atypical of the general population (e.g. alcoholics) were excluded. 34 separate studies were identified. From each study data were extracted relating to the joint distribution of snus and smoking, the amount used by dual users, switching from smoking to snus, and the sequence of initiating smoking and snus use. For studies including adolescents and young adults, attention was restricted, if possible, to those aged 18+ years.

**Association between ever smoking and ever use**

19 studies provided relevant information. There was clear evidence that those who had ever smoked were more likely to have ever used snus. Based on n = 36 estimates, the random-effects estimate of the odds ratio (OR) was 3.13 (95% CI 2.28-4.30). The association was stronger in females (5.49, 4.14-7.30, n = 9) than in males (2.80, 1.94-4.06, n = 25). Within males, estimates were higher in later studies, in studies in the north of Sweden, and in studies where the definition of snus use was based on any frequency of use, rather than regular use. These factors did not fully explain the marked heterogeneity between the OR estimates.

The frequency of dual use varied from 3% to 44% with a mean of 18.2% (SE 1.7%). Dual use was less common in females (9.4%) than in males (22.0%) and, within males, values were higher for later studies, studies in the North and studies looking at any use rather than regular use.

**Association between current smoking and current use**

22 studies provided relevant information. In contrast to the evidence for ever smoking/snus use, there was no clear evidence that those who currently smoked were more likely to currently use snus. Here the random-effect estimate was 1.05 (0.95-1.16, n = 56) and there was no marked variation in the estimates by any of the cofactors studied. Though there was unexplained heterogeneity between estimates, this was much less than for ever smoking/snus use.

The frequency of dual use varied from 0.1% to 12.4% with a mean of 3.5% (SE 0.4%). Dual use is much less common in females (0.9%) than in males (4.8%), and, within males, values were higher for earlier studies and at younger ages.

The association for ever smoking/snus use, but not for current smoking/snus use can be explained by some people avoiding tobacco, many of the rest trying both products and ultimately settling for one.

**Tobacco consumption in single and dual users**

8 studies provided relevant information. Cigarette consumption in dual users is less than in smokers who do not use snus, with the ratio varying from 54% to 98% (mean 75%). Four of the studies allow comparison of snus use in dual users to that in snus users who do not smoke. Apart from a study of military conscripts, the results indicate a somewhat lower snus usage in the dual users. Comparison of total tobacco use in dual users and exclusive smokers or snus users is complicated by difficulties in assigning appropriate values to the weight of tobacco in a cigarette and in a can of snus.

The studies we considered provided limited, and conflicting, evidence based on levels of biomarkers of exposure, in dual and exclusive users, allowing no clear conclusion.

**Switching ratios**

Based on more detailed breakdowns of the joint distribution of smoking and snus use, it was possible to derive estimates from 19 studies of the ratio of the number of people switching from smoking to snus, relative to those continuing to smoke. Interpretation of the data is complicated by the differing definitions available from the different studies. However, it is clear that the ratio is much lower for women than for men. Within men, the ratio generally decreases with age and increases over time. For studies conducted before the year 2000, non-switchers generally outnumber switchers, while for later studies switchers are in the majority, with switching ratios exceeding 2 from recent studies of younger men.

**Sequence of initiation**

Only 3 studies provided information. All these studies showed that a large majority of male dual users reported having smoked first. Generally this was about 70-80%, except for the older age group in an early study where virtually all (99%) had smoked first. Note that studies in adolescents would provide additional data here.

# Tables

## TABLE 1 Association of ever (vs never) snus use with ever (vs never) smoking

| Study^a^ | Year | Source | Sex | Age | Definitions | | Numbers of subjects | | | | | OR (95% CI) | |
| --- | --- | --- | --- | --- | --- | --- | --- | --- | --- | --- | --- | --- | --- |
|  |  |  |  |  |  | | Neither | Snus  only | Smoking  only | Snus and  smoking | |  | |
|  |  |  |  |  | Snus^b^ | Smok^c^ |  |  |  |  |  |  |  |
|  |  |  |  |  |  |  |  |  |  |  |  |  | |
| Construction workers (1) | 1978-93 | [11] | M | <24 | /R | A/R | 33508 | 21282 | 15044 | 8543 | (10.9%) | 0.89 (0.87-0.92) | |
|  |  |  |  | 25-34 |  |  | 20371 | 10200 | 27804 | 13914 | (19.2%) | 1.00 (0.97-1.03) | |
|  |  |  |  | 35-44 |  |  | 15048 | 3529 | 29355 | 11093 | (18.8%) | 1.61 (1.54-1.68) | |
|  |  |  |  | 45-54 |  |  | 10462 | 1313 | 20028 | 5601 | (15.0%) | 2.23 (2.09-2.38) | |
|  |  |  |  | 55+ |  |  | 8361 | 1135 | 16909 | 4274 | (13.9%) | 1.86 (1.74-2.00) | |
|  |  |  |  | Total^d^ |  |  | 87750 | 37459 | 109143 | 43425 | (15.6%) | 0.93 (0.92-0.95) | |
|  |  |  |  |  |  |  |  |  |  |  |  |  | |
| Construction workers (1) ^e^ | 1971-93 | [9,10] | M | ≤20 | /R | A/R | 16478 | 9561 | 7621 | 3962 | (10.5%) | 0.90 (0.86-0.94) | |
|  |  |  |  | 20-29 |  |  | 38656 | 20887 | 38056 | 19861 | (16.9%) | 0.97 (0.94-0.99) | |
|  |  |  |  | 30-39 |  |  | 19189 | 5201 | 35626 | 14030 | (18.9%) | 1.45 (1.40-1.51) | |
|  |  |  |  | 40-49 |  |  | 13294 | 2087 | 26967 | 7611 | (15.2%) | 1.80 (1.71-1.90) | |
|  |  |  |  | 50-59 |  |  | 10419 | 2063 | 23078 | 6202 | (14.9%) | 1.36 (1.28-1.43) | |
|  |  |  |  | 60+ |  |  | 3923 | 1133 | 8290 | 2186 | (14.1%) | 0.91 (0.84-0.99) | |
|  |  |  |  | Total^d^ |  |  | 101959 | 40932 | 139638 | 53852 | (16.0%) | 0.96 (0.95-0.98) | |
|  |  |  |  |  |  |  |  |  |  |  |  |  | |
| Kalixanda (2) ^e^ | 1998-2001 | [12] | M+F | 20-34 | /U | C/U | 36 | 20 | 28 | 10 | (10.6%) | 0.64 (0.26-1.59) | |
|  |  |  |  | 35-49 |  |  | 112 | 33 | 104 | 17 | (6.4%) | 0.55 (0.29-1.06) | |
|  |  |  |  | 50-64 |  |  | 147 | 45 | 154 | 29 | (7.7%) | 0.62 (0.37-1.03) | |
|  |  |  |  | 65+ |  |  | 137 | 14 | 88 | 15 | (5.9%) | 1.67 (0.77-3.62) | |
|  |  |  |  | Total 20+ |  |  | 432 | 112 | 374 | 71 | (7.2%) | 0.73 (0.53-1.02) | |
|  |  |  |  |  |  |  |  |  |  |  |  |  | |
| SALT (4) | 1998-2002 | [19] | M | 42-64 | /A | C/A | 6325 | 1509 | 6200 | 2608 | (15.7%) | 1.76 (1.64-1.90) | |
|  |  |  |  |  |  |  |  |  |  |  |  |  | |
| 2 AMI CC (6) | 1992-94 | [23] | M | 45-70 | /U | U/U | 598 | 40 | 940 | 232 | (12.8%) | 3.69 (2.60-5.24) | |
|  |  |  |  |  |  |  |  |  |  |  |  |  | |
| MONICA (7) | 1986 | [29] | M | 25-64 | /R | C/R | 282 | 97 | 237 | 191 | (23.7%) | 2.34 (1.74-3.16) | |
|  |  | [26] | M | 25-64 | /U | A/A | 208 | 34 | 311 | 256 | (31.6%) | 5.04 (3.38-7.50) | |
|  |  |  |  |  |  |  |  |  |  |  |  |  | |
|  | 1990 | [29] | M | 25-64 | /R | C/R | 253 | 130 | 207 | 176 | (23.0%) | 1.65 (1.24-2.22) | |
|  |  |  | F | 25-64 | /R | C/R | 437 | 16 | 318 | 23 | (2.9%) | 1.98 (1.03-3.80) | |
|  |  |  |  |  |  |  |  |  |  |  |  |  | |
|  | 1994 | [29] | M | 25-74 | /R | C/R | 269 | 97 | 196 | 185 | (24.8%) | 2.62 (1.93-3.56) | |
|  |  |  | F | 25-74 | /R | C/R | 353 | 16 | 383 | 33 | (4.2%) | 1.90 (1.03-3.51) | |
|  |  |  |  |  |  |  |  |  |  |  |  |  | |
|  | 1999 | [29] | M | 25-74 | /R | C/R | 237 | 122 | 124 | 194 | (28.7%) | 3.04 (2.22-4.16) | |
|  |  |  | F | 25-74 | /R | C/R | 362 | 15 | 307 | 55 | (7.4%) | 4.32 (2.39-7.81) | |
|  |  |  |  |  |  |  |  |  |  |  |  |  | |
| IBD CC Stockholm (11) | 1984-87 | [34] | M | 15-79 | /R | C/U | 61 | 11 | 63 | 10 | (6.9%) | 0.88 (0.35-2.22) | |
|  |  |  |  |  |  |  |  |  |  |  |  |  | |
| Uppsala (13) | 1973-74 | [35] | M | 15+ | /R | U/R | 3108 | 867 | 5309 | 692 | (6.9%) | 0.47 (0.42-0.52) | |
|  |  |  |  |  |  |  |  |  |  |  |  |  | |
| Oral cancer CC (14) | 1980-89 | [37] | M+F | ^f^ | /R | A/R | 144 | 27 | 138 | 45 | (12.7%) | 1.74 (1.02-2.96) | |
|  |  |  |  |  |  |  |  |  |  |  |  |  | |
| VIP (15) | 1990-95 | [40] | M | 40 | /U | U/A | 1513 | 517 | 1130 | 1811 | (36.4%) | 4.69 (4.14-5.31) | |
|  |  |  | M | 50 |  |  | 1471 | 251 | 1587 | 1326 | (28.6%) | 4.90 (4.20-5.70) | |
|  |  |  | M | 60 |  |  | 1244 | 125 | 1268 | 732 | (21.7%) | 5.75 (4.68-7.05) | |
|  |  |  | F | 40 |  |  | 2212 | 34 | 3024 | 325 | (5.8%) | 6.99 (4.89-10.00) | |
|  |  |  | F | 50 |  |  | 2423 | 10 | 2748 | 68 | (1.3%) | 6.00 (3.08-11.67) | |
|  |  |  | F | 60 |  |  | 2339 | 0 | 1557 | 20 | (0.5%) | – | |
|  |  |  |  |  |  |  |  |  |  |  |  |  | |
|  | 1996-2001 |  | M | 40 |  |  | 2012 | 931 | 799 | 1586 | (29.8%) | 4.29 (3.82-4.81) | |
|  |  |  | M | 50 |  |  | 1855 | 521 | 1607 | 2227 | (35.9%) | 4.93 (4.39-5.54) | |
|  |  |  | M | 60 |  |  | 1632 | 253 | 1469 | 1166 | (25.8%) | 5.12 (4.39-5.97) | |
|  |  |  | F | 40 |  |  | 2431 | 156 | 2465 | 734 | (12.7%) | 4.64 (3.87-5.56) | |
|  |  |  | F | 50 |  |  | 2735 | 19 | 3398 | 283 | (4.4%) | 11.99 (7.51-19.13) | |
|  |  |  | F | 60 |  |  | 2685 | 0 | 2227 | 60 | (1.2%) | – | |
|  |  |  |  |  |  |  |  |  |  |  |  |  | |
|  | 2002-2007 |  | M | 40 |  |  | 2676 | 1357 | 527 | 1489 | (24.6%) | 5.57 (4.95-6.28) | |
|  |  |  | M | 50 |  |  | 2158 | 902 | 1111 | 2171 | (34.2%) | 4.68 (4.20-5.20) | |
|  |  |  | M | 60 |  |  | 2027 | 417 | 1911 | 2064 | (32.2%) | 5.25 (4.65-5.93) | |
|  |  |  | F | 40 |  |  | 3319 | 390 | 1584 | 1000 | (15.9%) | 5.37 (4.71-6.13) | |
|  |  |  | F | 50 |  |  | 2766 | 73 | 3081 | 771 | (11.5%) | 9.48 (7.42-12.12) | |
|  |  |  | F | 60 |  |  | 2994 | 14 | 3331 | 272 | (4.1%) | 17.46 (10.18-29.95) | |
|  |  |  |  |  |  |  |  |  |  |  |  |  | |
| Gastric cancer CC (17) | 1989-1995 | [44] | M | 40-79 | /W | A/R | 217 | 36 | 370 | 156 | (20.0%) | 2.54 (1.70-3.79) | |
|  |  |  |  |  |  |  |  |  |  |  |  |  | |
| STAGE (19) | 2005-06 | [47] | M | 20-47 | /A | C/A | 2006 | 1360 | 1424 | 3763 | (44.0%) | 3.90 (3.56-4.27) | |
|  |  |  | F |  |  |  | 3441 | 422 | 4406 | 2251 | (21.4%) | 4.17 (3.72-4.66) | |
|  |  |  |  |  |  |  |  |  |  |  |  |  | |
| NTS (22) | 1987 | [52] | M | 18-34 | /R | A/R | 158 | 79 | 89 | 111 | (25.4%) | 2.49 (1.69-3.68) | |
|  |  |  |  | 35-70 |  |  | 223 | 8 | 363 | 111 | (15.7%) | 8.52 (4.08-17.8) | |
|  |  |  |  | Total 18-70 |  |  | 381 | 87 | 452 | 222 | (19.4%) | 2.15 (1.62-2.85) | |
|  |  |  |  |  |  |  |  |  |  |  |  |  | |
| Your country and your life (23) | 2001-02 | [53] | M | 16-79 | /R | A/R | 1397 | 402 | 888 | 438 | (14.0%) | 1.71 (1.46-2.01) | |
|  |  |  |  |  |  |  |  |  |  |  |  |  | |
| Stockholm Public Health follow-up (26) ^e^ | 2007 | [60] | M | 23-89 | /R | U/R | 3877 | 801 | 2610 | 2666 | (26.8%) | 4.94 (4.50-5.43) | |
|  |  |  |  |  |  |  |  |  |  |  |  |  | |
| Göteborg Public Dental Service (27) ^e^ | 1986 | [61] | M+F | 18-19 | /W | C/R | 517 | 110 | 209 | – |  | – | |
|  |  |  |  |  |  |  |  |  |  |  |  |  | |
| Stockholm family diabetes (29) | 1992-94 | [63] | M | 35-56 | /R | C/R | 895 | 177 | 1020 | 691 | (24.8%) | 3.43 (2.84-4.13) | |
|  |  |  |  |  |  |  |  |  |  |  |  |  | |
| AIR (33) | unkn | [67] | M | 58 | /R | C/R | 139 | 4 | 171 | 77 | (19.7%) | 15.65 (5.59-43.82) | |
|  |  |  |  |  |  |  |  |  |  |  |  |  | |
| Stockholm county 60 year olds (34) ^e^ | 1997-99 | [68] | M | 60 | /W | U/R | 594 | 28 | 1097 | 140 | (7.5%) | 2.71 (1.78-4.11) | |
|  |  |  |  |  |  |  |  |  |  |  |  |  | |
| Stockholm 31-40 year olds (36) | 1985? | [70] | M | 31-40 | /U | U/U | 250 | 51 | 432 | 90 | (10.9%) | 1.02 (0.70-1.49) | |
| ^a^ For case-control (CC) studies the data shown are for controls. The numbers in brackets refer to the text section numbers within §3.2  ^b^ Frequency of snus use : /R regular or daily, /W weekly, /A any (regular or occasional, /U unspecified  ^c^ First code show product smoked : C cigarettes, A any (cigarettes, pipe or cigar), U unspecified. Second code shows frequency of smoking, as in footnote b.  ^d^ No age restriction, workers  ^e^ See text for problems, doubts or assumptions for this study  f Matched on age to cases, who were mean age 72 (males) and 69 (females)  Note: The MS CC study (§3.2.5) gives results where snus use is current vs never not ever vs never as follows: | | | | | | | | | | | | |  |
|  | 2005-08 | [22] | M+F | 16-70 | /R | C/R | 903 | 84 | 702 | 186 | (10.0%) | 3.74 (2.77-5.05) | |
|  | | | | | | | | | | | | |  |

## TABLE 2 Association of current (vs non-current) snus use with current (vs non-current) smoking

| Study^a^ | Year | Source | Sex | Age | Definitions | | Numbers of subjects | | | | | OR (95% CI) | |
| --- | --- | --- | --- | --- | --- | --- | --- | --- | --- | --- | --- | --- | --- |
|  |  |  |  |  |  |  | Neither | Snus  only | Smoking  only | Snus and  smoking | |  | |
|  |  |  |  |  | Snus^b^ | Smok^c^ |  |  |  |  |  |  |  |
|  |  |  |  |  |  |  |  |  |  |  |  |  | |
| Kalixanda (2) ^d^ | 1998-2001 | [12] | M+F | 20-34 | /U | C/U | 52 | 18 | 19 | 5 | (5.3%) | 0.76 (0.25-2.33) | |
|  |  |  |  | 35-49 |  |  | 178 | 27 | 56 | 5 | (1.9%) | 0.59 (0.22-1.60) | |
|  |  |  |  | 50-64 |  |  | 255 | 41 | 69 | 10 | (2.7%) | 0.90 (0.43-1.89) | |
|  |  |  |  | 65+ |  |  | 221 | 10 | 21 | 2 | (0.8%) | 2.10 (0.43-10.25) | |
|  |  |  |  | Total 20+ |  |  | 706 | 96 | 165 | 22 | (2.2%) | 0.98 (0.60-1.61) | |
|  |  |  |  |  |  |  |  |  |  |  |  |  | |
| ULF (3) | 1980-81 | [16] | M | 16-24 | /A | A/R | 620 | 215 | 208 | 79 | (7.0%) | 1.09 (0.81-1.48) | |
|  |  |  | M | 25-44 |  |  | 1181 | 847 | 303 | 164 | (6.6%) | 0.75 (0.61-0.93) | |
|  |  |  | M | 45-64 |  |  | 1037 | 574 | 106 | 73 | (4.1%) | 1.24 (0.91-1.70) | |
|  |  |  | M | 65-84 |  |  | 971 | 451 | 200 | 63 | (3.7%) | 0.68 (0.50-0.92) | |
|  |  |  | M | Total 16-84 |  |  | 3810 | 2086 | 817 | 378 | (5.3%) | 0.85 (0.74-0.97) | |
|  |  |  |  |  |  |  |  |  |  |  |  |  | |
|  | 1988-89 |  | M | 16-24 | /A | A/R | 607 | 87 | 229 | 79 | (7.9%) | 2.41 (1.72-3.39) | |
|  |  |  | M | 25-44 |  |  | 1097 | 483 | 405 | 174 | (8.1%) | 0.98 (0.79-1.20) | |
|  |  |  | M | 45-64 |  |  | 1011 | 439 | 147 | 68 | (4.1%) | 1.07 (0.79-1.46) | |
|  |  |  | M | 65-84 |  |  | 861 | 249 | 138 | 34 | (2.6%) | 0.85 (0.57-1.26) | |
|  |  |  | M | Total 16-84 |  |  | 3576 | 1257 | 920 | 356 | (5.8%) | 1.10 (0.96-1.26) | |
|  |  |  | F | 16-84 |  |  | 4715 | 1618 | 45 | 41 | (0.6%) | 2.68 (1.75-4.10) | |
|  |  |  |  |  |  |  |  |  |  |  |  |  | |
|  | 1996-97 |  | M | 16-24 | /A | A/R | 526 | 79 | 222 | 43 | (4.9%) | 1.28 (0.86-1.92) | |
|  |  |  | M | 25-44 |  |  | 1104 | 270 | 581 | 116 | (5.6%) | 0.82 (0.64-1.04) | |
|  |  |  | M | 45-64 |  |  | 1068 | 313 | 237 | 78 | (4.6%) | 1.12 (0.84-1.49) | |
|  |  |  | M | 65-84 |  |  | 756 | 129 | 75 | 26 | (2.6%) | 2.01 (1.24-3.27) | |
|  |  |  | M | Total 16-84 |  |  | 3453 | 790 | 1115 | 262 | (4.7%) | 1.03 (0.88-1.20) | |
|  |  |  | F | 16-84 |  |  | 4507 | 1308 | 79 | 46 | (0.8%) | 2.02 (1.40-2.92) | |
|  |  |  |  |  |  |  |  |  |  |  |  |  | |
|  | 2004 |  | M | 16-24 | /A | A/R | 224 | 19 | 104 | 20 | (5.4%) | 2.22 (1.13-4.33) | |
|  |  |  | M | 25-44 |  |  | 528 | 66 | 296 | 50 | (5.3%) | 1.33 (0.90-1.98) | |
|  |  |  | M | 45-64 |  |  | 527 | 167 | 181 | 41 | (4.5%) | 0.71 (0.49-1.04) | |
|  |  |  | M | 65-84 |  |  | 384 | 38 | 36 | 1 | (0.2%) | 0.22 (0.02-2.10) | |
|  |  |  | M | Total 16-84 |  |  | 1663 | 291 | 617 | 111 | (4.1%) | 1.03 (0.81-1.30) | |
|  |  |  | F | 16-84 |  |  | 2231 | 450 | 90 | 36 | (1.3%) | 1.99 (1.33-2.96) | |
|  |  |  |  |  |  |  |  |  |  |  |  |  | |
| ULF (3) *continued* | 2010 | [17] | M | 16-24 | /R | A/R | (71.7%) | (8.2%) | (17.8%) |  | (2.3%) | 1.13 | |
|  |  |  | M | 25-34 |  |  | (64.7%) | (9.5%) | (23.5%) |  | (2.3%) | 0.67 | |
|  |  |  | M | 35-44 |  |  | (67.9%) | (8.5%) | (21.6%) |  | (2.0%) | 0.74 | |
|  |  |  | M | 45-54 |  |  | (66.5%) | (11.6%) | (19.7%) |  | (2.2%) | 0.64 | |
|  |  |  | M | 55-64 |  |  | (65.1%) | (16.2%) | (16.8%) |  | (1.9%) | 0.45 | |
|  |  |  | M | 65-74 |  |  | (73.4%) | (14.2%) | (10.8%) |  | (1.6%) | 0.77 | |
|  |  |  | M | 75-84 |  |  | (87.9%) | (5.2%) | (6.1%) |  | (0.8%) | 2.22 | |
|  |  |  | M | 85+ |  |  | (95.1%) | (1.5%) | (3.4%) |  | (0.0%) | 0.00 | |
|  |  |  | M | Total 16+ |  |  | (70.0%) | (10.6%) | (17.5%) |  | (1.9%) | 0.72 | |
|  |  |  | F | 16+ |  |  | (82.7%) | (14.3%) | (2.6%) |  | (0.4%) | 0.89 | |
|  |  |  |  |  |  |  |  |  |  |  |  |  | |
| SALT (4) | 1998-2002 | [19] | M | 42-64 | /A | C/A | 11342 | 2382 | 2639 | 279 | (1.7%) | 0.50 (0.44-0.57) | |
|  |  |  |  |  |  |  |  |  |  |  |  |  | |
| 2 AMI CC (6) | 1992-94 | [23] | M | 45-70 | /U | C/U | 1156 | 122 | 472 | 60 | (3.3%) | 1.20 (0.87-1.67) | |
|  |  |  |  |  |  |  |  |  |  |  |  |  | |
| MONICA (7) | 1986 | [29] | M | 25-64 | /R | C/R | 476 | 145 | 153 | 32 | (4.0%) | 0.69 (0.45-1.05) | |
|  |  | [26] | M | 25-64 | /U | A/A | 405 | 110 | 226 | 68 | (8.4%) | 1.11 (0.79-1.56) | |
|  |  |  |  |  |  |  |  |  |  |  |  |  | |
|  | 1990 | [29] | M | 25-64 | /R | C/R | 452 | 138 | 138 | 38 | (5.0%) | 0.90 (0.60-1.35) | |
|  |  |  | F | 25-64 | /R | C/R | 572 | 16 | 199 | 8 | (1.0%) | 1.44 (0.61-3.41) | |
|  |  |  |  |  |  |  |  |  |  |  |  |  | |
|  | 1994 | [29] | M | 25-74 | /R | C/R | 455 | 149 | 112 | 30 | (4.0%) | 0.82 (0.53-1.27) | |
|  |  |  | F | 25-74 | /R | C/R | 557 | 16 | 204 | 8 | (1.0%) | 1.37 (0.58-3.24) | |
|  |  |  |  |  |  |  |  |  |  |  |  |  | |
|  | 1999 | [29] | M | 25-74 | /R | C/R | 399 | 183 | 74 | 20 | (3.0%) | 0.59 (0.35-1.00) | |
|  |  |  | F | 25-74 | /R | C/R | 532 | 44 | 155 | 7 | (0.9%) | 0.55 (0.24-1.24) | |
|  |  |  |  |  |  |  |  |  |  |  |  |  | |
|  | 2004 | [30] | M | 25-34 | /R | C/R | 116 | 64 | 6 | 2 | (1.1%) | 0.60 (0.12-3.08) | |
|  |  |  |  | 35-44 |  |  | 103 | 64 | 13 | 7 | (3.7%) | 0.87 (0.33-2.29) | |
|  |  |  |  | 45-54 |  |  | 120 | 45 | 17 | 6 | (3.2%) | 0.94 (0.35-2.54) | |
|  |  |  |  | 55-64 |  |  | 122 | 34 | 28 | 4 | (2.1%) | 0.51 (0.17-1.56) | |
|  |  |  |  | 65-74 |  |  | 144 | 30 | 11 | 2 | (1.1%) | 0.87 (0.18-4.14) | |
|  |  |  |  | Total 25-74 |  |  | 580 | 252 | 84 | 19 | (2.0%) | 0.52 (0.31-0.88) | |
|  |  |  |  |  |  |  |  |  |  |  |  |  | |
|  |  |  | F | 25-74 | /R | C/R | 682 | 84 | 149 | 19 | (2.0%) | 1.04 (0.61-1.76) | |
|  |  |  |  |  |  |  |  |  |  |  |  |  | |
| Malmö diet (9) | 1991-96 | [33] | M | 45-73 | /U | U/A | 6960 | 487 | 2776 | 250 | (2.4%) | 1.29 (1.10-1.51) | |
|  |  |  | F |  |  |  | 12003 | 54 | 4676 | 21 | (0.1%) | 1.00 (0.60-1.65) | |
|  |  |  |  |  |  |  |  |  |  |  |  |  | |
| Oral cancer CC (14) | 1980-89 | [37] | M+F | ^e^ | /R | A/R | 228 | 38 | 72 | 16 | (4.5%) | 1.33 (0.70-2.53) | |
| VIP (15) | 1990-95 | [40] | M | 40 | /U | U/A | 2628 | 965 | 975 | 403 | (8.1%) | 1.13 (0.98-1.29) | |
|  |  |  | M | 50 |  |  | 2821 | 571 | 965 | 278 | (6.0%) | 1.42 (1.21-1.67) | |
|  |  |  | M | 60 |  |  | 2316 | 378 | 584 | 91 | (2.7%) | 0.95 (0.75-1.22) | |
|  |  |  | F | 40 |  |  | 3674 | 112 | 1742 | 67 | (1.2%) | 1.26 (0.93-1.72) | |
|  |  |  | F | 50 |  |  | 3760 | 26 | 1447 | 16 | (0.3%) | 1.60 (0.86-2.99) | |
|  |  |  | F | 60 |  |  | 3180 | 8 | 724 | 4 | (0.1%) | 2.20 (0.66-7.31) | |
|  |  |  |  |  |  |  |  |  |  |  |  |  | |
|  | 1996-2001 |  | M | 40 |  |  | 3013 | 1357 | 639 | 319 | (6.0%) | 1.11 (0.96-1.29) | |
|  |  |  | M | 50 |  |  | 3598 | 1222 | 999 | 391 | (6.3%) | 1.15 (1.01-1.32) | |
|  |  |  | M | 60 |  |  | 3110 | 628 | 637 | 145 | (3.2%) | 1.13 (0.92-1.38) | |
|  |  |  | F | 40 |  |  | 4037 | 381 | 1258 | 110 | (1.9%) | 0.93 (0.74-1.16) | |
|  |  |  | F | 50 |  |  | 4646 | 155 | 1583 | 51 | (0.8%) | 0.97 (0.70-1.33) | |
|  |  |  | F | 60 |  |  | 4032 | 35 | 900 | 5 | (0.1%) | 0.64 (0.25-1.64) | |
|  |  |  |  |  |  |  |  |  |  |  |  |  | |
|  | 2002-2007 |  | M | 40 |  |  | 3578 | 1702 | 430 | 339 | (5.6%) | 1.66 (1.42-1.93) | |
|  |  |  | M | 50 |  |  | 3739 | 1511 | 717 | 375 | (5.9%) | 1.29 (1.13-1.49) | |
|  |  |  | M | 60 |  |  | 4233 | 1122 | 840 | 224 | (3.5%) | 1.01 (0.86-1.18) | |
|  |  |  | F | 40 |  |  | 4652 | 742 | 767 | 132 | (2.1%) | 1.08 (0.88-1.32) | |
|  |  |  | F | 50 |  |  | 4842 | 415 | 1300 | 134 | (2.0%) | 1.20 (0.98-1.48) | |
|  |  |  | F | 60 |  |  | 5202 | 166 | 1203 | 40 | (0.6%) | 1.04 (0.73-1.48) | |
|  |  |  |  |  |  |  |  |  |  |  |  |  | |
| Birth register (16) | 1999-2006 | [42] | F | ^f^ | /U | C/R | 504531 | 7629 | 58502 | 483 | (0.1%) | 0.55 (0.50-0.60) | |
|  |  |  |  |  |  |  |  |  |  |  |  |  | |
| Eurobarometer (18) ^d^ | 2002 | [45] | M | 15+ | /U^g^ | A/U | 327 | 68 | 77 | 18 | (3.6%) | 1.12 (0.63-2.00) | |
|  |  |  |  |  |  |  |  |  |  |  |  |  | |
| Northern town 21-year olds (21) | 1986 | [49] | M | 21 | /U | C/A | 298 | 118 | 111 | 33 | (5.9%) | 0.75 (0.48-1.17) | |
|  | 1994 |  | M | 21 |  |  | 262 | 83 | 41 | 35 | (8.3%) | 2.69 (1.61-4.51) | |
|  |  |  |  |  |  |  |  |  |  |  |  |  | |
| NTS (22) | 1985 | [50] | M | 18-34 | /A | A/A | 164 | 79 | 92 | 82 | (19.7%) | 1.85 (1.24-2.76) | |
|  |  |  | M | 35-70 |  |  | 329 | 62 | 207 | 50 | (7.7%) | 1.28 (0.85-1.93) | |
|  |  |  | M | Total 18-70 |  |  | 493 | 141 | 299 | 132 | (12.4%) | 1.54 (1.17-2.04) | |
|  |  |  |  |  |  |  |  |  |  |  |  |  | |
|  | 1986 | [51] | M | 18-34 |  |  | 135 | 78 | 96 | 58 | (15.8%) | 1.05 (0.68-1.61) | |
|  |  |  | M | 35-70 |  |  | 343 | 76 | 185 | 46 | (7.1%) | 1.12 (0.75-1.69) | |
|  |  |  | M | Total 18-70 |  |  | 478 | 154 | 281 | 104 | (10.2%) | 1.15 (0.86-1.53) | |
|  |  |  |  |  |  |  |  |  |  |  |  |  | |
|  | 1987 | [52] | M | 18-34 |  |  | 193 | 87 | 81 | 86 | (19.2%) | 2.36 (1.59-3.50) | |
|  |  |  | M | 35-70 |  |  | 406 | 33 | 219 | 47 | (6.7%) | 2.64 (1.64-4.24) | |
|  |  |  | M | Total 18-70 |  |  | 599 | 120 | 300 | 133 | (11.5%) | 2.21 (1.67-2.94) | |
| Your country and your life (23) | 2001-02 | [53] | M | 16-24 | /R | A/R | 269 | 83 | 32 | 12 | (3.0%) | 1.22 (0.60-2.47) | |
|  |  |  | M | 25-44 |  |  | 648 | 319 | 121 | 22 | (2.0%) | 0.37 (0.23-0.59) | |
|  |  |  | M | 45-64 |  |  | 755 | 200 | 200 | 24 | (2.0%) | 0.45 (0.29-0.71) | |
|  |  |  | M | 65-79 |  |  | 451 | 39 | 68 | 6 | (1.1%) | 1.02 (0.42-2.50) | |
|  |  |  | M | Total 18-70 |  |  | 2105 | 648 | 421 | 65 | (2.0%) | 0.50 (0.38-0.66) | |
|  |  |  |  |  |  |  |  |  |  |  |  |  | |
| Health on equal terms survey (24) | 2004 | [54] | M | 16-29 | /R | U/R | 591 | 191 | 61 | 26 | (3.0%) | 1.32 (0.81-2.15) | |
|  |  |  | M | 30-44 |  |  | 837 | 363 | 153 | 42 | (3.0%) | 0.63 (0.44-0.91) | |
|  |  |  | M | 45-64 |  |  | 1252 | 358 | 318 | 60 | (3.0%) | 0.66 (0.49-0.89) | |
|  |  |  | M | 65-84 |  |  | 982 | 84 | 120 | 12 | (1.0%) | 1.17 (0.62-2.20) | |
|  |  |  | M | Total 18-70 |  |  | 3650 | 1035 | 599 | 163 | (3.0%) | 0.96 (0.80-1.16) | |
|  |  |  |  |  |  |  |  |  |  |  |  |  | |
|  | 2009 |  | M | 16-29 |  |  | 484 | 130 | 48 | 20 | (2.9%) | 1.55 (0.89-2.71) | |
|  |  |  | M | 30-44 |  |  | 660 | 183 | 64 | 9 | (1.0%) | 0.51 (0.25-1.04) | |
|  |  |  | M | 45-64 |  |  | 1125 | 285 | 218 | 50 | (3.0%) | 0.91 (0.65-1.26) | |
|  |  |  | M | 65-84 |  |  | 1025 | 114 | 114 | 13 | (1.0%) | 1.03 (0.56-1.88) | |
|  |  |  | M | Total 18-70 |  |  | 3272 | 772 | 409 | 91 | (2.0%) | 0.94 (0.74-1.20) | |
|  |  |  |  |  |  |  |  |  |  |  |  |  | |
| Stockholm Public Health baseline (26) | 2006 | [59] | M | 18-84 | /R | U/R | 10690 | 2624 | 1745 | 369 | (2.4%) | 0.86 (0.76-0.97) | |
|  |  |  | F | 18-84 |  |  | 15237 | 576 | 2853 | 95 | (0.5%) | 0.88 (0.71-1.10) | |
|  |  |  |  |  |  |  |  |  |  |  |  |  | |
| Skåne PublicHealth (28) | 2004 | [62] | M | 18-80 | /R | U/A | 7730 | 1861 | 1813 | 451 | (3.8%) | 1.03 (0.92-1.16) | |
|  |  |  | F | 18-80 |  |  | 10665 | 251 | 3061 | 73 | (0.5%) | 1.01 (0.78-1.32) | |
|  |  |  |  |  |  |  |  |  |  |  |  |  | |
| Värmland ice hockey players (30) | 2001 | [64] | M | 18-22 | /A | U/U | 56 | 27 | 2 | 10 | (10.5%) | 10.37 (2.12-50.66) | |
|  |  |  |  |  |  |  |  |  |  |  |  |  | |
| Älvsborg county (31) | 1983-84 | [65] | M | 20+ | /U | A/R | 232 | 58 | 137 | 21 | (4.7%) | 0.61 (0.36-1.05) | |
|  |  |  |  |  |  |  |  |  |  |  |  |  | |
| AIR (33) | unkn | [67] | M | 58 | /R | C/R | 261 | 34 | 82 | 14 | (3.6%) | 1.31 (0.67-2.56) | |
|  |  |  |  |  |  |  |  |  |  |  |  |  | |
| Stockholm county 60 year olds (34) ^d^ | 1997-99 | [68] | M | 60 | /W | U/R | 1343 | 129 | 360 | 27 | (1.5%) | 0.78 (0.51-1.20) | |
|  |  |  |  |  |  |  |  |  |  |  |  |  | |
| Säve military conscripts (35) | unkn | [69] | M | 18-19 | /R | A/R | 377 | 127 | 43 | 30 | (5.2%) | 2.07 (1.25-3.44) | |
|  |  |  |  |  |  |  |  |  |  |  |  |  | |
| Stockholm 31-40 year olds (36) | 1985? | [70] | M | 31-40 | /U | U/U | 434 | 75 | 278 | 36 | (4.4%) | 0.75 (0.49-1.15) | |
| ^a^ For case-control (CC) studies the data shown are for controls. The numbers in brackets refer to the text section number within §3.2  ^b^ Frequency of snus use : /R regular or daily, /W weekly, /A any (regular or occasional, /U unspecified  ^c^ First code show product smoked : C cigarettes, A any (cigarettes, pipe or cigar), U unspecified. Second code shows frequency of smoking, as in footnote b.  ^d^ See text for problems, doubts or assumptions for this study  ^e^ Matched on age to cases, who were mean age 72 (males and 69 (females)  ^f^ Age range not stated, but of child-bearing age  ^g^ Chew tobacco or take snuff | | | | | | | | | | | | |  |

## TABLE 3 Daily consumption^a^ of tobacco in single and dual users

| Study^b^ | Year | Source | Sex | Age | Snus only | Smoking only | Snus and Smoking | |
| --- | --- | --- | --- | --- | --- | --- | --- | --- |
|  |  |  |  |  | (grams) | (cigs) | (grams) | (cigs) |
|  |  |  |  |  |  |  |  |  |
| Construction workers (1) | 1978-93 | [11] | M | <24 | 23 | 10 | 19 (83%) | 8 (80%) |
|  |  |  |  | 25-34 | 23 | 12 | 17 (74%) | 10 (83%) |
|  |  |  |  | 35-44 | 21 | 13 | 16 (76%) | 11 (85%) |
|  |  |  |  | 45-54 | 19 | 11 | 14 (74%) | 10 (91%) |
|  |  |  |  | 55+ | 15 | 10 | 12 (80%) | 8 (80%) |
|  |  |  |  | Total ^c^ | 22 | 12 | 16 (73%) | 9 (75%) |
|  |  |  |  |  |  |  |  |  |
| Kalixanda (2) | 1998-2001 | [12] | M+F | 20+ | 3.2^d^ | 11.5 | 2.2^d^ (69%) | 6.2 (54%) |
|  |  |  |  |  |  |  |  |  |
| SALT (4)^e^ | 1998-2002 | [19] | M | 20+ | - | 16.7 | - | 16.5 (98%) |
|  |  |  |  |  |  |  |  |  |
| 2 AMI CC (6)^f^ | 1992-94 | [23] | M | 45-70 | - | 18.6 | - | 16.4 (88%) |
|  |  |  |  |  |  |  |  |  |
| MONICA (7) ^g^ | 1986-1999 | [29] | M | 25-74 | 0.42^h^ | 15.8 | 0.25^h^ (59%) | 10.8 (68%) |
|  |  |  |  |  |  |  |  |  |
|  | 1990 | [31] | M | 25-64 | 3.2^d^ | 16.5 | 2.5^d^ (78%) | 10.1 (61%) |
|  |  |  |  |  |  |  |  |  |
| Malmö diet (9) | 1991-96 | [33] | M | 45-73 | - | 16.1 | - | 12.3 (76%) |
|  |  | [33] | F |  | - | 12.9 | - | 7.8 (60%) |
|  |  |  |  |  |  |  |  |  |
| Telephone smokers survey (20) | 2000 | [48] | M | 25-55 | - | 15.1 | - | 11.0 (73%) |
|  |  |  | F |  | - | 12.3 | - | 11.7 (95%) |
|  |  |  |  |  |  |  |  |  |
| Säve military conscripts (35) ^i^ | unknown | [69] | M | 18-19 | 25 | 12.2 | 27 (108%) | 7.8 (64%) |
| ^a^ Means  ^b^ For case-control (CC) studies the data shown are for controls. The numbers in brackets refer to the text section number within §3.2  ^c^ No age restriction, workers  ^d^ Cans/week  ^e^ Estimated from distribution given. Cigarette consumption is by current or former smokers. Dual users are current snus users.  ^f^ Data are for current smokers. Consumption by former smokers is 20.6 cigs/day for smoking only and 18.4 (90%) for dual users of snus and tobacco  ^g^ See text-table in §3.2.7 for further results on total tobacco consumption and biomarkers  ^h^ Packages per day  ^i^ See text table in §3.2.35 for further results on biomarkers  Note: Related results are given in the text for the following studies – NTS (22) distribution of current heavy smoking , Vara (32) snus consumption by current/former smoking, and results from studies 5, 14, 33 and 35 on lifetime consumption | | | | | | | | |

## TABLE 4 Association of current/ex/never snus use with current/ex/never smoking – studies giving at least 6 categories

### TABLE 4A Numbers of subjects

| Study^a^ | Year | Source | Sex | Age | Definitions | | Current Smoking | | |  | Ex smoking | | |  | Never smoking | | |
| --- | --- | --- | --- | --- | --- | --- | --- | --- | --- | --- | --- | --- | --- | --- | --- | --- | --- |
|  |  |  |  |  | Snus^b^ | Smok^c^ | Current snus | Ex  snus | Never snus |  | Current snus | Ex  snus | Never snus |  | Current snus | Ex  snus | Never snus |
|  |  |  |  |  |  |  |  |  |  |  |  |  |  |  |  |  |  |
| Kalixanda (2) | 1998-2001 | [12] | M+F | 20-34 | /U | C/U | 5 | ^d^ | 19 |  | ^d^ | 5 | 9 |  | 18 | 2 | 36 |
|  |  |  |  | 35-49 |  |  | 5 | ^d^ | 56 |  | ^d^ | 12 | 48 |  | 27 | 6 | 112 |
|  |  |  |  | 50-64 |  |  | 10 | ^d^ | 69 |  | ^d^ | 19 | 85 |  | 41 | 4 | 147 |
|  |  |  |  | >65 |  |  | 2 | ^d^ | 21 |  | ^d^ | 13 | 67 |  | 10 | 4 | 137 |
|  |  |  |  | 20+ |  |  | 22 | ^d^ | 165 |  | ^d^ | 49 | 209 |  | 96 | 16 | 432 |
|  |  |  |  |  |  |  |  |  |  |  |  |  |  |  |  |  |  |
| ULF (3) | 1980-81 | [16] | M | 16-24 | /A | A/R | 79 | 215 | ← |  | 72 | 80 | ← |  | 136 | 541 | ← |
|  |  |  | M | 25-44 |  |  | 164 | 847 | ← |  | 203 | 486 | ← |  | 99 | 695 | ← |
|  |  |  | M | 45-64 |  |  | 73 | 574 | ← |  | 80 | 528 | ← |  | 26 | 510 | ← |
|  |  |  | M | 65-84 |  |  | 63 | 451 | ← |  | 131 | 574 | ← |  | 69 | 397 | ← |
|  |  |  | M | 16-84 |  |  | 378 | 2086 | ← |  | 487 | 1667 | ← |  | 330 | 2143 | ← |
|  |  |  |  |  |  |  |  |  |  |  |  |  |  |  |  |  |  |
|  | 1988-89 |  | M | 16-24 | /A | A/R | 79 | 87 | ← |  | 63 | 37 | ← |  | 167 | 570 | ← |
|  |  |  | M | 25-44 |  |  | 174 | 483 | ← |  | 235 | 372 | ← |  | 170 | 725 | ← |
|  |  |  | M | 45-64 |  |  | 68 | 439 | ← |  | 114 | 505 | ← |  | 34 | 506 | ← |
|  |  |  | M | 65-84 |  |  | 34 | 249 | ← |  | 101 | 496 | ← |  | 37 | 365 | ← |
|  |  |  | M | 16-84 |  |  | 356 | 1257 | ← |  | 512 | 1410 | ← |  | 408 | 2166 | ← |
|  |  |  | F | 16-84 |  |  | 41 | 1618 | ← |  | 26 | 1172 | ← |  | 19 | 3543 | ← |
|  |  |  |  |  |  |  |  |  |  |  |  |  |  |  |  |  |  |
|  | 1996-97 |  | M | 16-24 | /A | A/R | 43 | 79 | ← |  | 85 | 35 | ← |  | 137 | 490 | ← |
|  |  |  | M | 25-44 |  |  | 116 | 270 | ← |  | 290 | 277 | ← |  | 291 | 826 | ← |
|  |  |  | M | 45-64 |  |  | 78 | 313 | ← |  | 184 | 569 | ← |  | 53 | 499 | ← |
|  |  |  | M | 65-84 |  |  | 26 | 129 | ← |  | 62 | 437 | ← |  | 14 | 318 | ← |
|  |  |  | M | 16-84 |  |  | 262 | 790 | ← |  | 620 | 1319 | ← |  | 495 | 2134 | ← |
|  |  |  | F | 16-84 |  |  | 46 | 1308 | ← |  | 51 | 1370 | ← |  | 28 | 3137 | ← |
|  |  |  |  |  |  |  |  |  |  |  |  |  |  |  |  |  |  |
|  | 2004 |  | M | 16-24 | /A | A/R | 20 | 19 | ← |  | 46 | 15 | ← |  | 58 | 209 | ← |
|  |  |  | M | 25-44 |  |  | 50 | 66 | ← |  | 141 | 96 | ← |  | 155 | 432 | ← |
|  |  |  | M | 45-64 |  |  | 41 | 167 | ← |  | 140 | 244 | ← |  | 41 | 283 | ← |
|  |  |  | M | 65-84 |  |  | 1 | 38 | ← |  | 32 | 223 | ← |  | 3 | 160 | ← |
|  |  |  | M | 16-84 |  |  | 111 | 291 | ← |  | 359 | 578 | ← |  | 258 | 1084 | ← |
|  |  |  | F | 16-84 |  |  | 36 | 450 | ← |  | 69 | 695 | ← |  | 21 | 1536 | ← |
|  |  |  |  |  |  |  |  |  |  |  |  |  |  |  |  |  |  |
| SALT (4) | 1998-2002 | [19] | M | 42-64 | /A | C/A | 279 | 159 | 2480 |  | 1368 | 802 | 3720 |  | 1014 | 495 | 6325 |
|  |  |  |  |  |  |  |  |  |  |  |  |  |  |  |  |  |  |
|  |  |  |  |  |  |  |  |  |  |  |  |  |  |  |  |  |  |
| 2 AMI CC (6) | 1992-94 | [23] | M | 45-70 | /U | C/U | 60 | 12 | 460 |  | 94 | 66 | 480 |  | 28 | 12 | 598 |
|  |  |  |  |  |  |  |  |  |  |  |  |  |  |  |  |  |  |
| MONICA (7) | 1986 | [29] | M | 25-64 | /R | C/R | 32 | 32 | 121 |  | 73 | 54 | 115 |  | 73 | 24 | 282 |
|  |  | [26] | M | 25-64 | /U | A/A | 68 | 80 | 30 |  | 51 | 57 | 4 |  | 175 | 136 | 208 |
|  |  |  |  |  |  |  |  |  |  |  |  |  |  |  |  |  |  |
|  | 1990 | [29] | M | 25-64 | /R | C/R | 38 | 23 | 115 |  | 61 | 54 | 92 |  | 77 | 54 | 253 |
|  |  |  | F | 25-64 | /R | C/R | 8 | 0 | 199 |  | 8 | 8 | 120 |  | 8 | 8 | 437 |
|  |  |  |  |  |  |  |  |  |  |  |  |  |  |  |  |  |  |
|  | 1994 | [29] | M | 25-74 | /R | C/R | 30 | 15 | 97 |  | 90 | 50 | 99 |  | 60 | 37 | 269 |
|  |  |  | F | 25-74 | /R | C/R | 8 | 0 | 204 |  | 8 | 17 | 179 |  | 8 | 8 | 353 |
|  |  |  |  |  |  |  |  |  |  |  |  |  |  |  |  |  |  |
|  | 1999 | [29] | M | 25-74 | /R | C/R | 20 | 27 | 47 |  | 95 | 52 | 76 |  | 88 | 34 | 237 |
|  |  |  | F | 25-74 | /R | C/R | 7 | 7 | 148 |  | 30 | 11 | 159 |  | 15 | 0 | 362 |
|  |  |  |  |  |  |  |  |  |  |  |  |  |  |  |  |  |  |
| Malmö diet (9) | 1991-96 | [33] | M | 45-73 | /U | U/A | 250 | 2776 | ← |  | 420 | 4014 | ← |  | 67 | 2946 | ← |
|  |  |  | F | 45-73 | /U | U/A | 21 | 4676 | ← |  | 27 | 4599 | ← |  | 27 | 7404 | ← |
|  |  |  |  |  |  |  |  |  |  |  |  |  |  |  |  |  |  |
| IBD CC Stockholm (11) | 1984-87 | [34] | M | 15-79 | /R | C/U | 5 | ← | 46 |  | 5 | ← | 17 |  | 11 | ← | 61 |
|  |  |  |  |  |  |  |  |  |  |  |  |  |  |  |  |  |  |
| Oral cancer CC (14) | 1980-89 | [37] | M+F | ^e^ | /R | A/R | 16 | 1 | 71 |  | 15 | 13 | 67 |  | 23 | 4 | 144 |
|  |  |  |  |  |  |  |  |  |  |  |  |  |  |  |  |  |  |
| VIP (15) | 1990-95 | [40] | M | 40 | /U | U/A | 403 | 318 | 657 |  | 642 | 448 | 473 |  | 323 | 194 | 1513 |
|  |  |  | M | 50 |  |  | 278 | 190 | 775 |  | 436 | 422 | 812 |  | 135 | 116 | 1471 |
|  |  |  | M | 60 |  |  | 91 | 88 | 496 |  | 297 | 256 | 772 |  | 81 | 44 | 1244 |
|  |  |  | F | 40 |  |  | 67 | 73 | 1669 |  | 95 | 90 | 1355 |  | 17 | 17 | 2212 |
|  |  |  | F | 50 |  |  | 16 | 10 | 1437 |  | 21 | 21 | 1311 |  | 5 | 5 | 2423 |
|  |  |  | F | 60 |  |  | 4 | 4 | 720 |  | 8 | 4 | 837 |  | 0 | 0 | 2339 |
|  |  |  |  |  |  |  |  |  |  |  |  |  |  |  |  |  |  |
|  | 1996-2001 |  | M | 40 |  |  | 319 | 213 | 426 |  | 697 | 357 | 373 |  | 660 | 271 | 2012 |
|  |  |  | M | 50 |  |  | 391 | 248 | 751 |  | 912 | 676 | 856 |  | 310 | 211 | 1855 |
|  |  |  | M | 60 |  |  | 145 | 113 | 524 |  | 479 | 429 | 945 |  | 149 | 104 | 1632 |
|  |  |  | F | 40 |  |  | 110 | 121 | 1137 |  | 289 | 214 | 1328 |  | 92 | 64 | 2431 |
|  |  |  | F | 50 |  |  | 51 | 32 | 1551 |  | 142 | 58 | 1847 |  | 13 | 6 | 2735 |
|  |  |  | F | 60 |  |  | 5 | 5 | 895 |  | 35 | 15 | 1332 |  | 0 | 0 | 2685 |
|  |  |  |  |  |  |  |  |  |  |  |  |  |  |  |  |  |  |
|  |  |  |  |  |  |  |  |  |  |  |  |  |  |  |  |  |  |
|  |  |  |  |  |  |  |  |  |  |  |  |  |  |  |  |  |  |
| VIP (15) *continued* | 2002-2007 |  | M | 40 |  |  | 339 | 145 | 285 |  | 745 | 260 | 242 |  | 957 | 400 | 2676 |
|  |  |  | M | 50 |  |  | 375 | 203 | 514 |  | 971 | 622 | 597 |  | 540 | 362 | 2158 |
|  |  |  | M | 60 |  |  | 224 | 167 | 673 |  | 891 | 782 | 1238 |  | 231 | 186 | 2027 |
|  |  |  | F | 40 |  |  | 132 | 126 | 641 |  | 497 | 245 | 943 |  | 245 | 145 | 3319 |
|  |  |  | F | 50 |  |  | 134 | 74 | 1226 |  | 375 | 188 | 1855 |  | 40 | 33 | 2766 |
|  |  |  | F | 60 |  |  | 40 | 20 | 1183 |  | 159 | 53 | 2148 |  | 7 | 7 | 2994 |
|  |  |  |  |  |  |  |  |  |  |  |  |  |  |  |  |  |  |
| Gastric cancer CC (17) | 1989-95 | [44] | M | 40-79 | /W | A/R | 42 | ← | 135 |  | 114 | ← | 235 |  | 36 | ← | 217 |
|  |  |  |  |  |  |  |  |  |  |  |  |  |  |  |  |  |  |
| NTS (22) | 1985 | [50] | M | 18-34 | /R | A/R | 17 | 90 | ← |  | 64 | 48 | ← |  | 47 | 151 | ← |
|  |  |  | M | 35-70 |  |  | 12 | 196 | ← |  | 40 | 157 | ← |  | 28 | 210 | ← |
|  |  |  | M | 18-70 |  |  | 29 | 286 | ← |  | 104 | 205 | ← |  | 75 | 361 | ← |
|  |  |  |  |  |  |  |  |  |  |  |  |  |  |  |  |  |  |
|  | 1986 | [51] | M | 18-34 | /R | A/R | 7 | 87 | ← |  | 29 | 36 | ← |  | 71 | 139 | ← |
|  |  |  | M | 35-70 |  |  | 9 | 168 | ← |  | 73 | 182 | ← |  | 11 | 211 | ← |
|  |  |  | M | 18-70 |  |  | 16 | 255 | ← |  | 102 | 218 | ← |  | 82 | 350 | ← |
|  |  |  |  |  |  |  |  |  |  |  |  |  |  |  |  |  |  |
|  | 1987 | [52] | M | 18-34 | /R | A/R | 24 | 25 | 51 |  | 48 | 14 | 38 |  | 70 | 9 | 158 |
|  |  |  | M | 35-70 |  |  | 21 | 28 | 164 |  | 27 | 35 | 199 |  | 6 | 2 | 223 |
|  |  |  | M | 18-70 |  |  | 45 | 53 | 215 |  | 75 | 49 | 237 |  | 76 | 11 | 381 |
|  |  |  |  |  |  |  |  |  |  |  |  |  |  |  |  |  |  |
| Your country and your life (23) | 2001-02 | [53] | M | 16-79 | /R | A/R | 47 | 20 | 391 |  | 263 | 108 | 497 |  | 310 | 92 | 1397 |
|  |  |  |  |  |  |  |  |  |  |  |  |  |  |  |  |  |  |
| Stockholm Fire Brigade (25) | 1993 | [55] | M | 35-60 | ^f^ | U/R | 5 | 2 | 26 |  | 21 | 26 | ← |  | 29 | ←* | 42 |
|  |  |  |  |  |  |  |  |  |  |  |  |  |  |  |  |  |  |
| Stockholm Public Health follow-up (26) | 2007 | [60] | M | 23-89 | /R | U/R | 2666^g^ | ← | 785 |  | ←* | ←* | 1825 |  | 497 | 301 | 3877 |
|  |  |  |  |  |  |  |  |  |  |  |  |  |  |  |  |  |  |
|  |  |  |  |  |  |  |  |  |  |  |  |  |  |  |  |  |  |
|  |  |  |  |  |  |  |  |  |  |  |  |  |  |  |  |  |  |
|  |  |  |  |  |  |  |  |  |  |  |  |  |  |  |  |  |  |
| Skåne Public Health (28) | 2004 | [62] | M | 18-80 | /R | U/R | 451 | 1813 | ← |  | 925 | 2491 | ← |  | 936 | 5239 | ← |
|  |  |  | F | 18-80 |  |  | 73 | 3061 | ← |  | 173 | 2882 | ← |  | 78 | 7783 | ← |
|  |  |  |  |  |  |  |  |  |  |  |  |  |  |  |  |  |  |
| Stockholm family diabetes (29) | 1992-94 | [63] | M | 35-56 | /R | C/R | 691 | ← | 517 |  | ←* | ←* | 503 |  | 121 | 56 | 895 |
|  |  |  |  |  |  |  |  |  |  |  |  |  |  |  |  |  |  |
| Vara municipality (32) | 2001-03 | [66] | M | 30-75 | /W | A/R | excluded | 109 | ← |  | 116 | 189 | ← |  | 63 | 357 | ← |
|  |  |  |  |  |  |  |  |  |  |  |  |  |  |  |  |  |  |
| AIR (33) | unknown | [67] | M | 58 | /R | C/R | 14 | 12 | 70 |  | 32 | 19 | 101 |  | 2 | 2 | 139 |
|  |  |  |  |  |  |  |  |  |  |  |  |  |  |  |  |  |  |
| Stockholm county 60 year olds (34) | 1997-99 | [68] | M | 60 | /W | U/R | 27 | 0^h^ | 360 |  | 113 | 0^h^ | 737 |  | 16 | 12 | 594 |
| Footnotes – see end of Table 4B | | | | | | | | | | | | | | | | | |

### TABLE 4B Percentages

| Study^a^ | Year | Source | Sex | Age | Definitions | | Current Smoking | | |  | Ex smoking | | |  | Never smoking | | |
| --- | --- | --- | --- | --- | --- | --- | --- | --- | --- | --- | --- | --- | --- | --- | --- | --- | --- |
|  |  |  |  |  | Snus^b^ | Smok^c^ | Current snus | Ex  snus | Never snus |  | Current snus | Ex  snus | Never snus |  | Current snus | Ex  snus | Never snus |
|  |  |  |  |  |  |  |  |  |  |  |  |  |  |  |  |  |  |
| Kalixanda (2) | 1998-2001 | [12] | M+F | 20-34 | /U | C/U | 5.3 | ^d^ | 20.2 |  | ^d^ | 5.3 | 9.6 |  | 19.1 | 2.1 | 38.3 |
|  |  |  |  | 35-49 |  |  | 1.9 | ^d^ | 21.1 |  | ^d^ | 4.5 | 18.0 |  | 10.2 | 2.3 | 42.1 |
|  |  |  |  | 50-64 |  |  | 2.7 | ^d^ | 18.4 |  | ^d^ | 5.1 | 22.7 |  | 10.9 | 1.1 | 39.2 |
|  |  |  |  | >65 |  |  | 0.8 | ^d^ | 8.3 |  | ^d^ | 5.1 | 26.4 |  | 3.9 | 1.6 | 53.9 |
|  |  |  |  | 20+ |  |  | 2.2 | ^d^ | 16.7 |  | ^d^ | 5.0 | 21.1 |  | 9.7 | 1.6 | 43.7 |
|  |  |  |  |  |  |  |  |  |  |  |  |  |  |  |  |  |  |
| ULF (3) | 1980-81 | [16] | M | 16-24 | /A | A/R | 7.0 | 19.1 | ← |  | 6.4 | 7.1 | ← |  | 12.2 | 48.2 | ← |
|  |  |  | M | 25-44 |  |  | 6.6 | 34.0 | ← |  | 8.2 | 19.5 | ← |  | 4.0 | 27.9 | ← |
|  |  |  | M | 45-64 |  |  | 4.1 | 32.0 | ← |  | 4.5 | 29.5 | ← |  | 1.4 | 28.5 | ← |
|  |  |  | M | 65-84 |  |  | 3.7 | 26.8 | ← |  | 7.8 | 34.1 | ← |  | 4.1 | 23.6 | ← |
|  |  |  | M | 16-84 |  |  | 5.3 | 29.4 | ← |  | 6.9 | 23.5 | ← |  | 4.7 | 30.2 | ← |
|  |  |  |  |  |  |  |  |  |  |  |  |  |  |  |  |  |  |
|  | 1988-89 |  | M | 16-24 | /A | A/R | 7.9 | 8.7 | ← |  | 6.2 | 3.7 | ← |  | 16.6 | 56.8 | ← |
|  |  |  | M | 25-44 |  |  | 8.1 | 22.4 | ← |  | 10.9 | 17.2 | ← |  | 7.9 | 33.6 | ← |
|  |  |  | M | 45-64 |  |  | 4.1 | 26.4 | ← |  | 6.8 | 30.3 | ← |  | 2.0 | 30.4 | ← |
|  |  |  | M | 65-84 |  |  | 2.6 | 19.4 | ← |  | 7.9 | 38.7 | ← |  | 2.9 | 28.4 | ← |
|  |  |  | M | 16-84 |  |  | 5.8 | 20.6 | ← |  | 8.4 | 23.1 | ← |  | 6.7 | 35.5 | ← |
|  |  |  | F | 16-84 |  |  | 0.6 | 25.2 | ← |  | 0.4 | 18.3 | ← |  | 0.3 | 55.2 | ← |
|  |  |  |  |  |  |  |  |  |  |  |  |  |  |  |  |  |  |
|  | 1996-97 |  | M | 16-24 | /A | A/R | 4.9 | 9.0 | ← |  | 9.8 | 4.1 | ← |  | 15.8 | 56.4 | ← |
|  |  |  | M | 25-44 |  |  | 5.6 | 13.0 | ← |  | 14.0 | 13.4 | ← |  | 14.1 | 39.9 | ← |
|  |  |  | M | 45-64 |  |  | 4.6 | 18.5 | ← |  | 10.8 | 33.6 | ← |  | 3.2 | 29.4 | ← |
|  |  |  | M | 65-84 |  |  | 2.6 | 13.1 | ← |  | 6.3 | 44.4 | ← |  | 1.4 | 32.3 | ← |
|  |  |  | M | 16-84 |  |  | 4.7 | 14.1 | ← |  | 11.0 | 23.5 | ← |  | 8.8 | 38.0 | ← |
|  |  |  | F | 16-84 |  |  | 0.8 | 22.0 | ← |  | 0.9 | 23.1 |  |  | 0.5 | 52.8 |  |
|  |  |  |  |  |  |  |  |  |  |  |  |  |  |  |  |  |  |
|  | 2004 |  | M | 16-24 | /A | A/R | 5.4 | 5.2 | ← |  | 12.5 | 4.1 | ← |  | 15.9 | 56.9 | ← |
|  |  |  | M | 25-44 |  |  | 5.3 | 7.1 | ← |  | 15.0 | 10.2 | ← |  | 16.5 | 46.0 | ← |
|  |  |  | M | 45-64 |  |  | 4.5 | 18.2 | ← |  | 15.2 | 26.7 | ← |  | 4.5 | 30.9 | ← |
|  |  |  | M | 65-84 |  |  | 0.2 | 8.4 | ← |  | 7.1 | 48.7 | ← |  | 0.7 | 35.0 | ← |
|  |  |  | M | 16-84 |  |  | 4.1 | 10.8 | ← |  | 13.4 | 21.6 | ← |  | 9.6 | 40.4 | ← |
|  |  |  | F | 16-84 |  |  | 1.3 | 16.0 | ← |  | 2.5 | 24.7 | ← |  | 0.7 | 54.7 | ← |
|  |  |  |  |  |  |  |  |  |  |  |  |  |  |  |  |  |  |
| SALT (4) | 1998-2002 | [19] | M | 42-64 | /A | C/A | 1.7 | 1.0 | 14.9 |  | 8.2 | 4.8 | 22.4 |  | 6.1 | 3.0 | 38.0 |
|  |  |  |  |  |  |  |  |  |  |  |  |  |  |  |  |  |  |
|  |  |  |  |  |  |  |  |  |  |  |  |  |  |  |  |  |  |
|  |  |  |  |  |  |  |  |  |  |  |  |  |  |  |  |  |  |
|  |  |  |  |  |  |  |  |  |  |  |  |  |  |  |  |  |  |
| 2 AMI CC (6) | 1992-94 | [23] | M | 45-70 | /U | C/U | 3.3 | 0.7 | 25.4 |  | 5.2 | 3.6 | 26.5 |  | 1.5 | 0.7 | 33.0 |
|  |  |  |  |  |  |  |  |  |  |  |  |  |  |  |  |  |  |
| MONICA (7) | 1986 | [29] | M | 25-64 | /R | C/R | 4.0 | 4.0 | 15.0 |  | 9.0 | 6.7 | 14.3 |  | 9.0 | 3.0 | 35.0 |
|  |  | [26] | M | 25-64 | /U | A/A | 8.3 | 9.7 | 3.6 |  | 6.2 | 6.9 | 0.5 |  | 21.2 | 16.5 | 25.3 |
|  |  |  |  |  |  |  |  |  |  |  |  |  |  |  |  |  |  |
|  | 1990 | [29] | M | 25-64 | /R | C/R | 5.0 | 3.0 | 15.0 |  | 8.0 | 7.0 | 12.0 |  | 10.0 | 7.0 | 33.0 |
|  |  |  | F | 25-64 | /R | C/R | 1.0 | 0.0 | 25.0 |  | 1.0 | 0.9 | 15.1 |  | 1.0 | 1.0 | 55.0 |
|  |  |  |  |  |  |  |  |  |  |  |  |  |  |  |  |  |  |
|  | 1994 | [29] | M | 25-74 | /R | C/R | 4.0 | 2.0 | 13.0 |  | 12.0 | 6.7 | 13.3 |  | 8.0 | 5.0 | 36.0 |
|  |  |  | F | 25-74 | /R | C/R | 1.0 | 0.0 | 26.0 |  | 1.0 | 2.2 | 22.8 |  | 1.0 | 1.0 | 45.0 |
|  |  |  |  |  |  |  |  |  |  |  |  |  |  |  |  |  |  |
|  | 1999 | [29] | M | 25-74 | /R | C/R | 3.0 | 4.0 | 7.0 |  | 14.0 | 7.7 | 11.3 |  | 13.0 | 5.0 | 35.0 |
|  |  |  | F | 25-74 | /R | C/R | 1.0 | 1.0 | 20.0 |  | 4.0 | 1.4 | 21.6 |  | 2.0 | 0.0 | 49.0 |
|  |  |  |  |  |  |  |  |  |  |  |  |  |  |  |  |  |  |
| Malmö diet (9) | 1991-96 | [33] | M | 45-73 | /U | U/A | 2.4 | 26.5 | ← |  | 4.0 | 38.3 | ← |  | 0.6 | 28.1 | ← |
|  |  |  | F | 45-73 | /U | U/A | 0.1 | 27.9 | ← |  | 0.2 | 27.5 | ← |  | 0.2 | 44.2 | ← |
|  |  |  |  |  |  |  |  |  |  |  |  |  |  |  |  |  |  |
| IBD CC Stockholm (11) | 1984-87 | [34] | M | 15-79 | /R | C/U | 3.4 | ← | 31.7 |  | 3.4 | ← | 11.7 |  | 7.6 | ← | 42.1 |
|  |  |  |  |  |  |  |  |  |  |  |  |  |  |  |  |  |  |
| Oral cancer CC (14) | 1980-89 | [37] | M+F | ^e^ | /R | A/R | 4.5 | 0.3 | 20.1 |  | 4.2 | 3.7 | 18.9 |  | 6.5 | 1.1 | 40.7 |
|  |  |  |  |  |  |  |  |  |  |  |  |  |  |  |  |  |  |
| VIP (15) | 1990-95 | [40] | M | 40 | /U | U/A | 8.1 | 6.4 | 13.2 |  | 12.9 | 9.0 | 9.5 |  | 6.5 | 3.9 | 30.4 |
|  |  |  | M | 50 |  |  | 6.0 | 4.1 | 16.7 |  | 9.4 | 9.1 | 17.5 |  | 2.9 | 2.5 | 31.7 |
|  |  |  | M | 60 |  |  | 2.7 | 2.6 | 14.7 |  | 8.8 | 7.6 | 22.9 |  | 2.4 | 1.3 | 36.9 |
|  |  |  | F | 40 |  |  | 1.2 | 1.3 | 29.8 |  | 1.7 | 1.6 | 24.2 |  | 0.3 | 0.3 | 39.5 |
|  |  |  | F | 50 |  |  | 0.3 | 0.2 | 27.4 |  | 0.4 | 0.4 | 25.0 |  | 0.1 | 0.1 | 46.2 |
|  |  |  | F | 60 |  |  | 0.1 | 0.1 | 18.4 |  | 0.2 | 0.1 | 21.4 |  | 0.0 | 0.0 | 59.8 |
|  |  |  |  |  |  |  |  |  |  |  |  |  |  |  |  |  |  |
|  | 1996-2001 |  | M | 40 |  |  | 6.0 | 4.0 | 8.0 |  | 13.1 | 6.7 | 7.0 |  | 12.4 | 5.1 | 37.8 |
|  |  |  | M | 50 |  |  | 6.3 | 4.0 | 12.1 |  | 14.7 | 10.9 | 13.8 |  | 5.0 | 3.4 | 29.9 |
|  |  |  | M | 60 |  |  | 3.2 | 2.5 | 11.6 |  | 10.6 | 9.5 | 20.9 |  | 3.3 | 2.3 | 36.1 |
|  |  |  | F | 40 |  |  | 1.9 | 2.1 | 19.7 |  | 5.0 | 3.7 | 23.0 |  | 1.6 | 1.1 | 42.1 |
|  |  |  | F | 50 |  |  | 0.8 | 0.5 | 24.1 |  | 2.2 | 0.9 | 28.7 |  | 0.2 | 0.1 | 42.5 |
|  |  |  | F | 60 |  |  | 0.1 | 0.1 | 18.0 |  | 0.7 | 0.3 | 26.8 |  | 0.0 | 0.0 | 54.0 |
|  |  |  |  |  |  |  |  |  |  |  |  |  |  |  |  |  |  |
|  |  |  |  |  |  |  |  |  |  |  |  |  |  |  |  |  |  |
|  |  |  |  |  |  |  |  |  |  |  |  |  |  |  |  |  |  |
| VIP (15) *continued* | 2002-2007 |  | M | 40 |  |  | 5.6 | 2.4 | 4.7 |  | 12.3 | 4.3 | 4.0 |  | 15.8 | 6.6 | 44.2 |
|  |  |  | M | 50 |  |  | 5.9 | 3.2 | 8.1 |  | 15.3 | 9.8 | 9.4 |  | 8.5 | 5.7 | 34.0 |
|  |  |  | M | 60 |  |  | 3.5 | 2.6 | 10.5 |  | 13.9 | 12.2 | 19.3 |  | 3.6 | 2.9 | 31.6 |
|  |  |  | F | 40 |  |  | 2.1 | 2.0 | 10.2 |  | 7.9 | 3.9 | 15.0 |  | 3.9 | 2.3 | 52.8 |
|  |  |  | F | 50 |  |  | 2.0 | 1.1 | 18.3 |  | 5.6 | 2.8 | 27.7 |  | 0.6 | 0.5 | 41.3 |
|  |  |  | F | 60 |  |  | 0.6 | 0.3 | 17.9 |  | 2.4 | 0.8 | 32.5 |  | 0.1 | 0.1 | 45.3 |
|  |  |  |  |  |  |  |  |  |  |  |  |  |  |  |  |  |  |
| Gastric cancer CC (17) | 1989-95 | [44] | M | 40-79 | /W | A/R | 5.4 | ← | 17.3 |  | 14.6 | ← | 30.2 |  | 4.6 | ← | 27.9 |
|  |  |  |  |  |  |  |  |  |  |  |  |  |  |  |  |  |  |
| NTS (22) | 1985 | [50] | M | 18-34 | /R | A/R | 4.1 | 21.6 | ← |  | 15.3 | 11.5 | ← |  | 11.3 | 36.2 | ← |
|  |  |  | M | 35-70 |  |  | 1.9 | 30.5 | ← |  | 6.2 | 24.4 | ← |  | 4.4 | 32.7 | ← |
|  |  |  | M | 18-70 |  |  | 2.7 | 27.0 | ← |  | 9.8 | 19.3 | ← |  | 7.1 | 34.1 | ← |
|  |  |  |  |  |  |  |  |  |  |  |  |  |  |  |  |  |  |
|  | 1986 | [51] | M | 18-34 | /R | A/R | 1.9 | 23.6 | ← |  | 7.9 | 9.8 | ← |  | 19.2 | 37.7 | ← |
|  |  |  | M | 35-70 |  |  | 1.4 | 25.7 | ← |  | 11.2 | 27.8 | ← |  | 1.7 | 32.3 | ← |
|  |  |  | M | 18-70 |  |  | 1.6 | 24.9 | ← |  | 10.0 | 21.3 | ← |  | 8.0 | 34.2 |  |
|  |  |  |  |  |  |  |  |  |  |  |  |  |  |  |  |  |  |
|  | 1987 | [52] | M | 18-34 | /R | A/R | 5.5 | 5.7 | 11.7 |  | 11.0 | 3.2 | 8.7 |  | 16.0 | 2.1 | 36.2 |
|  |  |  | M | 35-70 |  |  | 3.0 | 4.0 | 23.3 |  | 3.8 | 5.0 | 28.2 |  | 0.9 | 0.3 | 31.6 |
|  |  |  | M | 18-70 |  |  | 3.9 | 4.6 | 18.8 |  | 6.6 | 4.3 | 20.8 |  | 6.7 | 1.0 | 33.4 |
|  |  |  |  |  |  |  |  |  |  |  |  |  |  |  |  |  |  |
| Your country and your life (23) | 2001-02 | [53] | M | 16-79 | /R | A/R | 1.5 | 0.6 | 12.5 |  | 8.4 | 3.5 | 15.9 |  | 9.9 | 2.9 | 44.7 |
|  |  |  |  |  |  |  |  |  |  |  |  |  |  |  |  |  |  |
| Stockholm Fire Brigade (25) | 1993 | [55] | M | 35-60 | ^f^ | U/R | 3.3 | 1.3 | 17.2 |  | 13.9 | 17.2 | ← |  | 19.2 | ←* | 27.8 |
|  |  |  |  |  |  |  |  |  |  |  |  |  |  |  |  |  |  |
| Stockholm Public Health follow-up (26) | 2007 | [60] | M | 23-89 | /R | U/R | 26.8^g^ | ← | 7.9 |  | ←* | ←* | 18.3 |  | 5.0 | 3.1 | 38.9 |
|  |  |  |  |  |  |  |  |  |  |  |  |  |  |  |  |  |  |
|  |  |  |  |  |  |  |  |  |  |  |  |  |  |  |  |  |  |
|  |  |  |  |  |  |  |  |  |  |  |  |  |  |  |  |  |  |
| Skåne Public Health (28) | 2004 | [62] | M | 18-80 | /R | U/R | 3.8 | 15.3 | ← |  | 7.8 | 21.0 | ← |  | 7.9 | 44.2 | ← |
|  |  |  | F | 18-80 |  |  | 0.5 | 21.8 | ← |  | 1.2 | 20.5 | ← |  | 0.6 | 55.4 | ← |
|  |  |  |  |  |  |  |  |  |  |  |  |  |  |  |  |  |  |
| Stockholm family diabetes (29) | 1992-94 | [63] | M | 35-56 | /R | C/R | 24.8 | ← | 18.6 |  | ←* | ←* | 18.1 |  | 4.3 | 2.0 | 32.2 |
|  |  |  |  |  |  |  |  |  |  |  |  |  |  |  |  |  |  |
| Vara municipality (32) ^i^ | 2001-03 | [66] | M | 30-75 | /W | A/R |  |  |  |  |  |  |  |  |  |  |  |
|  |  |  |  |  |  |  |  |  |  |  |  |  |  |  |  |  |  |
| AIR (33) | unknown | [67] | M | 58 | /R | C/R | 3.6 | 3.1 | 17.9 |  | 8.2 | 4.9 | 25.8 |  | 0.5 | 0.5 | 35.5 |
|  |  |  |  |  |  |  |  |  |  |  |  |  |  |  |  |  |  |
| Stockholm county 60 year olds (34) | 1997-99 | [68] |  | 60 | /W | U/R | 1.5 | 0.0^h^ | 19.4 |  | 6.1 | 0.0^h^ | 39.6 |  | 0.9 | 0.6 | 32.0 |
| ← Category combined with category to the left.  ←* Category combined with dual users category further to the left  ^a^ For case-control (CC) studies data shown are for controls. Number in brackets refers to text section numbers within §3.2  ^b^ Frequency of snus use : /R regular or daily, /W weekly, /A any (regular or occasional, /U unspecified  ^c^ First code show product smoked : C cigarettes, A any (cigarettes, pipe or cigar), U unspecified. Second code shows frequency of smoking, as in footnote b.  ^d^ No subjects reported – see §3.2.2  ^e^ Matched on age to cases, who were mean age 72 (males) and 69 (females)  ^f^ Dual user: daily or occasional, exclusive user: daily – see §3.2.25  ^g^ May be inflated by subjects with inconsistent replies – see §3.2.26  ^h^ Assumed to be zero – see §3.2.34  ^i^ Percentages cannot be calculated due to excluded category | | | | | | | | | | | | | | | | | |

## TABLE 5 Ratios of switchers to non-switchers

| Study^a^ | Year^b^ | Source | Sex | Age^c^ | Definitions | | Switchers |  | Non-switchers | |  | Ratio | | | |
| --- | --- | --- | --- | --- | --- | --- | --- | --- | --- | --- | --- | --- | --- | --- | --- |
|  |  |  |  |  | Snus^d^ | Smok^e^ | (S1)^f^ |  | (N1)^g^ | (N2)^h^ |  | S1:N1 | S1:N2 | Other^i^ |  |
|  |  |  |  |  |  |  |  |  |  |  |  |  |  |  |  |
| ULF (3) – cross-sectional | 1980-81 | [16] | M | 16-24 | /A | A/R | 72 |  |  | 215 |  |  | 0.33 |  |  |
|  |  |  | M | 25-44 |  |  | 203 |  |  | 847 |  |  | 0.24 |  |  |
|  |  |  | M | 45-64 |  |  | 80 |  |  | 574 |  |  | 0.14 |  |  |
|  |  |  | M | 65-84 |  |  | 131 |  |  | 451 |  |  | 0.29 |  |  |
|  |  |  | M | 16-84 |  |  | 487 |  |  | 2086 |  |  | 0.23 |  |  |
|  |  |  |  |  |  |  |  |  |  |  |  |  |  |  |  |
|  | 1988-89 |  | M | 16-24 | /A | A/R | 63 |  |  | 87 |  |  | 0.72 |  |  |
|  |  |  | M | 25-44 |  |  | 235 |  |  | 483 |  |  | 0.49 |  |  |
|  |  |  | M | 45-64 |  |  | 114 |  |  | 439 |  |  | 0.26 |  |  |
|  |  |  | M | 65-84 |  |  | 101 |  |  | 249 |  |  | 0.41 |  |  |
|  |  |  | M | 16-84 |  |  | 512 |  |  | 1257 |  |  | 0.41 |  |  |
|  |  |  | F | 16-84 |  |  | 26 |  |  | 1618 |  |  | 0.02 |  |  |
|  |  |  |  |  |  |  |  |  |  |  |  |  |  |  |  |
|  | 1996-97 |  | M | 16-24 | /A | A/R | 85 |  |  | 79 |  |  | 1.08 |  |  |
|  |  |  | M | 25-44 |  |  | 290 |  |  | 270 |  |  | 1.07 |  |  |
|  |  |  | M | 45-64 |  |  | 184 |  |  | 313 |  |  | 0.59 |  |  |
|  |  |  | M | 65-84 |  |  | 62 |  |  | 129 |  |  | 0.48 |  |  |
|  |  |  | M | 16-84 |  |  | 620 |  |  | 790 |  |  | 0.78 |  |  |
|  |  |  | F | 16-84 |  |  | 51 |  |  | 1308 |  |  | 0.04 |  |  |
|  |  |  |  |  |  |  |  |  |  |  |  |  |  |  |  |
|  | 2004 |  | M | 16-24 | /A | A/R | 46 |  |  | 19 |  |  | 2.42 |  |  |
|  |  |  | M | 25-44 |  |  | 141 |  |  | 66 |  |  | 2.14 |  |  |
|  |  |  | M | 45-64 |  |  | 140 |  |  | 167 |  |  | 0.84 |  |  |
|  |  |  | M | 65-84 |  |  | 32 |  |  | 38 |  |  | 0.84 |  |  |
|  |  |  | M | 16-84 |  |  | 359 |  |  | 291 |  |  | 1.23 |  |  |
|  |  |  | F | 16-84 |  |  | 69 |  |  | 450 |  |  | 0.15 |  |  |
|  |  |  |  |  |  |  |  |  |  |  |  |  |  |  |  |
| ULF (3) follow-up | 1980-81, 8y | [18] | M | 16-84 | /R | A/R | S4: 32 |  | N3:450 |  |  |  |  | S4:N3 0.07 |  |
|  |  |  |  |  |  |  | S5: 69 |  | N4: 480 | N5: 530 |  |  |  | S5:N4 0.14 | S5:N5 0.13 |
|  |  |  |  |  |  |  |  |  |  |  |  |  |  |  |  |
|  |  |  |  |  |  |  |  |  |  |  |  |  |  |  |  |
|  | 1988-89, 8 y | [13] | M | 16-44 | /R | U/R | S4: 25 |  | N3:110 |  |  |  |  | S4:N3 0.23 |  |
|  |  |  |  |  |  |  | S5: 43 |  | N4: 115 | N5: 137 |  |  |  | S5:N4 0.37 | S5:N5 0.31 |
|  |  |  |  |  |  |  |  |  |  |  |  |  |  |  |  |
|  |  |  |  |  |  |  |  |  |  |  |  |  |  |  |  |
|  |  |  |  |  |  |  |  |  |  |  |  |  |  |  |  |
|  |  |  |  |  |  |  |  |  |  |  |  |  |  |  |  |
|  |  |  |  |  |  |  |  |  |  |  |  |  |  |  |  |
|  |  |  |  |  |  |  |  |  |  |  |  |  |  |  |  |
| ULF (3) follow-up |  |  | M | 45-84 | /R | U/R | S4: 19 |  | N3:180 |  |  |  |  | S4:N3 0.11 |  |
| *continued* |  |  |  |  |  |  | S5: 31 |  | N4: 188 | N5: 207 |  |  |  | S5:N4 0.16 | S5:N5 0.15 |
|  |  |  |  |  |  |  |  |  |  |  |  |  |  |  |  |
|  |  |  |  |  |  |  |  |  |  |  |  |  |  |  |  |
|  |  |  |  | 16-84 | /R | U/R | S4: 44 |  | N3:290 |  |  |  |  | S4:N3 0.15 |  |
|  |  |  |  |  |  |  | S5: 74 |  | N4: 303 | N5: 344 |  |  |  | S5:N4 0.24 | S5:N5 0.22 |
|  |  |  |  |  |  |  |  |  |  |  |  |  |  |  |  |
|  |  |  |  |  |  |  |  |  |  |  |  |  |  |  |  |
| SALT (4) | 1998-2002 | [19] | M | 42-64 | /A | C/A | 1368 |  | 2480 | 2639 |  | 0.55 | 0.52 |  |  |
|  |  | [20] | M | 42-64 | /R | C/R | S3: 1701 |  | 2120 |  |  |  |  | S3:N1 0.80 |  |
|  |  |  |  |  | /A | C/R | S3: 1838 |  | 2120 |  |  |  |  | S3:N1 0.87 |  |
|  |  |  |  |  | /A | C/A | S3: 1928 |  | 2278 |  |  |  |  | S3:N1 0.85 |  |
|  |  | [21] | M | 42-64 | /A | C/A | 1470 |  | 2416 |  |  | 0.61 |  |  |  |
|  |  |  |  |  |  |  |  |  |  |  |  |  |  |  |  |
| 2 AMI CC (6) | 1992-94 | [23] | M | 45-70 | /U | C/U | 94 |  | 460 | 472 |  | 0.20 | 0.20 |  |  |
|  |  |  |  |  |  |  |  |  |  |  |  |  |  |  |  |
| MONICA cross-sectional (7) | 1986 | [29] | M | 25-64 | /R | C/R | 73 |  | 121 | 153 |  | 0.60 | 0.48 |  |  |
|  |  | [26] | M | 25-64 | /U | A/A | 51 |  | 30 | 110 |  | 1.70 | 0.46 |  |  |
|  |  |  |  |  |  |  |  |  |  |  |  |  |  |  |  |
|  | 1990 | [29] | M | 25-64 | /R | C/R | 61 |  | 115 | 138 |  | 0.53 | 0.44 |  |  |
|  |  |  | F | 25-64 | /R | C/R | 8 |  | 199 | 199 |  | 0.04 | 0.04 |  |  |
|  |  |  |  |  |  |  |  |  |  |  |  |  |  |  |  |
|  | 1994 | [29] | M | 25-74 | /R | C/R | 90 |  | 97 | 112 |  | 0.93 | 0.80 |  |  |
|  |  |  | F | 25-74 | /R | C/R | 8 |  | 204 | 204 |  | 0.04 | 0.04 |  |  |
|  |  |  |  |  |  |  |  |  |  |  |  |  |  |  |  |
|  | 1999 | [29] | M | 25-74 | /R | C/R | 95 |  | 47 | 74 |  | 2.02 | 1.28 |  |  |
|  |  |  | F | 25-74 | /R | C/R | 30 |  | 148 | 155 |  | 0.20 | 0.19 |  |  |
|  |  |  |  |  |  |  |  |  |  |  |  |  |  |  |  |
| MONICA follow-up (8) | 1986, 13 y | [32] | M | 25-64 | /A | C/R | S6: 7 |  | N6: 34 |  |  |  |  | S6:N6 0.21 |  |
|  |  |  | M |  |  |  | S4: 11 |  | N3: 42 |  |  |  |  | S4:N3 0.26 |  |
|  |  |  | M |  |  |  | S5: 25 |  | N4: 44 | N5: 55 |  |  |  | S5:N4 0.57 | S5:N5 0.45 |
|  |  |  |  |  |  |  |  |  |  |  |  |  |  |  |  |
|  | 1990, 9 y |  | M | 25-64 | /A | C/R | S6: 5 |  | N6: 37 |  |  |  |  | S6:N6 0.14 |  |
|  |  |  | M |  |  |  | S4: 13 |  | N3: 42 |  |  |  |  | S4:N3 0.31 |  |
|  |  |  | M |  |  |  | S5: 24 |  | N4: 43 | N5: 63 |  |  |  | S5:N4 0.56 | S5:N5 0.38 |
|  |  |  |  |  |  |  |  |  |  |  |  |  |  |  |  |
|  | 1994, 5 y |  | M | 25-64 | /A | C/R | S6: 3 |  | N6: 42 |  |  |  |  | S6:N6 0.07 |  |
|  |  |  | M |  |  |  | S4: 5 |  | N3: 46 |  |  |  |  | S4:N3 0.11 |  |
|  |  |  | M |  |  |  | S5: 9 |  | N4: 47 | N5: 59 |  |  |  | S5:N4 0.19 | S5:N5 0.15 |
|  |  |  |  |  |  |  |  |  |  |  |  |  |  |  |  |
|  |  |  |  |  |  |  |  |  |  |  |  |  |  |  |  |
| MONICA follow-up (8) *continued* | 1986, 13 y |  | F | 25-64 | /A | C/R | S5: 5 |  | N4: 90 | N5: 92 |  |  |  | S5:N4 0.06 | S5:N5 0.05 |
|  | 1990, 9 y |  | F | 25-64 | /A | C/R | S5: 3 |  | N4: 86 | N5: 87 |  |  |  | S5:N4 0.03 | S5:N5 0.03 |
|  | 1994, 5 y |  | F | 25-64 | /A | C/R | S5: 6 |  | N4: 113 | N5: 114 |  |  |  | S5:N4 0.05 | S5:N5 0.05 |
|  |  |  |  |  |  |  |  |  |  |  |  |  |  |  |  |
| Malmö diet (9) | 1991-96 | [33] | M | 45-73 | /U | U/A | 420 |  |  | 2776 |  |  | 0.15 |  |  |
|  |  |  | F | 45-73 | /U | U/A | 27 |  |  | 4676 |  |  | 0.01 |  |  |
|  |  |  |  |  |  |  |  |  |  |  |  |  |  |  |  |
| IBD CC Stockholm (11) | 1984-87 | [34] | M | 15-79 | /R | C/U | 5 |  | 46 |  |  | 0.11 |  |  |  |
|  |  |  |  |  |  |  |  |  |  |  |  |  |  |  |  |
| Oral cancer CC (14) | 1980-89 | [37] | M+F | ^j^ | /R | A/R | 15 |  | 71 | 72 |  | 0.21 | 0.21 |  |  |
|  |  |  |  |  |  |  |  |  |  |  |  |  |  |  |  |
| VIP cross-sectional(15) | 1990-95 | [40] | M | 40 | /U | U/A | 642 |  | 657 | 975 |  | 0.98 | 0.66 |  |  |
|  |  |  | M | 50 |  |  | 436 |  | 775 | 965 |  | 0.56 | 0.45 |  |  |
|  |  |  | M | 60 |  |  | 297 |  | 496 | 584 |  | 0.60 | 0.51 |  |  |
|  |  |  | F | 40 |  |  | 95 |  | 1669 | 1742 |  | 0.06 | 0.05 |  |  |
|  |  |  | F | 50 |  |  | 21 |  | 1437 | 1447 |  | 0.01 | 0.01 |  |  |
|  |  |  | F | 60 |  |  | 8 |  | 720 | 724 |  | 0.01 | 0.01 |  |  |
|  |  |  |  |  |  |  |  |  |  |  |  |  |  |  |  |
|  | 1996-2001 |  | M | 40 | /U | U/A | 697 |  | 426 | 639 |  | 1.64 | 1.09 |  |  |
|  |  |  | M | 50 |  |  | 912 |  | 751 | 999 |  | 1.21 | 0.91 |  |  |
|  |  |  | M | 60 |  |  | 479 |  | 524 | 637 |  | 0.91 | 0.75 |  |  |
|  |  |  | F | 40 |  |  | 289 |  | 1137 | 1258 |  | 0.25 | 0.23 |  |  |
|  |  |  | F | 50 |  |  | 142 |  | 1551 | 1583 |  | 0.09 | 0.09 |  |  |
|  |  |  | F | 60 |  |  | 35 |  | 895 | 900 |  | 0.04 | 0.04 |  |  |
|  |  |  |  |  |  |  |  |  |  |  |  |  |  |  |  |
|  | 2002-2007 |  | M | 40 | /U | U/A | 745 |  | 285 | 430 |  | 2.61 | 1.73 |  |  |
|  |  |  | M | 50 |  |  | 971 |  | 514 | 717 |  | 1.89 | 1.35 |  |  |
|  |  |  | M | 60 |  |  | 891 |  | 673 | 840 |  | 1.32 | 1.06 |  |  |
|  |  |  | F | 40 |  |  | 497 |  | 641 | 767 |  | 0.78 | 0.65 |  |  |
|  |  |  | F | 50 |  |  | 375 |  | 1226 | 1300 |  | 0.31 | 0.29 |  |  |
|  |  |  | F | 60 |  |  | 159 |  | 1183 | 1203 |  | 0.13 | 0.13 |  |  |
|  |  |  |  |  |  |  |  |  |  |  |  |  |  |  |  |
| VIP follow-up (15) | 1990-97, 10y | [40] | M | 30, 40, 50 | /U | U/A | S4: 229 |  | N3: 916 |  |  |  |  | S4:N3 0.25 |  |
|  |  |  |  |  |  |  | S5: 573 |  | N4: 972 | N5: 1368 |  |  |  | S5:N4 0.59 | S5:N5 0.42 |
|  |  |  |  |  |  |  |  |  |  |  |  |  |  |  |  |
|  |  |  | F | 30, 40, 50 | /U | U/A | S4: 255 |  | N3: 1886 |  |  |  |  | S4:N3 0.14 |  |
|  |  |  |  |  |  |  | S5: 311 |  | N4: 1893 | N5: 2003 |  |  |  | S5:N4 0.16 | S5:N5 0.16 |
|  |  |  |  |  |  |  |  |  |  |  |  |  |  |  |  |
| Gastric cancer CC (17) | 1989-95 | [44] | M | 40-79 | /W | A/R | 114 |  | 135 |  |  | 0.84 |  |  |  |
|  |  |  |  |  |  |  |  |  |  |  |  |  |  |  |  |
| NTS (22) | 1985 | [50] | M | 18-34 | /R | A/R | 64 |  |  | 90 |  |  | 0.71 |  |  |
|  |  |  | M | 35-70 |  |  | 40 |  |  | 196 |  |  | 0.20 |  |  |
|  |  |  | M | 18-70 |  |  | 104 |  |  | 286 |  |  | 0.36 |  |  |
|  |  |  |  |  |  |  |  |  |  |  |  |  |  |  |  |
|  | 1986 | [51] | M | 18-34 | /R | A/R | 29 |  |  | 87 |  |  | 0.33 |  |  |
|  |  |  | M | 35-70 |  |  | 73 |  |  | 168 |  |  | 0.43 |  |  |
|  |  |  | M | 18-70 |  |  | 102 |  |  | 255 |  |  | 0.40 |  |  |
|  |  |  |  |  |  |  |  |  |  |  |  |  |  |  |  |
|  | 1987^k^ | [52] | M | 18-34 | /R | A/R | 48 |  | 51 | 76 |  | 0.94 | 0.63 |  |  |
|  |  |  |  |  |  |  | S3: 51 |  | 52 |  |  |  |  | S3:N1 0.98 |  |
|  |  |  |  |  |  |  |  |  |  |  |  |  |  |  |  |
|  |  |  | M | 35-70 |  |  | 27 |  | 164 | 192 |  | 0.16 | 0.14 |  |  |
|  |  |  |  |  |  |  | S3: 54 |  | 171 |  |  |  |  | S3:N1 0.32 |  |
|  |  |  |  |  |  |  |  |  |  |  |  |  |  |  |  |
|  |  |  | M | 18-70 |  |  | 75 |  | 215 | 268 |  | 0.35 | 0.28 |  |  |
|  |  |  |  |  |  |  | S3: 105 |  | 223 |  |  |  |  | S3:N1 0.47 |  |
|  |  |  |  |  |  |  |  |  |  |  |  |  |  |  |  |
| Your country and your life (23) | 2001-02 | [53] | M | 16-79 | /R | A/R | 263 |  | 391 | 411 |  | 0.67 | 0.64 |  |  |
|  |  |  |  |  |  |  | S2: 207 |  |  |  |  |  |  | S2:N1 0.53 |  |
|  |  |  |  |  |  |  | S3: 338 |  |  |  |  |  |  | S3:N1 0.86 |  |
|  |  |  |  |  |  |  |  |  |  |  |  |  |  |  |  |
| Stockholm City Fire Brigade (25) | 1993 | [55] | M | 35-60 | /R | U/R | 21 |  | 26 | 28 |  | 0.81 | 0.75 |  |  |
|  |  |  |  |  |  |  |  |  |  |  |  |  |  |  |  |
| Skåne Public Health (28) | 2004 | [62] | M | 18-80 | /R | U/R | 925 |  |  | 1813 |  |  | 0.51 |  |  |
|  |  |  | F | 18-80 |  |  | 173 |  |  | 3061 |  |  | 0.06 |  |  |
|  |  |  |  |  |  |  |  |  |  |  |  |  |  |  |  |
| Vara municipality (32) | 2001-03 | [66] | M | 30-75 | /W | A/R | 116 |  |  | 109 |  |  | 1.06 |  |  |
|  |  |  |  |  |  |  |  |  |  |  |  |  |  |  |  |
| AIR (33) | unknown | [67] | M | 58 | /R | C/R | 32 |  | 70 | 82 |  | 0.46 | 0.39 |  |  |
|  |  |  |  |  |  |  |  |  |  |  |  |  |  |  |  |
| Stockholm county 60 year olds (34) | 1997-99 | [68] | M | 60 | /W | U/R | 113 |  | 360^l^ | 360^l^ |  | 0.31 | 0.31 |  |  |

footnotes on next page

^a^ For case-control (CC) studies data shown are for controls. Number in brackets refers to text section numbers within §3.2

^b^ Baseline year and length of follow-up for longitudinal studies

^c^ At baseline for longitudinal studies

^d^ Frequency of snus use : /R regular or daily, /W weekly, /A any (regular or occasional, /U unspecified

^e^ First code show product smoked : C cigarettes, A any (cigarettes, pipe or cigar), U unspecified. Second code shows frequency of smoking, as in footnote d.

^f^ Unless otherwise specified, the definition of a switcher is S1 (Ex smoking, current snus). Other definitions are

S2 : Ex smoking, current snus, started smoking first

S3 : Ex smoker, ever snus, started smoking first

S4 : Current exclusive smoker at baseline, current exclusive snus user at follow-up

S5 : Current smoker (exclusive or dual) at baseline, current exclusive snus user at follow-up

S6 : Current exclusive smoker and never snus at baseline, current exclusive snus user at follow-up.

^g^ Unless otherwise specified, the definition of a non-switcher shown in this column is N1 (Current smoker, never snus). Other definitions in this column are

N3 : Current exclusive smoker at both baseline and follow-up

N4 : Current smoker (exclusive or dual) at baseline, current exclusive smoker at follow-up

N6 : Current exclusive smoker and never snus at baseline, current exclusive smoker at follow-up

^h^ Unless otherwise specified, the definition of a non-switcher shown in this column is N2 (Current smoker, non snus). The other definition in this column is

N5 : Current smoker (exclusive or dual) at both baseline and follow-up

^i^ See footnotes f-h for definitions

^j^ Matched on age to cases, who were mean age 72 (males) and 69 (females)

^k^ See explanation in §3.2.22 for apparent discrepancy in estimates of N1

^l^ Assumed no current smoker/former snus user – see §3.2.34.

## TABLE 6 Detailed distribution of switching patterns – ULF study (section 3.2.3)

| Year | Age | Baseline | | N | Follow-up | | | | | | | |
| --- | --- | --- | --- | --- | --- | --- | --- | --- | --- | --- | --- | --- |
|  |  | Smoking | Snus |  | Neither |  | Snus only |  | Smoking only |  | Both |  |
|  |  |  |  |  |  |  |  |  |  |  |  |  |
| 1980-81 | 16-84 | Current | Current | 119 | 15 | (0.6%) | 37 | (1.6%) | 30 | (1.3%) | 37 | (1.6%) |
|  |  |  | Non | 643 | 148 | (6.2%) | 32 | (1.3%) | 450 | (18.9%) | 13 | (0.5%) |
|  |  | Non | Current | 286 | 74 | (3.1%) | 183 | (7.7%) | 14 | (0.6%) | 14 | (0.6%) |
|  |  |  | Non | 1334 | 1187 | (49.8%) | 67 | (2.8%) | 67 | (2.8%) | 13 | (0.5%) |
|  |  |  |  |  |  |  |  |  |  |  |  |  |
| 1988-89 | 16-44 | Current | Current | 38 | 3 | (0.3%) | 18 | (1.9%) | 5 | (0.5%) | 12 | (1.3%) |
|  |  |  | Non | 183 | 38 | (4.1%) | 25 | (2.7%) | 110 | (11.9%) | 10 | (1.1%) |
|  |  | Non | Current | 200 | 37 | (4.0%) | 152 | (16.5%) | 6 | (0.6%) | 5 | (0.5%) |
|  |  |  | Non | 503 | 432 | (46.8%) | 51 | (5.5%) | 18 | (1.9%) | 2 | (0.2%) |
|  |  |  |  |  |  |  |  |  |  |  |  |  |
|  | 45-84 | Current | Current | 37 | 4 | (0.3%) | 12 | (1.0%) | 8 | (0.6%) | 13 | (1.1%) |
|  |  |  | Non | 286 | 81 | (6.6%) | 19 | (1.5%) | 180 | (14.6%) | 6 | (0.5%) |
|  |  | Non | Current | 120 | 31 | (2.5%) | 83 | (6.7%) | 4 | (0.3%) | 2 | (0.2%) |
|  |  |  | Non | 789 | 765 | (62.1%) | 8 | (0.6%) | 14 | (1.1%) | 2 | (0.2%) |
|  |  |  |  |  |  |  |  |  |  |  |  |  |
|  | Total 16-84 | Current | Current | 75 | 7 | (0.3%) | 30 | (1.4%) | 13 | (0.6%) | 25 | (1.2%) |
|  |  |  | Non | 469 | 119 | (5.5%) | 44 | (2.0%) | 290 | (13.5%) | 16 | (0.7%) |
|  |  | Non | Current | 320 | 68 | (3.2%) | 235 | (10.9%) | 10 | (0.5%) | 7 | (0.3%) |
|  |  |  | Non | 1292 | 1197 | (55.5%) | 59 | (2.7%) | 32 | (1.5%) | 4 | (0.2%) |

## TABLE 7 Detailed distribution of switching patterns – SALT study (section 3.2.4)

| Smoking | | Regular snus | | | | Occasional snus | | | | Any snus | | | | Never snus |
| --- | --- | --- | --- | --- | --- | --- | --- | --- | --- | --- | --- | --- | --- | --- |
|  |  | snus first | cigs first | same time | snus only | snus first | cigs first | same time | snus only | snus first | cigs first | same time | snus only |  |
| Current | regular | 61 | 324 | 47 | - | 11 | 100 | 20 | - | 72 | 424 | 67 | - | 2120 |
|  |  | (0.4%) | (2.3%) | (0.3%) |  | (0.1%) | (0.7%) | (0.1%) |  | (0.5%) | (3.1%) | (0.5%) |  | (15.3%) |
|  | occasional | 16 | 57 | 13 | - | 2 | 13 | 3 | - | 18 | 70 | 16 | - | 158 |
|  |  | (0.1%) | (0.4%) | (0.1%) |  | (0.0%) | (0.1%) | (0.0%) |  | (0.1%) | (0.5%) | (0.1%) |  | (1.1%) |
|  | any | 77 | 381 | 60 | - | 13 | 113 | 23 | - | 90 | 494 | 83 | - | 2278 |
|  |  | (0.6%) | (2.8%) | (0.4%) |  | (0.1%) | (0.8%) | (0.2%) |  | (0.7%) | (3.6%) | (0.6%) |  | (16.5%) |
|  |  |  |  |  |  |  |  |  |  |  |  |  |  |  |
| Ex | regular | 147 | 1701 | 230 | - | 10 | 137 | 22 | - | 157 | 1838 | 252 | - | 2950 |
|  |  | (1.1%) | (12.3%) | (1.7%) |  | (0.1%) | (1.0%) | (0.2%) |  | (1.1%) | (13.3%) | (1.8%) |  | (21.3%) |
|  | occasional | 38 | 70 | 24 | - | 6 | 20 | 11 | - | 44 | 90 | 35 | - | 238 |
|  |  | (0.3%) | (0.5%) | (0.2%) |  | (0.0%) | (0.1%) | (0.1%) |  | (0.3%) | (0.7%) | (0.3%) |  | (1.7%) |
|  | any | 185 | 1771 | 254 | - | 16 | 157 | 33 | - | 201 | 1928 | 287 | - | 3188 |
|  |  | (1.3%) | (12.8%) | (1.8%) |  | (0.1%) | (1.1%) | (0.2%) |  | (1.5%) | (13.9%) | (2.1%) |  | (23.1%) |
|  |  |  |  |  |  |  |  |  |  |  |  |  |  |  |
| Ever | regular | 208 | 2025 | 277 |  | 21 | 237 | 42 |  | 229 | 2262 | 319 |  | 5070 |
|  |  | (1.5%) | (14.7%) | (2.0%) |  | (0.2%) | (1.7%) | (0.3%) |  | (1.7%) | (16.4%) | (2.3%) |  | (36.7%) |
|  | occasional | 54 | 127 | 37 | - | 8 | 33 | 14 | - | 62 | 160 | 51 | - | 396 |
|  |  | (0.4%) | (0.9%) | (0.3%) |  | (0.1%) | (0.2%) | (0.1%) |  | (0.4%) | (1.2%) | (0.4%) |  | (2.9%) |
|  | any | 262 | 2152 | 314 | - | 29 | 270 | 56 | - | 291 | 2422 | 370 | - | 5466 |
|  |  | (1.9%) | (15.6%) | (2.3%) |  | (0.2%) | (2.0%) | (0.4%) |  | (2.1%) | (17.5%) | (2.7%) |  | (39.5%) |
|  |  |  |  |  |  |  |  |  |  |  |  |  |  |  |
| Never |  | - | - | - | 976 | - | - | - | 60 | - | - | - | 1036 | - |
|  |  |  |  |  | (7.1%) |  |  |  | (0.4%) |  |  |  | (7.5%) | (0.0%) |

## TABLE 8 Detailed distribution of switching patterns – MONICA follow-up study (section 3.2.8)

| Sex | Baseline | | | N | Follow-up | | | | | | | |
| --- | --- | --- | --- | --- | --- | --- | --- | --- | --- | --- | --- | --- |
|  | Year | Smoking | Snus |  | Neither | | Snus only | | Smoking only | | Both | |
| **Men** |  |  |  |  |  |  |  |  |  |  |  |  |
|  | 1986 | current | current | 23 | 2 | (9.0%) | 14 | (60.0%) | 2 | (9.0%) | 5 | (22.0%) |
|  |  |  | ex | 20 | 5 | (25.0%) | 4 | (20.0%) | 8 | (40.0%) | 3 | (15.0%) |
|  |  |  | never | 72 | 28 | (39.0%) | 7 | (10.0%) | 34 | (47.0%) | 3 | (4.0%) |
|  |  | ex | current | 51 | 14 | (28.0%) | 33 | (64.0%) | 3 | (6.0%) | 1 | (2.0%) |
|  |  |  | ex | 47 | 32 | (68.0%) | 11 | (23.0%) | 4 | (9.0%) | 0 | (0.0%) |
|  |  |  | never | 90 | 88 | (98.0%) | 0 | (0.0%) | 2 | (2.0%) | 0 | (0.0%) |
|  |  | never | current | 51 | 16 | (31.0%) | 34 | (67.0%) | 0 | (0.0%) | 1 | (2.0%) |
|  |  |  | ex | 13 | 12 | (92.0%) | 0 | (0.0%) | 1 | (8.0%) | 0 | (0.0%) |
|  |  |  | never^a^ |  |  |  |  |  |  |  |  |  |
|  |  |  |  |  |  |  |  |  |  |  |  |  |
|  | 1990 | current | current | 27 | 3 | (11.0%) | 11 | (41.0%) | 1 | (4.0%) | 12 | (44.0%) |
|  |  |  | ex | 18 | 3 | (17.0%) | 8 | (44.0%) | 5 | (28.0%) | 2 | (11.0%) |
|  |  |  | never | 64 | 16 | (25.0%) | 5 | (8.0%) | 37 | (58.0%) | 6 | (9.0%) |
|  |  | ex | current | 44 | 14 | (32.0%) | 23 | (52.0%) | 2 | (5.0%) | 5 | (11.0%) |
|  |  |  | ex | 44 | 37 | (84.0%) | 6 | (14.0%) | 1 | (2.0%) | 0 | (0.0%) |
|  |  |  | never | 67 | 60 | (90.0%) | 4 | (6.0%) | 1 | (2.0%) | 1 | (2.0%) |
|  |  | never | current | 58 | 10 | (17.0%) | 47 | (81.0%) | 0 | (0.0%) | 1 | (2.0%) |
|  |  |  | ex | 34 | 30 | (88.0%) | 3 | (9.0%) | 1 | (3.0%) | 0 | (0.0%) |
|  |  |  | never^a^ |  |  |  |  |  |  |  |  |  |
|  |  |  |  |  |  |  |  |  |  |  |  |  |
|  | 1994 | current | current | 17 | 3 | (18.0%) | 4 | (24.0%) | 1 | (6.0%) | 9 | (52.0%) |
|  |  |  | ex | 8 | 1 | (13.0%) | 2 | (25.0%) | 4 | (49.0%) | 1 | (13.0%) |
|  |  |  | never | 59 | 12 | (20.0%) | 3 | (5.0%) | 42 | (72.0%) | 2 | (3.0%) |
|  |  | ex | current | 64 | 6 | (9.0%) | 55 | (86.0%) | 1 | (2.0%) | 2 | (3.0%) |
|  |  |  | ex | 32 | 28 | (88.0%) | 3 | (9.0%) | 1 | (3.0%) | 0 | (0.0%) |
|  |  |  | never | 75 | 67 | (89.0%) | 2 | (3.0%) | 6 | (8.0%) | 0 | (0.0%) |
|  |  | never | current | 44 | 3 | (7.0%) | 41 | (93.0%) | 0 | (0.0%) | 0 | (0.0%) |
|  |  |  | ex | 26 | 23 | (88.0%) | 2 | (8.0%) | 1 | (4.0%) | 0 | (0.0%) |
|  |  |  | never^a^ |  |  |  |  |  |  |  |  |  |
|  |  |  |  |  |  |  |  |  |  |  |  |  |
|  | All | current | current | 67 | 8 | (12.0%) | 29 | (43.0%) | 4 | (6.0%) | 26 | (39.0%) |
|  |  |  | ex | 46 | 9 | (20.0%) | 14 | (30.0%) | 17 | (37.0%) | 6 | (13.0%) |
|  |  |  | never | 195 | 57 | (29.0%) | 16 | (8.0%) | 111 | (57.0%) | 12 | (6.0%) |
|  |  | ex | current | 159 | 33 | (21.0%) | 111 | (70.0%) | 6 | (4.0%) | 8 | (5.0%) |
|  |  |  | ex | 123 | 97 | (79.0%) | 20 | (16.0%) | 6 | (5.0%) | 0 | (0.0%) |
|  |  |  | never | 232 | 216 | (93.0%) | 7 | (3.0%) | 9 | (4.0%) | 0 | (0.0%) |
|  |  | never | current | 153 | 29 | (19.0%) | 122 | (80.0%) | 0 | (0.0%) | 2 | (1.0%) |
|  |  |  | ex | 73 | 65 | (89.0%) | 5 | (7.0%) | 3 | (4.0%) | 0 | (0.0%) |
|  |  |  | never | 603 | 591 | (98.0%) | 12 | (2%) | ← |  | ← |  |
|  |  |  |  |  |  |  |  |  |  |  |  |  |
|  |  |  |  |  |  |  |  |  |  |  |  |  |
| Sex | Baseline |  |  | N | Follow-up | | | | | | | |
|  | Year | Smoking |  |  | Neither | | Snus only | | Smoking only | | Both | |
| **Women^b^** |  |  |  |  |  |  |  |  |  |  |  |  |
|  | 1986 | cur |  | 153 | 57 | (37.0%) | 5 | (3.0%) | 90 | (59.0%) | 2 | (1.0%) |
|  |  | ex |  | 95 | 89 | (94.0%) | 0 | (0.0%) | 6 | (6.0%) | 0 | (0.0%) |
|  |  |  |  |  |  |  |  |  |  |  |  |  |
|  | 1990 | cur |  | 125 | 35 | (28.0%) | 3 | (2.0%) | 86 | (69.0%) | 1 | (1.0%) |
|  |  | ex |  | 96 | 87 | (91.0%) | 2 | (2.0%) | 7 | (7.0%) | 0 | (0.0%) |
|  |  |  |  |  |  |  |  |  |  |  |  |  |
|  | 1994 | cur |  | 145 | 25 | (17.0%) | 6 | (4.0%) | 113 | (78.0%) | 1 | (1.0%) |
|  |  | ex |  | 140 | 126 | (90.0%) | 1 | (1.0%) | 13 | (9.0%) | 0 | (0.0%) |
|  |  |  |  |  |  |  |  |  |  |  |  |  |
|  | All | cur |  | 423 | 114 | (27.0%) | 13 | (3.0%) | 292 | (69.0%) | 4 | (1.0%) |
|  |  | ex |  | 331 | 301 | (91.0%) | 3 | (1.0%) | 26 | (8.0%) | 0 | (0.0%) |

^a^ Information available only for all years combined

^b^ No information at baseline for women never smokers, or by snus use

## TABLE 9 Detailed distribution of switching patterns – VIP follow-up study (section 3.2.15)

| Sex | Baseline | | N | Followup | | | | | | | |
| --- | --- | --- | --- | --- | --- | --- | --- | --- | --- | --- | --- |
|  | Smoking | Snus |  | Neither | | Snus only | | Smoking only | | Both | |
|  |  |  |  |  |  |  |  |  |  |  |  |
| **Men** | Current | current | 765 | 94 | (0.8%) | 344 | (3.0%) | 56 | (0.5%) | 263 | (2.3%) |
|  |  | non | 1792 | 514 | (4.4%) | 229 | (2.0%) | 916 | (7.9%) | 133 | (1.1%) |
|  | non | current | 2218 | 452 | (3.9%) | 1630 | (14.0%) | 24 | (0.2%) | 111 | (1.0%) |
|  |  | non | 6846 | 6380 | (54.9%) | 288 | (2.5%) | 151 | (1.3%) | 27 | (0.2%) |
|  |  |  |  |  |  |  |  |  |  |  |  |
| **Women** | current | current | 113 | 21 | (0.2%) | 56 | (0.4%) | 7 | (0.1%) | 29 | (0.2%) |
|  |  | non | 3361 | 1139 | (8.5%) | 255 | (1.9%) | 1886 | (14.1%) | 81 | (0.6%) |
|  | non | current | 369 | 79 | (0.6%) | 260 | (1.9%) | 7 | (0.1%) | 23 | (0.2%) |
|  |  | non | 9520 | 9082 | (68.0%) | 133 | (1.0%) | 286 | (2.1%) | 19 | (0.1%) |

## TABLE 10 Meta-analysis of ORs for ever snus use and ever smoking

| Sex | Factor | Level | N^a^ | Fixed-effect estimate OR (95% CI) | Random-effects estimate OR (95% CI) | | Heterogeneity within level per d.f.(p)^b^ | Heterogeneity  between levels^b^ |
| --- | --- | --- | --- | --- | --- | --- | --- | --- |
|  |  |  |  |  |  | |  |  |
| Any | ^–^ |  | 36 | 1.29 (1.27-1.30) | 3.13 (2.28-4.30) | | 229.9 *** |  |
|  |  |  |  |  |  | |  |  |
|  | Sex | Male | 25 | 1.22 (1.21-1.24) | 2.80 (1.94-4.06) | | 269.4 *** |  |
|  |  | Female | 9 | 5.03 (4.69-5.40) | 5.49 (4.14-7.30) | | 11.2 *** |  |
|  |  | Combined | 2 | 0.93 (0.70-1.22) | 1.10 (0.47-2.57) | | 7.4 ** | 3.7 * ^c^ |
|  |  |  |  |  |  | |  |  |
|  |  |  |  |  |  | |  |  |
| Males | Period | Before 1990 | 6 | 0.95 (0.94-0.97) | 1.13 (0.73-1.75) | | 47.2 *** |  |
|  |  | 1990-1999 | 12 | 4.18 (3.95-4.43) | 3.65 (3.01-4.42) | | 8.8 *** |  |
|  |  | 2000+ | 7 | 3.41 (3.29-3.55) | 3.61 (2.45-5.32) | | 99.6 *** | 65.4 *** |
|  |  |  |  |  |  | |  |  |
|  | Age | ≤45 | 7 | 1.15 (1.13-1.17) | 1.86 (1.29-2.68) | | 269.8 *** |  |
|  |  | 35-64 | 6 | 1.94 (1.88-2.00) | 2.63 (1.79-3.89) | | 143.2 *** |  |
|  |  | ≥55 | 6 | 2.12 (2.00-2.25) | 4.09 (1.69-9.91) | | 170.8 *** | 2.6 NS |
|  |  |  |  |  |  | |  |  |
|  | Region | Nationwide^d^ | 8 | 1.03 (1.02-1.05) | 2.55 (1.56-4.17) | | 181.1 *** |  |
|  |  | North | 11 | 4.64 (4.43-4.85) | 3.99 (3.40-4.68) | | 11.1 *** |  |
|  |  | South/Central | 6 | 1.90 (1.78-2.02) | 1.66 (0.55-4.99) | | 221.8 *** | 17.6 *** |
|  |  |  |  |  |  | |  |  |
|  | Regularity of snus use | Regular | 12 | 1.01 (1.00-1.03) | 2.12 (1.30-3.47) | | 156.5 *** |  |
|  |  | Any/unspecified | 13 | 3.63 (3.50-3.77) | 3.63 (2.78-4.75) | | 50.2 *** | 41.0 *** |
|  |  |  |  |  |  | |  |  |
|  |  |  |  |  |  | |  |  |
|  | Smoking product | Cigarettes | 9 | 2.47 (2.35-2.60) | 2.66 (1.90-3.72) | | 27.1 *** |  |
|  |  | Any/unspecified | 17 | 1.16 (1.14-1.18) | 2.94 (1.79-4.83) | | 345.1 *** | 3.2 (*) |
|  |  |  |  |  |  | |  |  |
| Data taken from Table 1. Method of selecting estimates and definitions of factors given in §2.8  ^a^ Number of estimates included  ^b^ p coded as *** p<0.001, p<0.01 **, p<0.05 *, 0.05 ≤ p < 0.1 (*), p≥0.1 NS  ^c^ Heterogeneity between males and females specifically is 7.2 (*)  ^d^ Including local studies in both regions | | | | | |  |  |  |

## TABLE 11 Percentages of dual users for ever snus use and ever smoking

| Sex | Factor | Level | N^a^ | Mean | Range | SE | |
| --- | --- | --- | --- | --- | --- | --- | --- |
|  |  |  |  |  |  |  | |
| Any | ^–^ |  | 36 | 18.2 | 2.9 - 44.0 | 1.7 | |
|  |  |  |  |  |  |  | |
|  | Sex | Male | 25 | 22.0 | 6.9 - 44.0 | 1.9 | |
|  |  | Female | 9 | 9.4 | 2.9 - 21.4 | 2.1 | |
|  |  | Combined | 2 | 10.0 | 7.2 - 12.7 | 2.8 | |
|  |  |  |  |  |  |  | |
|  |  |  |  |  |  |  | |
| Males | Period | Before 1990 | 6 | 14.0 | 6.9 - 23.7 | 2.8 | |
|  |  | 1990-1999 | 12 | 22.9 | 7.5 - 35.9 | 2.2 | |
|  |  | 2000+ | 7 | 27.4 | 14.0 - 44.0 | 4.0 | |
|  |  |  |  |  |  |  | |
|  | Age^d^ | ≤45 | 7 | 19.6 | 10.5 - 29.8 | 2.8 | |
|  |  | 35-64 | 6 | 23.5 | 14.9 - 35.9 | 4.0 | |
|  |  | ≥55 | 6 | 20.2 | 7.5 - 32.2 | 3.5 | |
|  |  |  |  |  |  |  | |
|  | Region | Nationwide^b^ | 8 | 20.2 | 12.8 - 44.0 | 3.2 | |
|  |  | North | 11 | 27.7 | 21.7 - 35.9 | 1.4 | |
|  |  | South/Central | 6 | 14.0 | 6.9 - 26.8 | 3.8 | |
|  |  |  |  |  |  |  | |
|  | Regularity of snus use | Regular | 12 | 19.6 | 6.9 - 28.7 | 2.1 | |
|  |  | Any/unspecified | 13 | 24.2 | 7.5 - 44.0 | 3.0 | |
|  |  |  |  |  |  |  | |
|  |  |  |  |  |  |  | |
|  | Smoking product | Cigarettes | 9 | 23.5 | 6.9 - 44.0 | 3.3 | |
|  |  | Any/unspecified | 17 | 21.8 | 6.9 - 35.9 | 2.3 | |
|  |  |  |  |  |  |  | |
| Data taken from Table 1. Method of selecting estimates and definitions of factors given in §2.8  ^a^ Number of estimates included  ^b^ Including local studies in both regions | | | | | | |  |

## TABLE 12 Meta-analysis of ORs for current snus use and current smoking

| Sex | Factor | Level | N^a^ | Fixed-effect estimate OR (95% CI) | Random-effects estimate OR (95% CI) | Heterogeneity within level per d.f.(p)^b^ | Heterogeneity  between levels^b^ |
| --- | --- | --- | --- | --- | --- | --- | --- |
|  |  |  |  |  |  |  |  |
| Any | ^–^ |  | 56 | 0.96 (0.93-0.99) | 1.05 (0.95-1.16) | 9.7 *** |  |
|  |  |  |  |  |  |  |  |
|  | Sex | Male | 36 | 1.01 (0.98-1.05) | 1.02 (0.91-1.14) | 9.0 *** |  |
|  |  | Female | 18 | 0.82 (0.77-0.87) | 1.12 (0.88-1.42) | 10.6 *** |  |
|  |  | Combined | 2 | 1.10 (0.74-1.62) | 1.10 (0.74-1.62) | 0.5 NS | 1.9 NS |
|  |  |  |  |  |  |  |  |
|  |  |  |  |  |  |  |  |
| Males | Period | Before 1990 | 10 | 1.06 (0.98-1.14) | 1.08 (0.86-1.36) | 7.0 *** |  |
|  |  | 1990-1999 | 13 | 1.11 (1.04-1.18) | 1.09 (0.97-1.21) | 2.4 ** |  |
|  |  | 2000+ | 13 | 0.94 (0.90-0.99) | 0.94 (0.75-1.17) | 17.0 *** | 1.0 NS |
|  |  |  |  |  |  |  |  |
|  | Age^d^ | ≤45 | 26 | 1.16 (1.09-1.24) | 1.19 (0.99-1.42) | 6.4 *** |  |
|  |  | 35-64 | 11 | 0.89 (0.83-0.95) | 0.87 (0.66-1.14) | 14.0 *** |  |
|  |  | ≥55 | 14 | 0.99 (0.91-1.09) | 0.98 (0.85-1.14) | 1.7 (*) | 2.5 (*) |
|  |  |  |  |  |  |  |  |
|  | Region | Nationwide^d^ | 14 | 0.90 (0.85-0.95) | 1.01 (0.82-1.25) | 13.2 *** |  |
|  |  | North | 14 | 1.15 (1.09-1.22) | 1.04 (0.90-1.21) | 5.2 *** |  |
|  |  | South/Central | 8 | 1.01 (0.94-1.08) | 1.03 (0.83-1.27) | 5.5 *** | 2.2 NS |
|  |  |  |  |  |  |  |  |
|  | Regularity of snus use | Regular | 12 | 0.90 (0.84-0.96) | 0.86 (0.73-1.00) | 4.0 *** |  |
|  |  | Any/unspecified | 24 | 1.06 (1.02-1.10) | 1.11 (0.96-1.28) | 11.1 *** | 1.9 NS |
|  |  |  |  |  |  |  |  |
|  |  |  |  |  |  |  |  |
|  | Smoking product | Cigarettes | 10 | 0.65 (0.59-0.72) | 0.85 (0.62-1.19) | 7.5 *** |  |
|  |  | Any/unspecified | 26 | 1.08 (1.04-1.12) | 1.08 (0.98-1.20) | 6.3 *** | 13.5 *** |
|  |  |  |  |  |  |  |  |
| Data taken from Table 2. Method of selecting estimates and definitions of factors given in §2.8  ^a^ Number of estimates included  ^b^ p coded as *** p<0.001, p<0.01 **, p<0.05 *, 0.05 ≤ p < 0.1 (*), p≥0.1 NS  ^c^ Heterogeneity between males and females specifically is 3.7 (*)  ^d^ Including local studies in both regions | | | | | | | |

## TABLE 13 Percentages of dual users for current snus use and current smoking

| Sex | Factor | Level | N^a^ | Mean | Range | SE | |
| --- | --- | --- | --- | --- | --- | --- | --- |
|  |  |  |  |  |  |  | |
| Any | ^–^ |  | 56 | 3.5 | 0.1 - 12.4 | 0.4 | |
|  |  |  |  |  |  |  | |
|  | Sex | Male | 36 | 4.8 | 1.5 - 12.4 | 0.5 | |
|  |  | Female | 18 | 0.9 | 0.1 - 2.1 | 0.2 | |
|  |  | Combined | 2 | 3.4 | 2.2 - 4.5 | 1.2 | |
|  |  |  |  |  |  |  | |
|  |  |  |  |  |  |  | |
| Males | Period | Before 1990 | 10 | 6.9 | 4.0 - 12.4 | 1.0 | |
|  |  | 1990-1999 | 13 | 4.2 | 1.5 - 8.3 | 0.5 | |
|  |  | 2000+ | 13 | 3.9 | 1.7 - 10.5 | 0.7 | |
|  |  |  |  |  |  |  | |
|  | Age^d^ | ≤45 | 26 | 6.6 | 1.0 - 19.7 | 1.0 | |
|  |  | 35-64 | 11 | 3.9 | 1.7 - 6.3 | 0.4 | |
|  |  | ≥55 | 14 | 2.1 | 0.2 - 3.7 | 0.3 | |
|  |  |  |  |  |  |  | |
|  | Region | Nationwide^b^ | 14 | 5.2 | 1.7 - 12.4 | 1.0 | |
|  |  | North | 14 | 4.7 | 2.0 - 8.3 | 0.5 | |
|  |  | South/Central | 8 | 4.4 | 1.5 - 10.5 | 1.0 | |
|  |  |  |  |  |  |  | |
|  | Regularity of snus use | Regular | 12 | 3.3 | 2.0 - 5.2 | 0.3 | |
|  |  | Any/unspecified | 24 | 5.6 | 1.5 - 12.4 | 0.6 | |
|  |  |  |  |  |  |  | |
|  |  |  |  |  |  |  | |
|  | Smoking product | Cigarettes | 10 | 4.1 | 1.7 - 8.3 | 0.6 | |
|  |  | Any/unspecified | 26 | 5.1 | 1.5 - 12.4 | 0.6 | |
|  |  |  |  |  |  |  | |
| Data taken from Table 2. Method of selecting estimates and definitions of factors given in §2.8  ^a^ Number of estimates included, taken from Table 2  ^b^ Including local studies in both regions | | | | | | |  |

References

Style BMJsqbrac

1. Lee PN. Summary of the epidemiological evidence relating snus to health. *Regul Toxicol Pharmacol* 2011;**59**:197-214.

2. Lee PN, Forey BA. *Gaining insight into the consequences of swithcing from cigarettes to snus. Report 1. Health risks of dual users or of switching to snus (including some data on joint use of cigarettes and snus).* Internal report. 2011.

3. Forey B, Hamling J, Hamling J, Lee P, editors. *International Smoking Statistics. A collection of historical data from 30 economically developed countries,* Web edition. Sutton, UK: P N Lee Statistics & Computing Ltd; 2006-2011. [www.pnlee.co.uk/iss.htm](http://www.pnlee.co.uk/iss.htm)

4. Berggren U, Eriksson M, Fahlke C, Blennow K, Balldin J. Different effects of smoking or use of smokeless tobacco on platelet MAO-B activity in type 1 alcohol-dependent subjects. *Alcohol Alcohol* 2007;**42**:267-71.

5. Ekberg G, Sjöfors G, Grefberg N, Larsson L-O, Vaara I. Protein intake and glomerular hyperfiltration in insulin-treated diabetics without manifest nephropathy. *Scand J Urol Nephrol* 1993;**27**:441-6.

6. Ekenvall L, Lindblad LE. Vibrationsutlösta Raynaudfenomen och nikotinkonsumtion - en preliminär rapport. (Vibration induced white fingers and nicotine - a preliminary report). *Opusc Med* 1985;**30**:28-31.

7. Fleiss JL, Gross AJ. Meta-analysis in epidemiology, with special reference to studies of the association between exposure to environmental tobacco smoke and lung cancer: a critique. *J Clin Epidemiol* 1991;**44**:127-39.

8. Unell L, Soderfeldt B, Halling A, Birkhed D. Attitudes to and experience of dental care among 50-year-olds in two Swedish counties. *Swed Dent J* 1999;**23**:87-96.

9. Nordenvall C, Nilsson PJ, Ye W, Nyrén O. Smoking, snus use and risk of right- and left-sided colon, rectal and anal cancer: a 37 year follow-up study. *Int J Cancer* 2011;**128**:157-65.

10. Zendehdel K, Nyrén O, Luo J, Dickman PW, Boffetta P, Englund A, *et al*. Risk of gastroesophageal cancer among smokers and users of Scandinavian moist snuff. *Int J Cancer* 2008;**122**:1095-9.

11. Carlens C, Hergens M-P, Grunewald J, Ekbom A, Eklund A, Höglund CO, *et al*. Smoking, use of moist snuff and risk of chronic inflammatory diseases. *Am J Respir Crit Care Med* 2010;**181**:1217-22.

12. Aro P, Ronkainen J, Storskrubb T, Vieth M, Engstrand L, Johansson S-E, *et al*. Use of tobacco products and gastrointestinal morbidity: an endoscopic population-based study (the Kalixanda study). *Eur J Epidemiol* 2010;**25**:741-50.

13. Stenbeck M, Hagquist C, Rosén M. The association of snus and smoking behaviour: a cohort analysis of Swedish males in the 1990s. *Addiction* 2009;**104**:1579-85.

14. Haglund B, Eliasson M, Stenbeck M, Rosén M. Is moist snuff use associated with excess risk of IHD or stroke? A longitudinal follow-up of snuff users in Sweden. *Scand J Public Health* 2007;**35**:618-22.

15. Johansson S-E, Sundquist K, Qvist J, Sundquist J. Smokeless tobacco and coronary heart disease a 12-year follow-up study. *Eur J Cardiovasc Prev Rehabil* 2005;**12**:387-92.

16. Högstorp E. *Tables on smoking and snus, based on Survey of Living Conditions in Sweden* [Personal communication to Lee PN]. 2005, 21 November.

17. Statistiska Centralbyrån (SCB Statistics Sweden). *Undersökningarna av levnadsförhållanden. Tobaksvanor efter indikator, ålder, kön (Surveys of living conditions. Tobacco Habits by indicator, age, gender) Tables downloaded from SCB Statistical database*. 2011, (accessed July 2011). <http://www.ssd.scb.se/databaser/makro/MainTable.asp?yp=tansss&xu=C9233001&omradekod=LE&omradetext=Living+conditions&lang=2&langdb=1>

18. Tillgren P, Haglund BJ, Lundberg M, Romelsjö A. The sociodemographic pattern of tobacco cessation in the 1980s: results from a panel study of living condition surveys in Sweden. *J Epidemiol Community Health* 1996;**50**:625-30.

19. Hansson J, Pedersen NL, Galanti MR, Andersson T, Ahlbom A, Hallqvist J, *et al*. Use of snus and risk for cardiovascular disease: results from the Swedish Twin Registry. *J Intern Med* 2009;**265**:717-24.

20. Furberg H, Bulik CM, Lerman C, Lichtenstein P, Pedersen NL, Sullivan PF. Is Swedish snus associated with smoking initiation or smoking cessation? *Tob Control* 2005;**14**:422-4.

21. Furberg H, Lichtenstein P, Pedersen NL, Bulik C, Sullivan PF. Cigarettes and oral snuff use in Sweden: Prevalence and transitions. *Addiction* 2006;**101**:1509-15.

22. Hedström AK, Bäärnhielm M, Olsson T, Alfredsson L. Tobacco smoking, but not Swedish snuff use, increases the risk of multiple sclerosis. *Neurology* 2009;**73**:696-701.

23. Hergens M-P, Ahlbom A, Andersson T, Pershagen G. Swedish moist snuff and myocardial infarction among men. *Epidemiology* 2005;**16**:12-6.

24. Huhtasaari F, Asplund K, Lundberg V, Stegmayr B, Wester PO. Tobacco and myocardial infarction: is snuff less dangerous than cigarettes? *BMJ* 1992;**305**:1252-6.

25. Huhtasaari F, Lundberg V, Eliasson M, Janlert U, Asplund K. Smokeless tobacco as a possible risk factor for myocardial infarction: a population-based study in middle-aged men. *J Am Coll Cardiol* 1999;**34**:1784-90.

26. Huhtasaari F, Asplund K, Wester PO. Cardiovascular risk factors in the Northern Sweden MONICA study. *Acta Med Scand* 1988;**224**:99-108.

27. Eliasson M, Asplund K, Nasic S, Rodu B. Influence of smoking and snus on the prevalence and incidence of type 2 diabetes amongst men: the northern Sweden MONICA study. *J Intern Med* 2004;**256**:101-10.

28. Ångman M, Eliasson M. Snus och blodtrych. Tvärsnittsstudie av viloblodtryck hos män i MONICA-studien i norra Sverige. (Snuff and blood pressure. Cross-sectional study of blood pressure in rest among men in the MONICA study in Northern Sweden). *Lakartidningen* 2008;**105**:3530-5. <http://www.lakartidningen.se/07engine.php?articleId=10812>;

29. Rodu B, Stegmayr B, Nasic S, Asplund K. Impact of smokeless tobacco use on smoking in northern Sweden. *J Intern Med* 2002;**252**:398-404.

30. Stegmayr B, Eliasson M, Rodu B. The decline of smoking in northern Sweden. *Scand J Public Health* 2005;**33**:321-4.

31. Eliasson M, Asplund K, Evrin P-E, Lundblad D. Relationship of cigarette smoking and snuff dipping to plasma fibrinogen, fibrinolytic variables and serum insulin. The Northern Sweden MONICA study. *Atherosclerosis* 1995;**113**:41-53.

32. Rodu B, Stegmayr B, Nasic S, Cole P, Asplund K. Evolving patterns of tobacco use in northern Sweden. *J Intern Med* 2003;**253**:660-5.

33. Janzon E, Hedblad B. Swedish snuff and incidence of cardiovascular disease. A population-based cohort study. *BMC Cardiovasc Disord* 2009;**9**:21.

34. Persson P-G, Hellers G, Ahlbom A. Use of oral moist snuff in inflammatory bowel disease. *Int J Epidemiol* 1993;**22**:1101-3.

35. Roosaar A, Johansson AL, Sandborgh-Englund G, Axéll T, Nyrén O. Cancer and mortality among users and nonusers of snus. *Int J Cancer* 2008;**123**:168-73.

36. Axéll T, Liedholm R. Occurrence of recurrent herpes labialis in an adult Swedish population. *Acta Odontol Scand* 1990;**48**:119-23.

37. Schildt E-B, Eriksson M, Hardell L, Magnuson A. Oral snuff, smoking habits and alcohol consumption in relation to oral cancer in a Swedish case-control study. *Int J Cancer* 1998;**77**:341-6.

38. Wennberg P, Eliasson M, Hallmans G, Johansson L, Boman K, Jansson J-H. The risk of myocardial infarction and sudden cardiac death amongst snuff users with or without a previous history of smoking. *J Intern Med* 2007;**262**:360-7.

39. Lundqvist G, Sandström H, Öhman A, Weinehall L. Patterns of tobacco use: a 10-year follow-up study of smoking and snus habits in a middle-aged Swedish population. *Scand J Public Health* 2009;**37**:161-7.

40. Norberg M, Lundqvist G, Nilsson M, Gilljam H, Weinehall L. Changing patterns of tobacco use in a middle-aged population - the role of snus, gender, age, and education. *Global Health Action* 2011;**4**:1-13.

41. Wikström AK, Stephansson O, Cnattingius S. Tobacco use during pregnancy and preeclampsia risk: effects of cigarette smoking and snuff. *Hypertension* 2010;**55**:1254-9.

42. Wikström AK, Cnattingius S, Stephansson O. Maternal Use of Swedish Snuff (Snus) and Risk of Stillbirth. *Epidemiology* 2010;**21**:772-8.

43. Wikström A-K, Cnattingius S, Galanti MR, Kieler H, Stephansson O. Effect of Swedish snuff (*snus*) on preterm birth. *BJOG* 2010;**117**:1005-10.

44. Ye W, Ekström AM, Hansson L-E, Bergström R, Nyrén O. Tobacco, alcohol and the risk of gastric cancer by sub-site and histologic type. *Int J Cancer* 1999;**83**:223-9.

45. Christensen T. *Euro-barometer 58.2: Health and developing countries, October-December 2002 [Computer file]*. Ann Arbor, Michigan: Inter-university Consortium for Political and Social Research; 2004, (accessed Oct 2010). <http://www.esds.ac.uk/findingData/snDescription.asp?sn=4810>

46. Bogdanovica I, Godfrey F, McNeill A, Britton J. Smoking prevalence in the European Union: a comparison of national and transnational prevalence survey methods and results. *Tob Control* 2011;**20**:e4.

47. Furberg H, Lichtenstein P, Pedersen NL, Thornton L, Bulik CM, Lerman C, *et al*. The STAGE cohort: a prospective study of tobacco use among Swedish twins. *Nicotine Tob Res* 2008;**10**:1727-35.

48. Gilljam H, Galanti MR. Role of *snus* (oral moist snuff) in smoking cessation and smoking reduction in Sweden. *Addiction* 2003;**98**:1183-9.

49. Novo M, Hammarström A, Janlert U. Smoking habits - a question of trend or unemployment? A comparison of young men and women between boom and recession. *Public Health* 2000;**114**:460-3.

50. Ramström LM. *Tobaksvanor i Sverige 1985. Resultat från NTS-Undersökningen 1985. (Tobacco habits in Sweden 1985. Results from an NTS study 1985)*. Stockholm: NTS; 1986. Tema Tobak Nr. 3.

51. Ramström LM, Tibblin H. *Tobaksvanor i Sverige 1986. Resultat från NTS-undersökningen 1986. (Smoking habits in Sweden 1986. Results from a National Council on Smoking and Health [NTS] study 1986)*. 1987. Tema Tobak No 5.

52. Ramström LM, Tibblin H. *Tobaksvanor i Sverige 1987. Resultat från NTS-undersökning 1987. (Tobacco habits in Sweden 1987. Results from study of National Association against Tobacco Damage 1987)*. Stockholm: NTS; 1988. Tema Tobak Nr. 7.

53. Ramström LM, Foulds J. Role of snus in initiation and cessation of tobacco smoking in Sweden. *Tob Control* 2006;**15**:210-4.

54. Wadman C. *Levnadsvanor - Tobaksvanor*. Statens Folkhälsoinstitut; 2009, (Accessed Oct 2010). <http://www.fhi.se/sv/Statistik-uppfoljning/Nationella-folkhalsoenkaten/Levnadsvanor/Tobaksvanor/>

55. Bolinder G, Norén A, Wahren J, de Faire U. Long-term use of smokeless tobacco and physical performance in middle-aged men. *Eur J Clin Invest* 1997;**27**:427-33.

56. Bolinder G, Norén A, de Faire U, Wahren J. Smokeless tobacco use and atherosclerosis: an ultrasonographic investigation of carotid intima media thickness in healthy middle-aged men. *Atherosclerosis* 1997;**132**:95-103.

57. Bolinder G. *Long-term use of smokeless tobacco: cardiovascular mortality and risk factors* [Thesis]. Stockholm: Karolinska Institute; 1997.

58. Bolinder G, de Faire U. Ambulatory 24-h blood pressure monitoring in healthy, middle-aged smokeless tobacco users, smokers, and nontobacco users. *Am J Hypertens* 1998;**11**:1153-63.

59. Engström K, Magnusson C, Galanti MR. Socio-demographic, lifestyle and health characteristics among snus users and dual tobacco users in Stockholm County, Sweden. *BMC Public Health* 2010;**10**:619-62.

60. Hansson J, Galanti MR, Magnusson C, Hergens M-P. Weight gain and incident obesity among male snus users. *BMC Public Health* 2011;**11**:371.

61. Hirsch JM, Livian G, Edward S, Noren JG. Tobacco habits among teenagers in the city of Göteborg, Sweden, and possible association with dental caries. *Swed Dent J* 1991;**15**:117-23.

62. Lindström M. Nicotine replacement therapy, professional therapy, snuff use and tobacco smoking: a study of smoking cessation strategies in southern Sweden. *Tob Control* 2007;**16**:410-6.

63. Persson PG, Carlsson S, Svanström L, Östenson CG, Efendic S, Grill V. Cigarette smoking, oral moist snuff use and glucose intolerance. *J Intern Med* 2000;**248**:103-10.

64. Rolandsson M, Hugoson A. Changes in tobacco habits. A prospective longitudinal study of tobacco habits among boys who play ice hockey. *Swed Dent J* 2003;**27**:175-84.

65. Salonen L, Axéll T, Helldén L. Occurrence of oral mucosal lesions, the influence of tobacco habits and an estimate of treatment time in an adult Swedish population. *J Oral Pathol Med* 1990;**19**:170-6.

66. Sundbeck M, Grahn M, Lönngren V, Månsson NO, Råstam L, Lindblad U. Snuff use associated with abdominal obesity in former smokers. *Scand J Public Health* 2009;**37**:487-93.

67. Wallenfeldt K, Hulthe J, Bokemark L, Wikstrand J, Fagerberg B. Carotid and femoral atherosclerosis, cardiovascular risk factors and C-reactive protein in relation to smokeless tobacco use or smoking in 58-year-old men. *J Intern Med* 2001;**250**:492-501.

68. Wändell PE, Bolinder G, de Faire U, Hellénius M-L. Association between metabolic effects and tobacco use in 60-year-old Swedish men. *Eur J Epidemiol* 2008;**23**:431-4.

69. Wennmalm A, Benthin G, Granström EF, Persson L, Petersson A-S, Winell S. Relation between tobacco use and urinary excretion of thromboxane A_2_ and prostacyclin metabolites in young men. *Circulation* 1991;**83**:1698-704.

70. Wickholm S, Söder P-Ö, Galanti MR, Söder B, Klinge B. Periodontal disease in a group of Swedish adult snuff and cigarette users. *Acta Odontol Scand* 2004;**62**:333-8.

71. Nordgren P, Ramström L. Moist snuff in Sweden - tradition and evolution. *Br J Addict* 1990;**85**:1107-12.

1. Now part of Västra Götaland county [↑](#footnote-ref-1)
