# Supplementary material for: Using data on snus use in Sweden to compare different modelling approaches to estimate the population health impact of introducing a smoke-free tobacco product
Source: BMC Public Health. 2019 Oct 29;19:1411. doi: 10.1186/s12889-019-7714-0 (PMC6819486; doi:10.1186/s12889-019-7714-0)
Supplement: Supplementary file 3 — Additional file 3: Additional details for Approach 3. [file 12889_2019_7714_MOESM3_ESM.docx]

Title : “Using data on snus use in Sweden to validate a published modelling approach for estimating the population health impact of introducing a smoke-free tobacco product”

Authors : Smilja Djurdjevic, Laszlo Pecze, Rolf Weitkunat, Frank Luedicke, John Fry and Peter Lee

**SUPPLEMENTARY FILE 3**

Additional details for Approach 3

Approach 3 compares projected tobacco distributions and mortality in two scenarios, the SNUS scenario, representing the historical situation, in which snus is available and individuals may currently or formerly have used snus and/or cigarettes, and the NO-SNUS scenario, representing the hypothetical situation, in which snus is not available. In 1980 data were available on the distribution of tobacco use in nine groups.

| Group | Smoking | Snus |
| --- | --- | --- |
| 1 | Never | Never |
| 2 | Never | Former |
| 3 | Never | Current |
| 4 | Former | Never |
| 5 | Former | Former |
| 6 | Former | Current |
| 7 | Current | Never |
| 8 | Current | Former |
| 9 | Current | Current |

In the historical (SNUS) scenario, five groups were considered based on the original nine groups.

| Group | Original groups combined |
| --- | --- |
| Never tobacco | 1 + 2 |
| Current cigarettes only | 7 + 8 |
| Current snus only | 3 |
| Current dual use | 9 |
| Former tobacco | 4 + 5 + 6 |

In the hypothetical (NO-SNUS) scenario, three groups were considered, again based on the original nine groups.

| Group | Original groups combined |
| --- | --- |
| Never cigarettes | 1 |
| Current cigarettes | 3 + 6 + 7 + 8 + 9 |
| Former cigarettes | 2 + 4 + 5 |

Thus, this scenario included as current smokers all those who were current users of either or both product and as former smokers all those who had used either or both products but did not currently use them. Never smokers included only those who had never used either product. Effectively it was assumed that cigarette smoking totally replaced snus use.

The historical scenario used five rather than nine groups based on two assumptions. One was that there was no increase in risk associated with former use of snus, consistent with epidemiological evidence that exclusive snus use is associated with little or no increase in the incidence of the smoking attributable diseases studied [1, 2]. This suggests that any risk of former snus use can be ignored, so indicating that groups 1 + 2, 4 + 5 and 7 + 8 could each be combined as having equivalent risk. Evidence that current snus users who formerly smoked (“switchers”) have risks very similar to those of never users who quit smoking (“quitters”) [3] also justifies the decision to count those originally in group 6 as having equivalent risk to the other former cigarette smoking groups 4 and 5.

PHIM requires estimates for each individual of the equivalent dose at the start of the simulations. For simplicity it assumes that smokers started smoking at age of 16 and the initial equivalent dose is calculated accordingly. For former smokers, it is also assumed that the given person started smoking at age of 16, but quit smoking according to the given quit time distribution. During the simulation the equivalent dose at the current time point is calculated from the previous value of the equivalent dose (ED) using also information on the disease- and age-specific half-life (H) and its current smoking status.

ED(a) = N * ED (a – 1) + (1 – N) * RE

N = exp (–ln (2) / H)

Where RE is the relative exposure, N is the negative exponential factor, and a is age.

The equivalent dose assumed in the NO-SNUS scenario is 1 in current cigarette smokers and 0 otherwise. In the SNUS scenario, the equivalent doses assumed are 1 for current cigarette smokers only, f for snus only and g for dual use, and 0 otherwise. In the main Approach 3 analysis, f is taken as 0, equivalent to snus on its own not increasing risk, and g is taken as 1, equivalent to dual users having the same risk as cigarette only smokers. This is consistent with epidemiological evidence indicating that relative risks for dual use are very similar to those for exclusive cigarette smoking [4]. However, sensitivity analyses were also conducted with f = 0.1 or 0.2 (and g fixed at 1) and with g = 0.9, 0.8 or 0.5 (and f fixed at 0).

In both scenarios, random numbers are used to divide the populations into distributions consistent with the Swedish national prevalence. For all ages combined, the 100,000 individuals in the main analysis started in the SNUS scenario with 31.02% never tobacco, 27.28% current cigarettes only, 5.27% current snus only, 4.44% current dual use, and 31.99% former tobacco, while the 100,000 individuals started in the NO-SNUS scenario with 28.59% never cigarettes, 42.92% current cigarettes and 28.49% former cigarettes.

Subsequently, individuals changed groups according to monthly tobacco transition probabilities (TTPs), with multiple transitions not being allowed in a single year in these analyses.

In the SNUS scenarios, there are 15 TTPs as transitions to never tobacco use are not possible. The table below shows the age-dependent values of these, labelled using the abbreviations N = never tobacco; C = current cigarettes only; S = current snus only; D = current dual use and F = former use, with for example PNC indicating the probability of the transition from N to C. The TTPs are classified as representing initiation, quitting, re-initiation or switching. The TTPs were derived so as to produce distribution of tobacco use similar to those seen in Sweden during follow-up.

|  | Initiation | | | Quitting | | | Reinitiation | | |
| --- | --- | --- | --- | --- | --- | --- | --- | --- | --- |
| Age | PNC | PNS | PND | PCF | PSF | PDF | PFC | PFS | PFD |
| 10-14 | 0.002 | 0.004 | 0.0005 | 0.002 | 0.002 | 0.002 | 0.0003 | 0.0002 | 0 |
| 15-19 | 0.001 | 0.003 | 0.0005 | 0.002 | 0.002 | 0.002 | 0.0006 | 0.0002 | 0 |
| 20-24 | 0.0005 | 0.0007 | 0.0003 | 0.001 | 0.001 | 0.001 | 0.0002 | 0.0002 | 0 |
| 25-29 | 0.0001 | 0 | 0 | 0.0005 | 0.0005 | 0.0005 | 0.0001 | 0.0001 | 0 |
| 30-34 | 0 | 0 | 0 | 0.0005 | 0.0005 | 0.0005 | 0.0002 | 0.0002 | 0 |
| 35-39 | 0 | 0 | 0 | 0.001 | 0.001 | 0.001 | 0.0002 | 0.0002 | 0 |
| 40-44 | 0 | 0 | 0 | 0.001 | 0.001 | 0.001 | 0.0002 | 0.0002 | 0 |
| 45-49 | 0 | 0 | 0 | 0.001 | 0.001 | 0.001 | 0.0001 | 0.0001 | 0 |
| 50-54 | 0 | 0 | 0 | 0.0015 | 0.0015 | 0.0015 | 0.0002 | 0.0001 | 0 |
| 55-59 | 0 | 0 | 0 | 0.001 | 0.001 | 0.001 | 0.0003 | 0.0002 | 0 |
| 60-64 | 0 | 0 | 0 | 0.002 | 0.002 | 0.002 | 0.0006 | 0.0002 | 0 |
| 65-69 | 0 | 0 | 0 | 0.003 | 0.003 | 0.003 | 0.0006 | 0.0002 | 0 |
| 70-74 | 0 | 0 | 0 | 0.004 | 0.004 | 0.004 | 0.0006 | 0.0002 | 0 |
| 75-79 | 0 | 0 | 0 | 0.004 | 0.004 | 0.004 | 0.0006 | 0.0002 | 0 |

|  | Switching between current use groups | | | | | |
| --- | --- | --- | --- | --- | --- | --- |
| Age | PCS | PCD | PSC | PSD | PDC | PDS |
| 10-14 | 0.001 | 0.0004 | 0.0005 | 0.0001 | 0.002 | 0.006 |
| 15-19 | 0.001 | 0.0004 | 0.0005 | 0.0001 | 0.002 | 0.006 |
| 20-24 | 0.001 | 0.0004 | 0.0005 | 0.0001 | 0.002 | 0.006 |
| 25-29 | 0.001 | 0.0004 | 0.0005 | 0.0001 | 0.002 | 0.01 |
| 30-34 | 0.001 | 0.0004 | 0.0005 | 0.0001 | 0.002 | 0.01 |
| 35-39 | 0.001 | 0.0004 | 0.0005 | 0.0001 | 0.002 | 0.02 |
| 40-44 | 0.001 | 0.0004 | 0.0005 | 0.0001 | 0.002 | 0.02 |
| 45-49 | 0.001 | 0.0004 | 0.0005 | 0.0001 | 0.002 | 0.02 |
| 50-54 | 0.001 | 0.0004 | 0.0005 | 0.0001 | 0.002 | 0.005 |
| 55-59 | 0.001 | 0.0004 | 0.0005 | 0.0001 | 0.002 | 0.005 |
| 60-64 | 0.001 | 0.0004 | 0.0005 | 0.0001 | 0.002 | 0.005 |
| 65-69 | 0.001 | 0.0004 | 0.0005 | 0.0001 | 0.002 | 0.008 |
| 70-74 | 0.001 | 0 | 0.0005 | 0.0001 | 0.002 | 0.008 |
| 75-79 | 0.001 | 0 | 0.0005 | 0.0001 | 0.002 | 0.008 |

In the NO-SNUS scenario, there are only three TTPs as indicated in the table below.

| Age | Initialization | Quitting | Reinitialization |
| --- | --- | --- | --- |
| 10-14 | 0.0065 | 0.002 | 0.0005 |
| 15-19 | 0.0045 | 0.002 | 0.0008 |
| 20-24 | 0.0015 | 0.001 | 0.0004 |
| 25-29 | 0.0001 | 0.0005 | 0.0002 |
| 30-34 | 0 | 0.0005 | 0.0004 |
| 35-39 | 0 | 0.001 | 0.0004 |
| 40-44 | 0 | 0.001 | 0.0004 |
| 45-49 | 0 | 0.001 | 0.0002 |
| 50-54 | 0 | 0.0015 | 0.0003 |
| 55-59 | 0 | 0.001 | 0.0005 |
| 60-64 | 0 | 0.002 | 0.0008 |
| 65-69 | 0 | 0.003 | 0.0008 |
| 70-74 | 0 | 0.004 | 0.0008 |
| 75-79 | 0 | 0.004 | 0.0008 |

Note that the TTPs for initialization and for reinitiation in the NO-SNUS scenario are the sum of the three TTPs in the SNUS scenario, while the TTPs for quitting are the same. This ensures comparability of the two scenarios.

References

1. Lee PN. Summary of the epidemiological evidence relating snus to health. Regul Toxicol Pharmacol 2011;59(2):197-214. doi:10.1016/j.yrtph.2010.12.002.

2. Lee PN. Epidemiological evidence relating snus to health - an updated review based on recent publications. Harm Reduct J 2013;10(1):36. doi:10.1186/1477-7517-10-36.

3. Lee PN. The effect on health of switching from cigarettes to snus - a review. Regul Toxicol Pharmacol 2013;66(1):1-5.

4. Lee PN. Health risks related to dual use of cigarettes and snus - A systematic review. Regul Toxicol Pharmacol 2014;69:125-34. doi:10.1016/j.yrtph.2013.10.007.
