# Supplementary material for: Using data on snus use in Sweden to compare different modelling approaches to estimate the population health impact of introducing a smoke-free tobacco product
Source: BMC Public Health. 2019 Oct 29;19:1411. doi: 10.1186/s12889-019-7714-0 (PMC6819486; doi:10.1186/s12889-019-7714-0)
Supplement: Supplementary file 4 — Additional file 4: Fuller details of results from the three approaches. [file 12889_2019_7714_MOESM4_ESM.docx]

Title : “Using data on snus use in Sweden to validate a published modelling approach for estimating the population health impact of introducing a smoke-free tobacco product”

Authors : Smilja Djurdjevic, Laszlo Pecze, Rolf Weitkunat, Frank Luedicke, John Fry and Peter Lee

**SUPPLEMENTARY FILE 4**

Fuller details of results from the three approaches

**Table 1. Increases in deaths in Sweden if snus had not been introduced – Approach 1**

|  | **Increase in Deaths** | | | | | | **Increase in Deaths (Adjusted)** | | | |
| --- | --- | --- | --- | --- | --- | --- | --- | --- | --- | --- |
| **Year** | **LC** | **COPD** | **IHD** | **STR** | **SRD** | **NSRD** | **LC** | **COPD** | **IHD** | **Stroke** |
| 1980 | 2092 | 1314 | -1837 | 2923 | 7781 | 7201 | 914 | 594 | -5543 | 1167 |
| 1981 | 2225 | 1269 | -1881 | 2680 | 7254 | 7536 | 971 | 548 | -5731 | 907 |
| 1982 | 2252 | 1252 | -1323 | 2863 | 7907 | 6933 | 1067 | 588 | -4924 | 1194 |
| 1983 | 2362 | 1420 | -480 | 3074 | 9343 | 7699 | 1055 | 656 | -4455 | 1239 |
| 1984 | 2384 | 1570 | -28 | 3199 | 9869 | 7825 | 1021 | 764 | -4172 | 1324 |
| 1985 | 2420 | 1608 | 169 | 3204 | 9851 | 8303 | 1007 | 739 | -4166 | 1255 |
| 1986 | 2756 | 1499 | 560 | 3175 | 10371 | 7146 | 1393 | 765 | -3369 | 1411 |
| 1987 | 2656 | 1356 | 1105 | 3132 | 9339 | 8143 | 1207 | 650 | -3098 | 1294 |
| 1988 | 2704 | 1338 | 1319 | 2973 | 9542 | 7363 | 1357 | 683 | -2469 | 1331 |
| 1989 | 2804 | 1453 | 2561 | 3151 | 10825 | 8667 | 1281 | 687 | -1778 | 1261 |
| 1990 | 2844 | 1347 | 3206 | 3137 | 11637 | 9395 | 1196 | 556 | -1626 | 1097 |
| 1991 | 2776 | 1205 | 3450 | 2814 | 11387 | 9584 | 1106 | 468 | -1365 | 828 |
| 1992 | 2773 | 1111 | 3829 | 2607 | 11845 | 10636 | 1011 | 371 | -1309 | 630 |
| 1993 | 2907 | 1176 | 3947 | 2666 | 11902 | 11680 | 1013 | 339 | -1416 | 579 |
| 1994 | 2775 | 1003 | 3968 | 2583 | 12035 | 12017 | 840 | 232 | -1398 | 459 |
| 1995 | 2901 | 1027 | 3759 | 2522 | 11914 | 11818 | 948 | 254 | -1406 | 509 |
| 1996 | 2791 | 855 | 3837 | 2357 | 10577 | 12231 | 854 | 141 | -1154 | 389 |
| 1997 | 2696 | 814 | 3529 | 2214 | 10367 | 11154 | 874 | 146 | -1020 | 377 |
| 1998 | 2774 | 936 | 3513 | 2203 | 10138 | 11457 | 925 | 218 | -883 | 354 |
| 1999 | 2687 | 970 | 3677 | 2284 | 10135 | 11648 | 840 | 226 | -767 | 420 |
| 2000 | 2667 | 859 | 3630 | 2205 | 9611 | 11369 | 846 | 168 | -558 | 390 |
| 2001 | 2635 | 774 | 3746 | 2283 | 9536 | 10884 | 876 | 154 | -271 | 561 |
| 2002 | 2541 | 756 | 3586 | 2192 | 9296 | 11069 | 799 | 121 | -339 | 477 |
| 2003 | 2488 | 827 | 3805 | 2242 | 9544 | 10929 | 767 | 178 | -95 | 554 |
| 2004 | 2540 | 765 | 3915 | 2095 | 9634 | 10469 | 824 | 169 | 190 | 529 |
| 2005 | 2373 | 839 | 4306 | 2014 | 9877 | 11160 | 648 | 184 | 335 | 494 |
| 2006 | 2487 | 787 | 4276 | 1970 | 9669 | 11620 | 701 | 156 | 344 | 454 |
| 2007 | 2508 | 866 | 4445 | 2095 | 10055 | 11937 | 695 | 181 | 419 | 516 |
| 2008 | 2426 | 739 | 4437 | 2071 | 9419 | 11380 | 672 | 130 | 580 | 545 |
| 2009 | 2518 | 803 | 4412 | 2018 | 9589 | 10598 | 827 | 199 | 847 | 601 |
| Total | 77762 | 32538 | 77438 | 76946 | 300249 | 299851 | 28535 | 11265 | -50597 | 23146 |

The Table shows the increases in the annual number of deaths that would have occurred in Swedish males aged 30-79 years if they had had the average mortality of 11 countries with a prevalence of cigarette consumption similar to the prevalence of tobacco consumption seen in Sweden. This is estimated using Approach 1. The adjustment is based on the lower mortality from NRSD in Sweden.

**Table 2. Increases in deaths in Sweden if snus had not been introduced – Approach 2**

|  | **Increase in Deaths** | | | |
| --- | --- | --- | --- | --- |
| **Year** | **Lung Cancer** | **COPD** | **IHD** | **Stroke** |
| 1980 | 262 | 78 | 472 | 57 |
| 1981 | 247 | 72 | 466 | 59 |
| 1982 | 237 | 68 | 442 | 54 |
| 1983 | 238 | 65 | 422 | 52 |
| 1984 | 249 | 61 | 413 | 50 |
| 1985 | 242 | 65 | 419 | 52 |
| 1986 | 234 | 60 | 406 | 50 |
| 1987 | 236 | 51 | 388 | 49 |
| 1988 | 240 | 54 | 376 | 51 |
| 1989 | 225 | 50 | 342 | 47 |
| 1990 | 235 | 54 | 343 | 52 |
| 1991 | 247 | 51 | 336 | 57 |
| 1992 | 243 | 50 | 322 | 56 |
| 1993 | 237 | 54 | 327 | 56 |
| 1994 | 252 | 52 | 320 | 57 |
| 1995 | 257 | 52 | 331 | 51 |
| 1996 | 261 | 54 | 308 | 59 |
| 1997 | 276 | 55 | 313 | 59 |
| 1998 | 277 | 55 | 303 | 60 |
| 1999 | 296 | 57 | 308 | 63 |
| 2000 | 298 | 57 | 307 | 60 |
| 2001 | 310 | 55 | 306 | 60 |
| 2002 | 321 | 61 | 296 | 63 |
| 2003 | 347 | 59 | 304 | 62 |
| 2004 | 363 | 59 | 300 | 63 |
| 2005 | 403 | 63 | 304 | 59 |
| 2006 | 407 | 62 | 296 | 66 |
| 2007 | 418 | 67 | 316 | 62 |
| 2008 | 463 | 71 | 315 | 65 |
| 2009 | 465 | 70 | 307 | 66 |
| Total | 8786 | 1781 | 10409 | 1720 |

The Table shows the increases in the annual number of deaths that would have occurred in Swedish males aged 30-79 years if those who used snus had smoked instead. This is estimated using Approach 2.

**Table 3. Increases in deaths in Sweden if snus had not been introduced – Approach 3 (varying f-factor)**

|  | **Lung Cancer** | | | **COPD** | | | **IHD** | | | **Stroke** | | |
| --- | --- | --- | --- | --- | --- | --- | --- | --- | --- | --- | --- | --- |
|  | **f-factor** | | | | | | | | | | | |
| **Year** | **0** | **0.1** | **0.2** | **0** | **0.1** | **0.2** | **0** | **0.1** | **0.2** | **0** | **0.1** | **0.2** |
| 1980 | 235 | 229 | 222 | 89 | 86 | 84 | 570 | 556 | 543 | 64 | 63 | 61 |
| 1981 | 223 | 217 | 211 | 82 | 80 | 78 | 551 | 537 | 523 | 63 | 62 | 60 |
| 1982 | 211 | 205 | 200 | 76 | 74 | 72 | 522 | 509 | 495 | 58 | 57 | 55 |
| 1983 | 206 | 200 | 195 | 70 | 68 | 66 | 494 | 480 | 467 | 56 | 54 | 52 |
| 1984 | 216 | 209 | 203 | 66 | 64 | 62 | 479 | 465 | 451 | 53 | 51 | 50 |
| 1985 | 207 | 200 | 194 | 69 | 67 | 65 | 476 | 461 | 446 | 54 | 53 | 51 |
| 1986 | 195 | 188 | 182 | 62 | 60 | 58 | 456 | 440 | 424 | 52 | 50 | 48 |
| 1987 | 201 | 194 | 187 | 54 | 52 | 50 | 442 | 426 | 409 | 52 | 49 | 47 |
| 1988 | 214 | 206 | 198 | 61 | 59 | 57 | 440 | 422 | 404 | 54 | 52 | 49 |
| 1989 | 196 | 188 | 180 | 55 | 53 | 51 | 397 | 380 | 362 | 50 | 48 | 46 |
| 1990 | 206 | 197 | 188 | 59 | 57 | 55 | 391 | 372 | 354 | 54 | 51 | 48 |
| 1991 | 216 | 206 | 196 | 55 | 53 | 50 | 386 | 366 | 346 | 60 | 56 | 53 |
| 1992 | 216 | 205 | 195 | 55 | 53 | 51 | 374 | 354 | 333 | 59 | 55 | 52 |
| 1993 | 216 | 204 | 193 | 63 | 60 | 58 | 386 | 364 | 342 | 59 | 56 | 52 |
| 1994 | 228 | 215 | 202 | 59 | 56 | 53 | 379 | 355 | 332 | 62 | 58 | 54 |
| 1995 | 227 | 214 | 200 | 57 | 54 | 51 | 391 | 366 | 341 | 56 | 52 | 48 |
| 1996 | 236 | 221 | 207 | 59 | 56 | 53 | 374 | 349 | 324 | 66 | 61 | 57 |
| 1997 | 251 | 234 | 218 | 61 | 58 | 54 | 383 | 357 | 331 | 66 | 61 | 56 |
| 1998 | 249 | 231 | 215 | 61 | 57 | 54 | 370 | 342 | 316 | 67 | 62 | 57 |
| 1999 | 269 | 250 | 231 | 62 | 58 | 55 | 381 | 353 | 324 | 71 | 66 | 60 |
| 2000 | 270 | 250 | 231 | 62 | 58 | 54 | 380 | 350 | 321 | 68 | 63 | 57 |
| 2001 | 291 | 269 | 247 | 61 | 57 | 54 | 387 | 357 | 327 | 69 | 63 | 58 |
| 2002 | 300 | 276 | 253 | 68 | 63 | 59 | 375 | 344 | 315 | 72 | 66 | 61 |
| 2003 | 320 | 293 | 267 | 64 | 59 | 55 | 383 | 350 | 319 | 71 | 65 | 59 |
| 2004 | 342 | 313 | 285 | 66 | 62 | 57 | 384 | 351 | 319 | 74 | 68 | 62 |
| 2005 | 374 | 342 | 311 | 70 | 65 | 60 | 382 | 349 | 318 | 68 | 62 | 56 |
| 2006 | 385 | 350 | 317 | 71 | 65 | 60 | 379 | 347 | 315 | 77 | 71 | 64 |
| 2007 | 387 | 352 | 318 | 76 | 70 | 64 | 401 | 365 | 331 | 72 | 66 | 60 |
| 2008 | 422 | 382 | 345 | 78 | 71 | 65 | 400 | 364 | 329 | 77 | 70 | 64 |
| 2009 | 422 | 381 | 343 | 78 | 72 | 66 | 390 | 355 | 321 | 78 | 71 | 64 |
| Total | 7931 | 7423 | 6932 | 1969 | 1868 | 1770 | 12501 | 11784 | 11082 | 1901 | 1781 | 1663 |

The Table shows the increases in the annual number of deaths that would have occurred in Swedish males aged 30-79 years if those who used snus had smoked instead. This is estimated using Approach 3. The analyses assume the g-factor is 1, but the f-factor is allowed to vary.

**Table 4. Increases in deaths in Sweden if snus had not been introduced – Approach 3 (varying g-factor)**

|  | **Lung Cancer** | | | **COPD** | | | **IHD** | | | **Stroke** | | |
| --- | --- | --- | --- | --- | --- | --- | --- | --- | --- | --- | --- | --- |
|  | **g-factor** | | | | | | | | | | | |
| **Year** | **0.9** | **0.8** | **0.5** | **0.9** | **0.8** | **0.5** | **0.9** | **0.8** | **0.5** | **0.9** | **0.8** | **0.5** |
| 1980 | 248 | 260 | 298 | 93 | 97 | 109 | 599 | 628 | 715 | 67 | 71 | 80 |
| 1981 | 235 | 247 | 284 | 86 | 90 | 102 | 580 | 609 | 698 | 67 | 70 | 80 |
| 1982 | 223 | 235 | 272 | 80 | 84 | 95 | 550 | 578 | 662 | 61 | 65 | 74 |
| 1983 | 218 | 230 | 268 | 74 | 78 | 89 | 521 | 548 | 630 | 59 | 62 | 71 |
| 1984 | 228 | 241 | 281 | 69 | 73 | 84 | 505 | 532 | 612 | 56 | 58 | 67 |
| 1985 | 220 | 233 | 273 | 73 | 77 | 89 | 502 | 529 | 611 | 57 | 60 | 69 |
| 1986 | 207 | 220 | 259 | 66 | 69 | 81 | 482 | 508 | 587 | 55 | 58 | 66 |
| 1987 | 214 | 226 | 266 | 57 | 60 | 70 | 467 | 492 | 567 | 54 | 57 | 65 |
| 1988 | 227 | 240 | 281 | 64 | 68 | 79 | 463 | 487 | 560 | 57 | 60 | 68 |
| 1989 | 209 | 221 | 260 | 58 | 61 | 71 | 419 | 440 | 505 | 53 | 55 | 63 |
| 1990 | 219 | 232 | 272 | 63 | 66 | 77 | 412 | 434 | 499 | 57 | 59 | 68 |
| 1991 | 229 | 243 | 285 | 58 | 62 | 72 | 406 | 427 | 490 | 63 | 66 | 75 |
| 1992 | 229 | 243 | 284 | 59 | 62 | 72 | 394 | 413 | 473 | 62 | 65 | 74 |
| 1993 | 228 | 241 | 281 | 67 | 70 | 81 | 406 | 426 | 486 | 62 | 65 | 74 |
| 1994 | 241 | 254 | 296 | 62 | 65 | 75 | 398 | 417 | 476 | 65 | 67 | 76 |
| 1995 | 241 | 254 | 297 | 60 | 64 | 74 | 410 | 430 | 490 | 58 | 61 | 69 |
| 1996 | 250 | 264 | 307 | 62 | 66 | 76 | 391 | 409 | 465 | 69 | 72 | 80 |
| 1997 | 265 | 279 | 324 | 64 | 68 | 78 | 401 | 420 | 475 | 69 | 71 | 80 |
| 1998 | 263 | 277 | 322 | 64 | 68 | 78 | 387 | 404 | 458 | 70 | 73 | 82 |
| 1999 | 284 | 299 | 346 | 66 | 69 | 80 | 399 | 416 | 469 | 74 | 77 | 86 |
| 2000 | 285 | 300 | 348 | 65 | 69 | 79 | 397 | 414 | 466 | 71 | 73 | 82 |
| 2001 | 306 | 322 | 370 | 65 | 68 | 77 | 404 | 421 | 472 | 72 | 75 | 83 |
| 2002 | 316 | 332 | 382 | 71 | 75 | 85 | 391 | 407 | 457 | 75 | 78 | 87 |
| 2003 | 337 | 354 | 407 | 67 | 70 | 80 | 399 | 415 | 465 | 74 | 77 | 85 |
| 2004 | 359 | 376 | 431 | 69 | 72 | 82 | 399 | 415 | 463 | 77 | 80 | 89 |
| 2005 | 393 | 412 | 471 | 73 | 76 | 86 | 398 | 413 | 461 | 70 | 73 | 81 |
| 2006 | 403 | 423 | 483 | 74 | 77 | 87 | 394 | 410 | 456 | 80 | 83 | 92 |
| 2007 | 406 | 426 | 488 | 79 | 83 | 94 | 417 | 433 | 482 | 75 | 77 | 85 |
| 2008 | 443 | 464 | 532 | 81 | 85 | 96 | 415 | 430 | 478 | 79 | 82 | 91 |
| 2009 | 443 | 464 | 531 | 82 | 85 | 96 | 405 | 420 | 467 | 80 | 83 | 92 |
| Total | 8367 | 8812 | 10198 | 2072 | 2175 | 2493 | 13110 | 13724 | 15597 | 1986 | 2072 | 2333 |

The Table shows the increases in the annual number of deaths that would have occurred in Swedish males aged 30-79 years if those who used snus had smoked instead. This is estimated using Approach 3. The analyses assume the f-factor is 0, but the g-factor is allowed to vary.

**Table 5. Increases in deaths in Sweden if snus had not been introduced**

**– Approach 3 (varying g-factor with f-factor = 0.1)**

|  | **Lung Cancer** | | **COPD** | | **IHD** | | **Stroke** | |
| --- | --- | --- | --- | --- | --- | --- | --- | --- |
|  | **g-factor** | | | | | | | |
| **Year** | **0.9** | **1.1** | **0.9** | **1.1** | **0.9** | **1.1** | **0.9** | **1.1** |
| 1980 | 241 | 217 | 90 | 82 | 585 | 528 | 66 | 60 |
| 1981 | 229 | 205 | 84 | 76 | 566 | 508 | 65 | 58 |
| 1982 | 217 | 194 | 78 | 70 | 536 | 481 | 60 | 54 |
| 1983 | 212 | 188 | 72 | 65 | 507 | 453 | 57 | 51 |
| 1984 | 222 | 197 | 67 | 60 | 491 | 439 | 54 | 48 |
| 1985 | 213 | 188 | 71 | 63 | 487 | 434 | 56 | 50 |
| 1986 | 200 | 176 | 64 | 56 | 466 | 414 | 53 | 47 |
| 1987 | 206 | 181 | 55 | 49 | 450 | 401 | 52 | 47 |
| 1988 | 219 | 193 | 62 | 55 | 446 | 398 | 54 | 49 |
| 1989 | 200 | 176 | 56 | 50 | 401 | 359 | 50 | 45 |
| 1990 | 209 | 184 | 60 | 53 | 393 | 351 | 54 | 48 |
| 1991 | 219 | 193 | 56 | 49 | 386 | 345 | 59 | 53 |
| 1992 | 218 | 193 | 56 | 50 | 373 | 334 | 58 | 53 |
| 1993 | 217 | 192 | 64 | 57 | 383 | 345 | 59 | 53 |
| 1994 | 228 | 202 | 59 | 52 | 374 | 337 | 61 | 55 |
| 1995 | 227 | 201 | 57 | 51 | 385 | 346 | 55 | 49 |
| 1996 | 235 | 208 | 59 | 52 | 366 | 331 | 64 | 58 |
| 1997 | 248 | 221 | 61 | 54 | 374 | 339 | 64 | 58 |
| 1998 | 245 | 218 | 61 | 54 | 359 | 326 | 65 | 59 |
| 1999 | 264 | 235 | 62 | 55 | 369 | 336 | 69 | 63 |
| 2000 | 265 | 236 | 61 | 55 | 367 | 334 | 65 | 60 |
| 2001 | 283 | 254 | 61 | 54 | 373 | 340 | 66 | 61 |
| 2002 | 291 | 261 | 67 | 60 | 360 | 329 | 69 | 64 |
| 2003 | 309 | 277 | 63 | 56 | 366 | 335 | 68 | 62 |
| 2004 | 329 | 297 | 64 | 59 | 366 | 336 | 71 | 65 |
| 2005 | 359 | 324 | 68 | 62 | 364 | 335 | 65 | 59 |
| 2006 | 368 | 333 | 68 | 62 | 361 | 332 | 73 | 68 |
| 2007 | 370 | 334 | 73 | 67 | 381 | 350 | 68 | 63 |
| 2008 | 402 | 363 | 75 | 68 | 379 | 349 | 73 | 67 |
| 2009 | 401 | 362 | 75 | 69 | 369 | 340 | 73 | 68 |
| Total | 7849 | 7004 | 1970 | 1767 | 12386 | 11186 | 1865 | 1697 |

The Table shows the increases in the annual number of deaths that would have occurred in Swedish males aged 30-79 years if those who used snus had smoked instead. This is estimated using Approach 3. The analyses assume the f-factor is 0.1, but the g-factor is allowed to vary.

**Table 6. Increases in deaths in Sweden if snus had not been introduced –**

**Approach 3 with f = 0, g = 0.1 but survival-adjusted**

| **Year** | **Lung Cancer** | **COPD** | **IHD** | **Stroke** |
| --- | --- | --- | --- | --- |
| 1980 | 235 | 89 | 570 | 64 |
| 1981 | 222 | 82 | 539 | 61 |
| 1982 | 209 | 75 | 501 | 54 |
| 1983 | 203 | 68 | 464 | 49 |
| 1984 | 211 | 63 | 441 | 45 |
| 1985 | 201 | 65 | 430 | 45 |
| 1986 | 188 | 58 | 404 | 41 |
| 1987 | 193 | 50 | 388 | 40 |
| 1988 | 205 | 56 | 381 | 41 |
| 1989 | 188 | 50 | 339 | 36 |
| 1990 | 196 | 54 | 327 | 38 |
| 1991 | 205 | 49 | 322 | 42 |
| 1992 | 206 | 50 | 308 | 42 |
| 1993 | 204 | 56 | 319 | 42 |
| 1994 | 215 | 52 | 312 | 43 |
| 1995 | 215 | 49 | 321 | 37 |
| 1996 | 223 | 51 | 307 | 47 |
| 1997 | 236 | 53 | 316 | 45 |
| 1998 | 234 | 53 | 304 | 46 |
| 1999 | 253 | 54 | 315 | 50 |
| 2000 | 255 | 53 | 318 | 46 |
| 2001 | 275 | 53 | 327 | 50 |
| 2002 | 284 | 59 | 315 | 52 |
| 2003 | 303 | 55 | 324 | 51 |
| 2004 | 324 | 58 | 328 | 55 |
| 2005 | 355 | 61 | 327 | 50 |
| 2006 | 366 | 62 | 325 | 60 |
| 2007 | 367 | 67 | 348 | 55 |
| 2008 | 401 | 68 | 347 | 59 |
| 2009 | 401 | 69 | 340 | 60 |
| Total | 7573 | 1781 | 10907 | 1443 |

The Table shows the increases in the annual numbers of deaths that would have occurred in Swedish males aged 30-79 years if those who used snus had smoked instead. This is estimated using Approach 2.
